# Understanding EMS response times: a machine learning-based analysis

**DOI:** 10.1186/s12911-025-02975-z

**Published:** 2025-03-24

**Authors:** Peter Hill, Jakob Lederman, Daniel Jonsson, Peter Bolin, Veronica Vicente

**Affiliations:** 1 Region Stockholm Health and Medical Care Administration, The Department for Specialized Care, Stockholm, Sweden; 2 The Ambulance Medical Service in Stockholm (AISAB), Stockholm, Sweden; 3 Academic EMS in Stockholm, Stockholm, Sweden; 4 Karolinska Institutet, Department of Clinical Science and Education, Södersjukhuset in Stockholm, Stockholm, SE-118 83 Sweden; 5 KTH Royal Institute of Technology, The Department of Urban Planning and Environment, Stockholm, Sweden; 6 Karolinska Institutet, Department of Learning, Informatics, Management and Ethics (LIME), Solna, Sweden

**Keywords:** Emergency medical services, Response times, Machine learning, Resource allocation, Predictive analytics, Emergency care optimization

## Abstract

**Background:**

Emergency Medical Services (EMS) response times are critical for optimizing patient outcomes, particularly in time-sensitive emergencies. This study explores the multifaceted determinants of EMS response times, leveraging machine learning (ML) techniques to identify key factors such as urgency levels, environmental conditions, and geographic variables. The findings aim to inform strategies for enhancing resource allocation and operational efficiency in EMS systems.

**Methods:**

A retrospective analysis was conducted using over one million EMS missions from Stockholm, Sweden, between 2017 and 2022. Advanced ML techniques, including Gradient Boosting models, were applied to evaluate the influence of diverse variables such as call handling times, travel times, weather patterns, and resource availability. Feature engineering was employed to extract meaningful insights, and statistical models were used to validate the relationships between key predictors and response times.

**Results:**

The study revealed a complex interplay of factors influencing EMS response times, aligning with the study’s aim to deepen the understanding of these determinants. Key drivers of response time variability included weather conditions, call priority, and resource constraints. ML models, particularly Gradient Boosting, proved effective in quantifying these impacts and provided robust predictions of response times across scenarios. By providing a comprehensive evaluation of these influences, the results support the development of adaptive resource allocation models and evidence-based policies aimed at enhancing EMS efficiency and equity across all call priorities.

**Conclusions:**

This study underscores the potential of ML-driven insights to revolutionize EMS resource allocation strategies. By integrating real-time data on weather, call types, and workload, EMS systems can transition to adaptive deployment models, reducing response times and enhancing equity across priority levels. The research provides a blueprint for implementing predictive analytics in EMS operations, paving the way for evidence-based policies that improve emergency care efficiency and outcomes.

**Clinical trial number:**

Not applicable.

**Supplementary Information:**

The online version contains supplementary material available at 10.1186/s12911-025-02975-z.

## Background

Emergency medical services (EMS), integral to out-of-hospital emergency care, utilize specialized vehicles and exhibit a notable degree of organizational similarity across different countries [1, 2]. The EMS process begins when an emergency call is received by the Emergency Medical Communication Centre (EMCC), where an Emergency Medical Dispatcher (EMD) assigns a priority based on the perceived risk of the patient having a time-critical medical condition [1, 3]. In Sweden, there are three priority levels: Priority 1, indicating the highest urgency, followed by Priorities 2 and 3 for lower levels, with lower-priority cases being deferred if resources are limited [3].

Accurately assessing the urgency of emergency calls is challenging due to factors such as language barriers and caller agitation. Emergency calls are rarely made by patients themselves and are often made by relatives or bystanders, leading to discrepancies between the urgency assigned by the EMCC and the clinical assessment performed by EMS personnel upon arrival. These discrepancies can result in misjudgment, potentially impacting patient outcomes [4–8].

Response time, defined as the interval from the initiation of an emergency call to the arrival of EMS personnel on the scene, is a globally recognized metric for evaluating EMS performance, particularly in high-urgency cases where lights and sirens are used. It comprises two phases: call handling time, from receipt of the call to the dispatch of resources, and travel time, from resource deployment to arrival at the scene [9]. Delays in response time can affect patient outcomes, making it a critical factor in EMS resource planning. Resource shortages in specific geographic areas often exacerbate response times [9–12]. In Sweden, the Swedish Association of Local Authorities and Regions (SKR) employs the median response time for Priority 1 calls as a benchmark for comparative assessment across EMS systems nationwide [13].

Despite extensive research on EMS resource allocation and optimization models, gaps remain in understanding how various factors interact to influence response times. Real-time traffic data, historical trends, and dynamic resource allocation strategies, such as “fluid deployment,” have shown potential for reducing response times. However, many studies have focused on isolated factors like traffic or weather without accounting for their combined effects or the disparities between urgency levels [6, 14–29].

While using lights and sirens accelerates response and transportation times, it poses risks of vehicular accidents and threats to public safety. Evidence supporting response times direct positive impact on patient outcomes remains limited [30]. Additionally, factors such as weather conditions and temporal variations, can impact response times, patient volumes, and resource deployment, further complicating resource allocation strategies [31–42].

### Advances in machine learning in EMS systems and research gap

Recent advancements in data collection and machine learning (ML) have significantly transformed Emergency Medical Services (EMS) operations. ML technologies have enhanced operational efficiency, minimized response times, and improved patient outcomes. Contemporary research underscores the critical roles of ML in EMS systems, particularly in demand prediction, resource optimization, and the integration of environmental and temporal factors into planning frameworks.

Machine learning models have demonstrated remarkable capabilities in several domains. They effectively forecast EMS demand by leveraging spatio-temporal data, traffic patterns, and even insights from social media [21, 26, 43]. Bayesian models, dynamic relocation strategies, and robust optimization algorithms have improved resource allocation, directly reducing response times [23, 44–46]. Additionally, regression-based models, including gradient boosting techniques, estimate response times with high accuracy, factoring in external variables such as weather and traffic conditions [6, 10, 39].

However, despite these strides, significant challenges and research gaps remain. Most studies have focused on individual factors like traffic or weather, neglecting their combined effects on EMS efficiency [6, 10, 21, 25, 27, 39]. Moreover, the scalability of ML models across different geographic and operational contexts is underexplored [23, 44–47]. Practical implementation of real-time ML predictions in live EMS operations also poses logistical and computational hurdles, especially during high-risk situations [6, 10, 39].

Addressing these gaps, the current study introduces an integrative ML framework that combines various factors such as urgency levels, geographic contexts, and weather conditions to provide a holistic analysis of EMS response dynamics. Unlike prior research, this approach not only evaluates the interplay of these variables but also proposes equitable strategies for resource allocation across different priority levels, emphasizing both high-priority and lower-priority cases.

### Outline of the paper

This paper begins by introducing the current state of knowledge regarding EMS response times, emphasizing both their significance in clinical outcomes and the limitations of solely using traditional metrics such as high-priority response times. It then explains how machine learning techniques can integrate various factors—urgent call assessments, dynamic resource deployment, and environmental variables—to develop a more holistic understanding of EMS operations. Subsequently, the data sources and methodological framework are discussed, highlighting the retrospective observational design, over one million EMS missions included from Stockholm between 2017 and 2022, the use of feature engineering, and the application of both linear regression and advanced machine learning methods (particularly gradient boosting). The analysis then focuses on the study’s main findings, demonstrating how urgency levels, weather conditions, and geographic factors collectively influence EMS response times, while also comparing model performance across different call priorities. This leads into a broader discussion that contextualizes these results within existing literature, explores their potential implications for resource allocation and policy, and addresses the feasibility and challenges of real-time machine learning implementation in EMS. Lastly, the paper concludes by underscoring key contributions, proposing strategies for more equitable and efficient EMS management, and outlining directions for further research, including the integration of missing data sources such as real-time traffic information, refined model deployment, and applicability to other regions.

### Aim

This study aims to deepen the understanding of the complex factors influencing EMS response times. By examining the interplay between urgency levels, environmental conditions, and geographical contexts, it seeks to uncover actionable insights that can optimize resource allocation and enhance the effectiveness of emergency response operations.

## Materials and methods

### Study design

This study utilizes a retrospective observational design, leveraging an extensive dataset of over one million emergency medical service (EMS) missions conducted in Stockholm, Sweden, from 2017 to 2022. The Strengthening the Reporting of Observational Studies in Epidemiology (STROBE) checklist, designed to assist authors in presenting observational studies with clarity and rigor, was followed to ensure comprehensive and transparent reporting [48]. The invaluable EMS procedural data were sourced from the Region Stockholm VAL databases, which are renowned for their robust and cohesive data at the individual level [49, 50]. Furthermore, to enrich our analysis, meteorological data from the Swedish Meteorological and Hydrological Institute were seamlessly integrated to provide invaluable contextual insights [7, 50, 51]. For analytical rigor, the study employed diverse statistical methods, including linear regression and advanced machine learning models, supported by feature engineering to incorporate key variables influencing EMS response time. A directed acyclic graph (DAG) was constructed to visually represent factors partially assumed to be influencing response time, elucidating potential causal pathways. To enhance the understanding of the multifaceted factors influencing EMS response time and their interrelationships, data analyses were conducted via a diverse array of statistical methodologies, including both traditional linear regression and advanced machine learning models [52–54]. These analyses were meticulously synthesised, presenting a comprehensive blend of inferential and descriptive statistics. To increase the analytical depth, sophisticated data mining techniques and feature engineering have been employed to identify and incorporate variables known to significantly influence response time [8, 9, 14, 31–37, 39, 40, 42, 55]. The program code used for feature engineering and machine learning is provided in Appendix B.

### Setting of the study

The study was conducted in Stockholm, a metropolitan area with a population of approximately 2.5 million inhabitants [56]. Healthcare services in Stockholm, including EMSs, are overseen by Region Stockholm [56]. The EMCC is operated by SOS Alarm AB, a publicly owned entity. EMS operations are carried out by a combination of private and publicly owned operators contracted by Region Stockholm [56, 57]. The EMS resources are stationed at fixed stations with the option of “fluid deployed” [9, 58–60].

### Participants

The initial dataset comprises a total of *N* = 1 297 858 observations, encompassing all EMS missions conducted in Stockholm between 2017 and 2022. Employing the exclusion criteria outlined in Table 1, the dataset underwent a meticulous refinement process, resulting in a refined subset of *n* = 1 144 754 observations deemed appropriate for subsequent feature engineering and in-depth analysis.

**Table 1:** Inclusion and exclusion criteria

Initial dataset *N* = 1 297 858 observations	
Exclusion criteria	Number of excluded observations
Dispatch priority not 1, 2, or 3 (Transport assignments)	***n*** ** = 480 710**
EMS unit type not Emergency Ambulance	
Year not between 2017 and 2022	
Response time not between 0 and 10 h*	
Call handling time not between 0 and 10 h*	
Travel time not between 0 and 10 h*	
On scene time not between 0 and 10 h*	
Transportation time not between 0 and 10 h*	
Delivery time not between 0 and 10 h*	
Intrahospital transports	
**Observations included** *** n*** ** = 817 148 observations**	

* Observations with skewed timestamps caused by technical issues in the prehospital digital platform FRAPP were excluded. Specifically, data with negative time values or response times exceeding 10 h were considered highly unlikely on the basis of the authors’ domain expertise and were therefore omitted from the analysis

### Variables and features

**Fig. 1:**
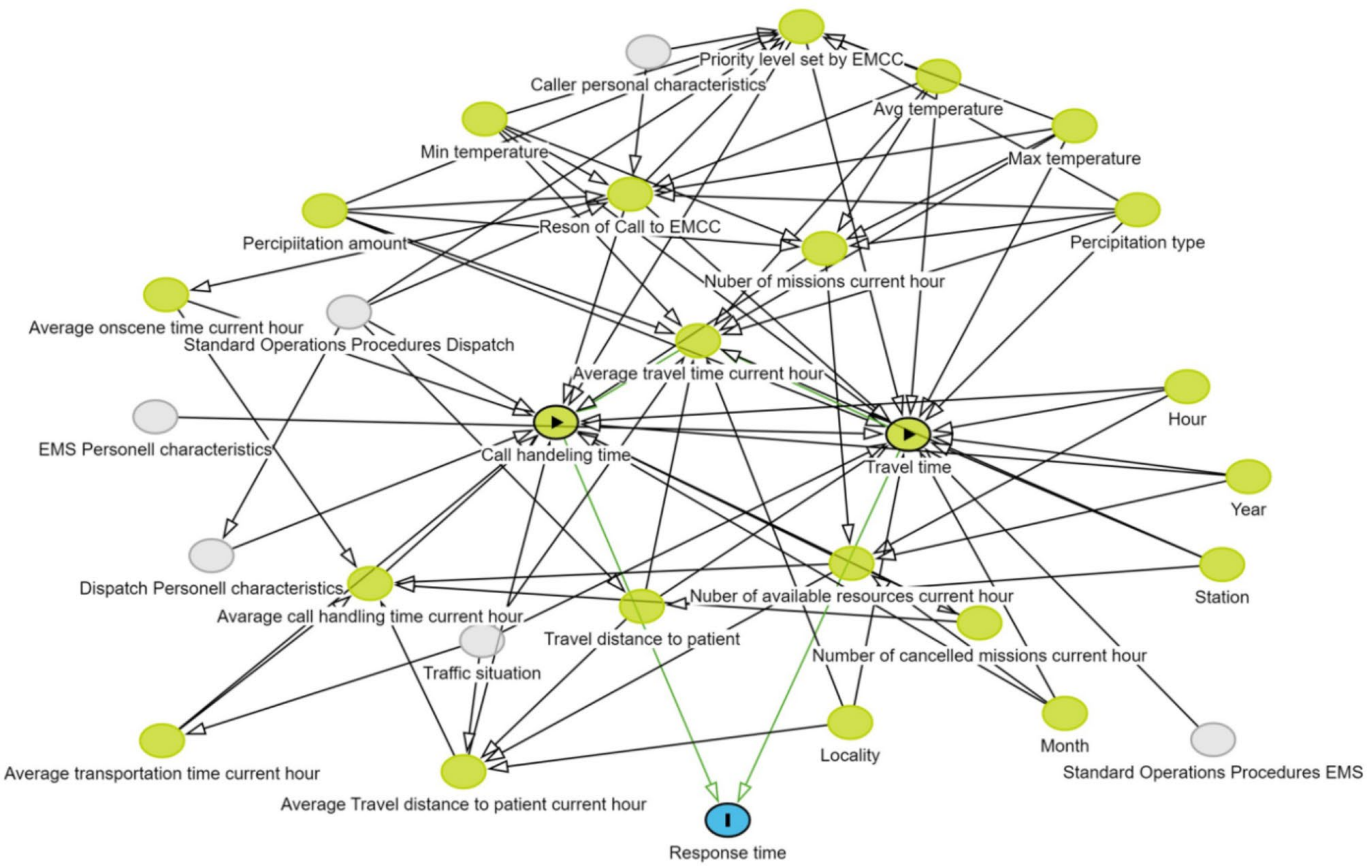
Depicts a directed acyclic graph illustrating the relationships between exposures and the outcome, indicating the absence of any open biasing path (60). The features highlighted in green represent variables that were included in the study

A DAG illustrated the hypothesized factors influencing response time and their measurable features. The DAG highlights the variables (in green) that were quantifiable either directly or by combining multiple variables (Fig. 1).

### Feature engineering and utilisation in data analysis

The feature engineering process in this study was designed to extract meaningful insights from a complex dataset comprising over one million EMS missions. By transforming raw data into actionable features, the analysis incorporated diverse factors influencing response times, including urgency levels, geographical variables, and environmental conditions.

Continuous variables were standardized to a mean of 0 and a standard deviation of 1 to facilitate linear regression [54]. This standardization ensured comparability across variables with different scales, improving the stability and interpretability of the model. Missing data were incorporated into machine learning models as predictors, acknowledging that missingness itself can contribute valuable insights by reflecting systemic strain or resource availability. For instance, missing timestamps were included as indicators of data flow issues or operational delays.

Notably, the dataset excluded extreme outliers, such as response times exceeding 10 h, which were deemed operationally implausible based on expert knowledge (by object specialist of the data source at Region Stockholm) and technical reviews of timestamp errors. This exclusion ensured that the analysis focused on plausible scenarios while maintaining model reliability.

The features for the machine learning models were designed to align with the specific requirements outlined in the DAG. Key considerations and methodologies included variable grouping, where the original dataset containing 333 categories for call reasons and 115 localities was grouped into 54 and 48 broader categories, respectively. These groupings reduced dimensionality while preserving meaningful distinctions, based on similarities in call type characteristics and geographic proximities to ensure operational relevance and interpretability (Table 2).

Derived features were also created to capture temporal and system-wide dynamics. Hourly averages of response times, call handling times, and travel distances were calculated to reflect system strain during peak periods. Weather-related features, such as temperature and precipitation, were integrated to evaluate their impact on EMS operations. Resource utilization metrics, including the hourly sum of missions and cancellations, provided insights into workload effects.

Additionally, gradient boosting models were used to assess the relative importance of features. Key determinants, such as Emergency Medical Communication Centre (EMCC) priority levels and weather conditions, aligned with prior research and expert judgment. Interaction effects, such as those between locality and adverse weather, were modelled to capture their combined influence on response times, offering a deeper understanding of these interdependencies.

This comprehensive approach ensured that the models effectively represented the complexities of EMS operations, enabling robust predictions and actionable insights.

**Table 2:** Explanation of crafted feature

Feature	Explanation
Priority level set by EMCC	The priority level set by EMCC is presumed to affect response time due to the assessed urgency. Since all missions in all priority levels affect response time between priority levels, stratification between priority levels is not suitable. Instead, the priority level is handled as a feature affecting response time like the other variables investigated in this study.
Response time	A created feature using the time difference between when the EMCC call was received, and the first EMS resource arrival at scene of incident. The response time is a result of call handling time and travel time.
Call Handling time	A created feature using the time difference between when the EMCC call was received, and the first EMS resource received a mission from EMCC in the incident.
Travel time	A created feature using the time difference between when the first EMS resource received a mission from EMCC in the incident, and the first EMS resource arrival at scene of incident.
Reason of Call to EMCC	The reason of call to EMCC was grouped from the origin of 333 different categories, which differs slightly between different years, into 54 different categories reflecting the reason of call to EMCC. Reason of Call to EMCC is presumed to effect call handling time because differences in call handling time due to the nature of different reason of call to EMCC-categories. Reason of Call to EMCC is presumed to effect travel time because differences in travel time in different reason of call to EMCC-categories where personal EMS-personnel experience might influence travel time.
Locality	The locality was grouped from the origin of 115 localities, where the same localities was duplicated one ore several times due to misspelling, into 48 localities which reflect the real locality. Locality is presumed to affect call handling time due to amount of available resources might differ in different localities. If there is lack of resources in a locality the call handling time might increase due to finding nearest suitable resource. Locality is presumed to affect travel distance and travel time because different coverage of available resources in different localities. Differences related to locality might indicate EMS allocation out of tune.
Hour	The hour of day is used to investigate differences in response time between hour of day. Differences related to hour might indicate EMS allocation out of tune.
Month	The month of year is used to investigate differences between month of year. Differences related to month might indicate EMS allocation out of tune.
Year	The year is used to identify differences in years like the Covid-19 pandemic and differences in EMS allocation between years. Differences between years might indicate EMS allocation out of tune.
On scene time	A created feature using the time difference between when the first EMS resource arrival at scene of incident and the first EMS resource started transportation of patient.
Transportation time	A created feature using the time difference between when the first EMS resource started transportation of patient and the first EMS resource arrival at hospital.
Air temperature	The average temperature current day at the weather station called “Observatoriekullen” in the centre of Stockholm. Differences in temperature is presumed to affect response time in different ways, like low temperature in combination with precipitation could affect traffic and travel time. It could also generate differences in reason of call to EMCC related to high and low temperatures.
Airtemperature_max	The max temperature current day at the weather station called “Observatoriekullen” in the centre of Stockholm. Differences in max temperature is presumed to affect response time in different ways, like low temperature in combination with precipitation could affect traffic and travel time. It could also generate differences in reason of call to EMCC related to high and low temperatures.
Airtemperature_min	The min temperature current day at the weather station called “Observatoriekullen” in the centre of Stockholm. Differences in min temperature is presumed to affect response time in different ways, like low temperature in combination with precipitation could affect traffic and travel time. It could also generate differences in reason of call to EMCC related to high and low temperatures.
H_AVG_of_Responsetime	A created feature aggregating the response time as average response time of all missions current hour. The purpose is to reflect how the general response time the current hour effects response time in individual missions. Differences in the hourly average response time is presumed to reflect the system where increased hourly response time might indicate strained situation or EMS allocation out of tune.
H_AVG_of_Call_Handlingtime	A created feature aggregating the call handling time as average response time of all missions current hour. The purpose is to reflect how the general call handling time the current hour effects response time in individual missions. Differences in the hourly average call handling time is presumed to reflect the system where increased hourly call handling time might indicate strained situation or EMS allocation out of tune.
H_AVG_of_DistArrival_km	A created feature aggregating the travel distance to patient in all missions current hour. The purpose is to reflect how the general travel distance to patient the current hour effects response time in individual missions. Differences in the hourly average travel distance is presumed to reflect the system where increased travel distance might indicate strained situation or EMS allocation out of tune.
H_AVG_of_Drivetime	A created feature aggregating the drive time as average drive time of all missions current hour. The purpose is to reflect how the general drive time the current hour effects response time in individual missions. Differences in the drive time is presumed to reflect the system where increased travel distance might indicate strained situation or EMS allocation out of tune. The traffic situation is a factor highly affecting drive time.
H_AVG_of_Onscenetime	A created feature aggregating the on scene time as average on scene time of all missions current hour. The purpose is to reflect how the general on scene time the current hour effects response time in individual missions. Differences in the on scene time is presumed to indicate prolonged treatment time affecting the response time.
H_AVG_of_Transportationtime	A created feature aggregating the on transportation time as average transportation time of all missions current hour. The purpose is to reflect how the general transportation time the current hour effects response time in individual missions. Differences in the on scene time is presumed to indicate prolonged transportation time affecting the response time.
H_SumOfTasks	A created feature aggregating the sum of missions current hour. The purpose is to reflect how the sum of missions the current hour effects response time in individual missions. Differences in the sum of missions is presumed in relation to available resources is presumed affecting the response time.
H_Sum_of_MissionCancelled	A created feature aggregating the sum of cancelled missions the current hour. The purpose is to reflect how the sum of cancelled missions the current hour effects response time in individual missions. Differences in the sum of cancelled missions is presumed to reflect the system, where a strained situation with lack of resources, or EMS allocation out of tune, would give a higher rate of cancelled missions and affect the response time.
H_C_DIST_of_rakelid	A created feature aggregating the sum of distinct EMS resource ID the current hour. The purpose of this variable is to reflect how the sum of EMS resources assigned to a mission the current hour effects response time in individual missions. Differences in the sum of EMS resources in relation to the sum of missions affects response time.

### Statistical methods

This study employed SAS Enterprise Guide 8.2 for data standardisation and manipulation, ensuring rigorous statistical procedures. Furthermore, SAS Viya 3.05 and Visual Analytics 8.5.2 facilitated comprehensive data analysis. The software allows the user to control the settings and tune the machine learning models. The specific code utilised for these analyses is outlined in Appendix D for easy reference and reproducibility.

#### Linear regression

To explore the relationships between the variables and response time, we utilised a linear regression model with 20 bins and a depth of 5 percentiles with a tolerance of 1e–10 [53, 54]. The objective of this analysis was to validate the impact of various factors on the response time (Appendix C.2).

#### Machine learning models

To construct a reliable prediction model for response time, the dataset underwent partitioning, with 60% allocated for training data, 30% allocated for validation data and 10% as test data. Each model where autotuned by the software to optimise the performance. Various models, including random forest, gradient boosting, neural network, and linear regression, have been investigated and juxtaposed for comparative analysis [52, 53, 61, 62]. Partial Dependence-plots (PDP) where used to interpret the models and gain understanding of the features of interest. The PDP illustrate the marginal effect of one or two features on the predicted outcome, averaged over the range of values of other features. They help to understand how a single feature influences predictions while holding all other variables constant [63–65]. The gradient boosting model was used to investigate the overall data and data stratified by priority level with the settings reported in Table 3.

**Table 3:** Gradient boosting settings

Gradient Boosting model settings				
	Overall	Priority 1	Priority 2	Priority 3
Auto-stop method	Stagnation	Stagnation	Stagnation	Stagnation
Auto-stop iterations	4	4	4	4
Number of trees	50	50	50	50
Learning rate	0.23	0.23	0.23	0.23
Subsample rate	0.1	1	1	1
Lasso	6.11111111	1.11111111	1.11111111	0
Ridge	2.22222222	6	1	2.22222222

### Ethical considerations

All procedures were performed in compliance with relevant laws and institutional guidelines and were approved by the Swedish Ethical Review Authority 2022-09-13 Dnr 2022-03701-01. The study was conducted in accordance with the Declaration of Helsinki and good clinical practice [66, 67].

## Results

**Fig. 2:**
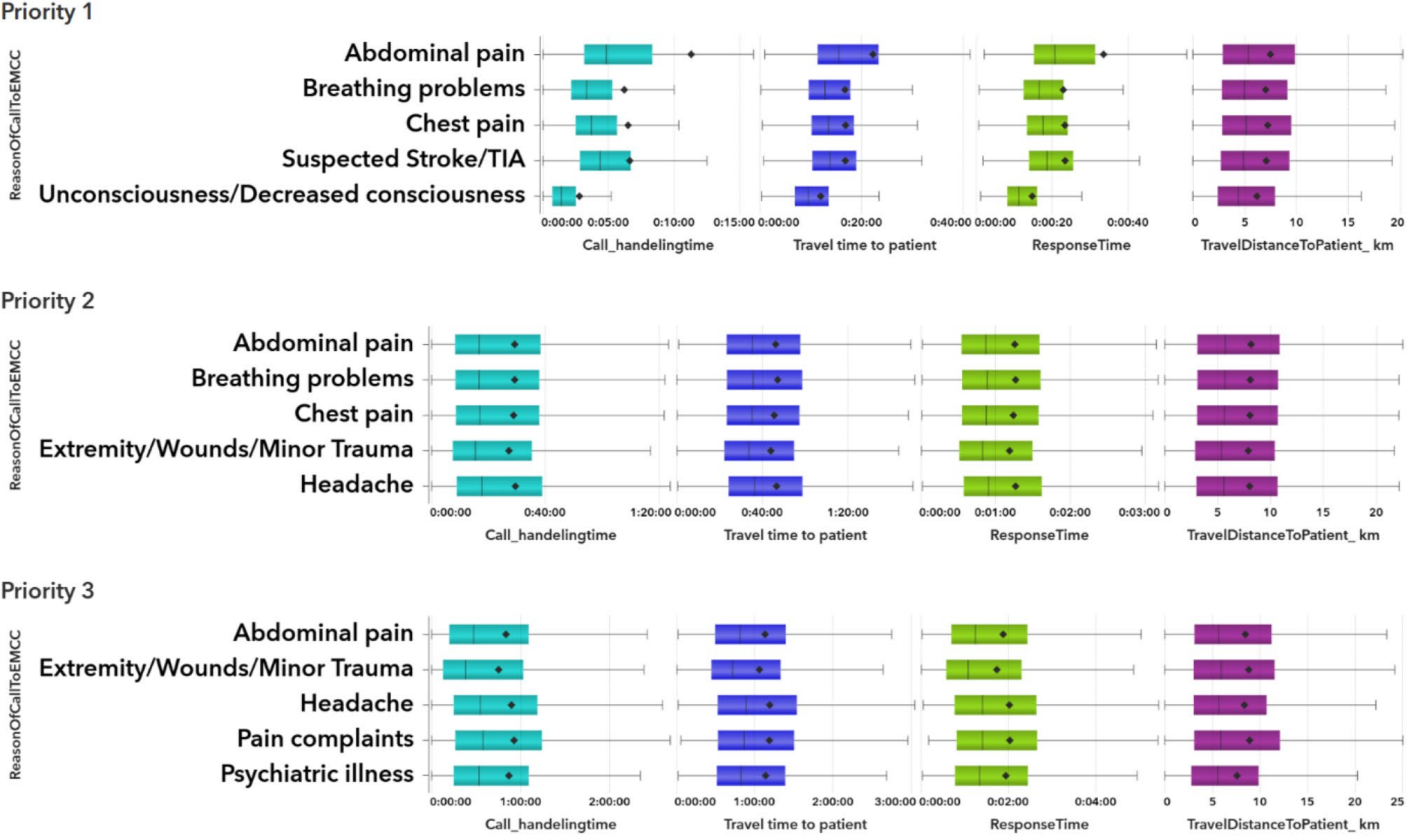
Boxplots of call handling, travel, and response times for the 5 most common call types by EMCC priority

The main result showed that complex factors influencing EMS response times. Call handling times impact on overall response time varied by both call reason and urgency level. Travel time and distance likewise differed across call types and priorities. Calls for certain issues (e.g., breathing problems, unconsciousness) consistently had shorter response and travel times, while others (e.g., abdominal pain) had longer handling times, suggesting potential areas for operational improvement (Fig. 2).

### Feature importance

**Fig. 3:**
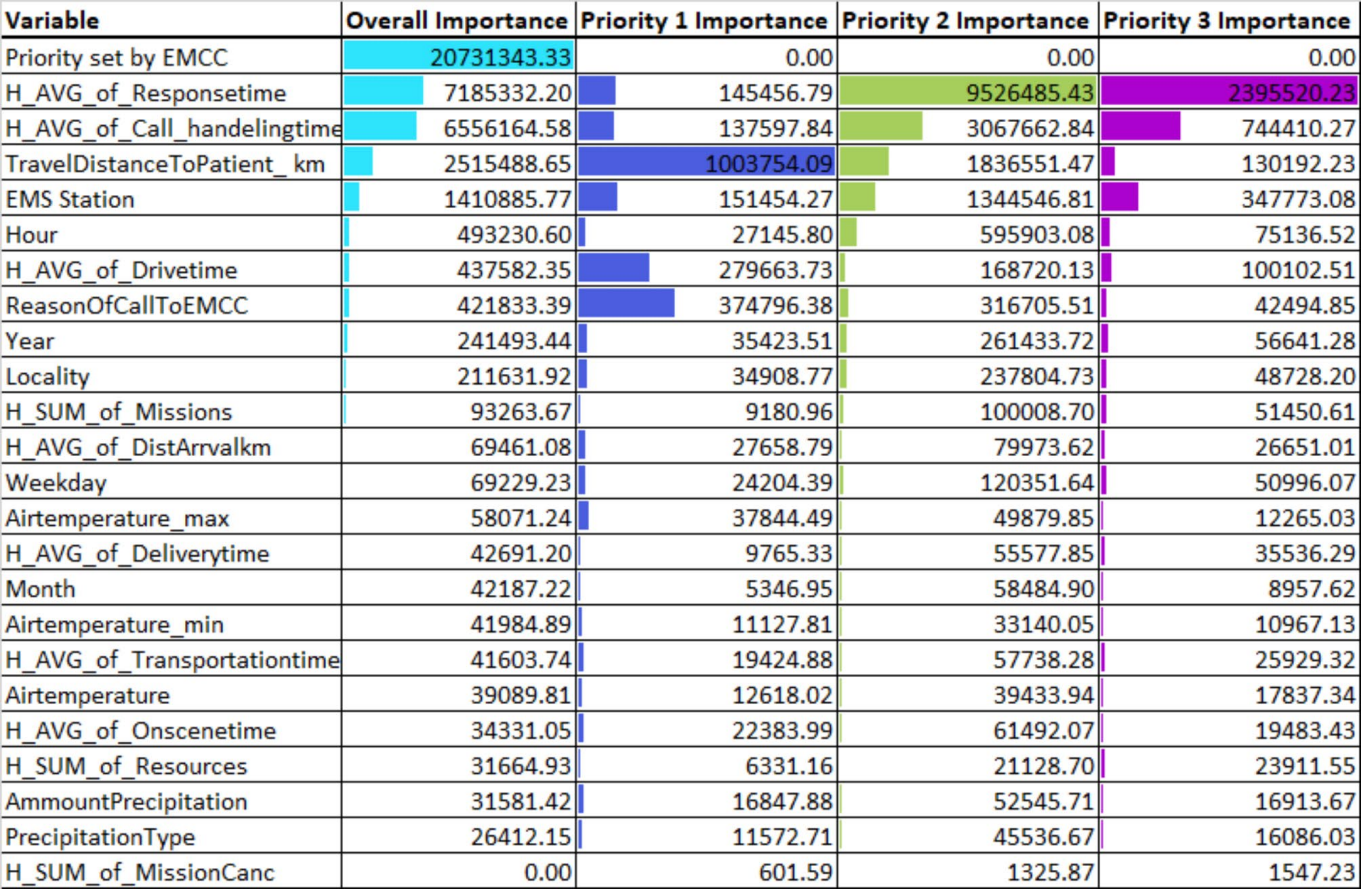
Feature importance in overall data and by urgency level; color-coding to show highest impact

Feature importance varied, with EMCC priority level emerging as the most influential in the overall model (Fig. 3).

Interestingly, some features (e.g., the “hourly sum of missions”) showed opposing effects across different priorities. The result showed an increased workload shortens the Priority 1 response times (perhaps due to resources already are on route to other lower prioritized patients and are redirected to higher prioritized patients), by the PDP used to interpret the model. However, the response time was lengthened for Priority 2 (Fig. 3).

**Fig. 4:**
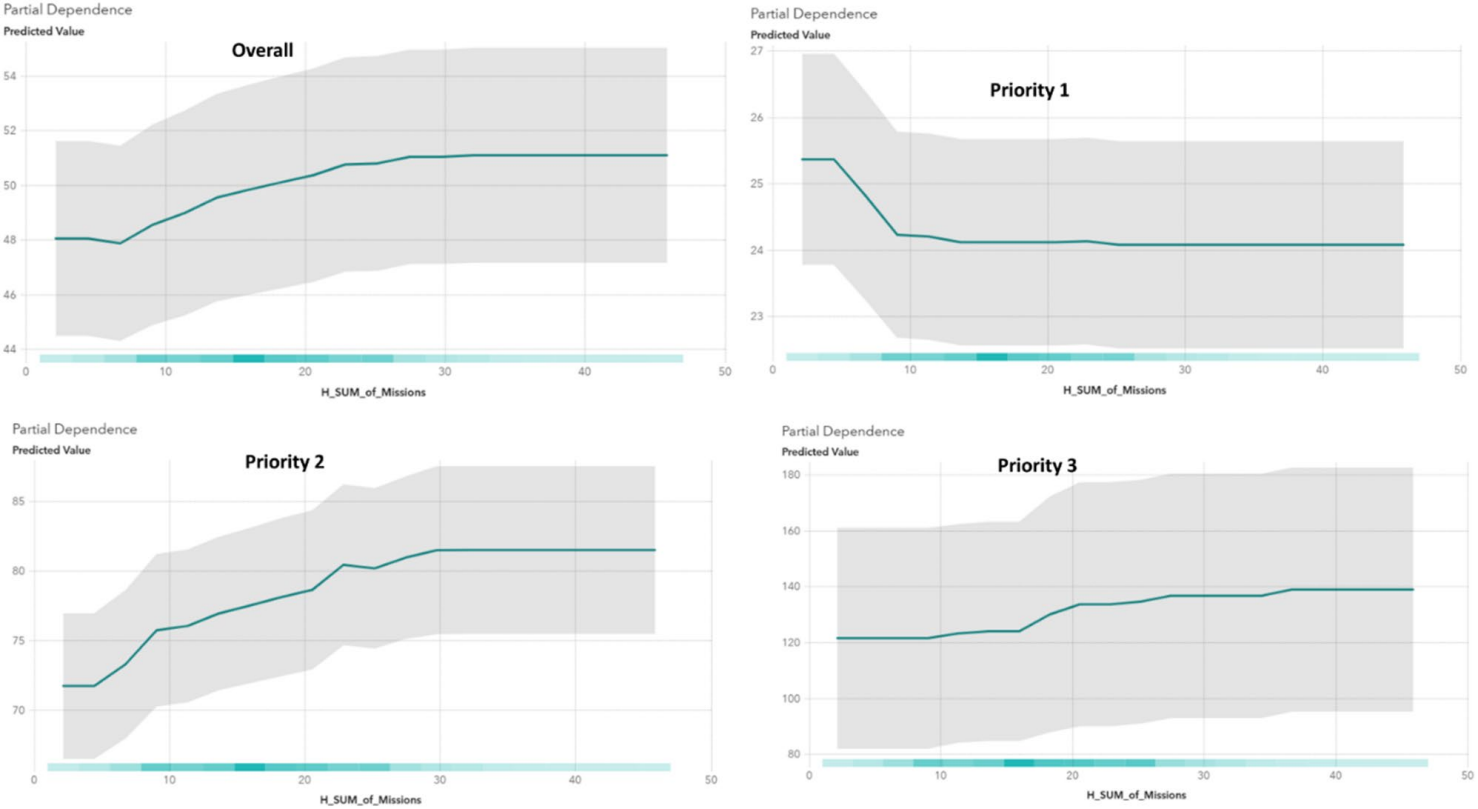
PDP of the hourly total sum of missions at different priority levels compared to predicted response time

Temperature and precipitation also played roles. Precipitation was generally associated with increased response times, and extreme cold (< − 8 °C) affected Priority 1 calls more than moderate temperatures. Conversely, higher temperatures (> 18 °C) tended to affect lower-priority calls. Local geography influenced response time variations, particularly in how urgency levels interacted with different areas. PDPs revealed that high-priority calls in central Stockholm had consistently lower response times compared to suburban or rural areas. However, for lower-priority calls, this trend reversed in some cases, with central areas experiencing longer response times than expected. This suggests that resource saturation at peak times may lead to a trade-off where high-priority cases are prioritized at the expense of lower-priority calls. Additionally, in some lower-density areas, ambulances may be stationed farther apart, leading to naturally longer response times, which disproportionately affect lower-priority dispatches (Appendix A.34).

The results show that different features have different effect on response time which confirms the relationships in the DAG (Fig. 1). Multiple features interact to influence EMS response time. PDPs show that while urgency level is the strongest predictor of response time, its effect varies depending on geographic location and real-time system strain. For instance, in areas with high EMS demand, the travel time component increases significantly for lower-priority calls, whereas high-priority calls maintain stable response times. This effect suggests that EMS resource allocation strategies should not only prioritize high-urgency cases but also account for cumulative system strain to prevent excessive delays in less urgent cases.

One example is determinants driving calls to EMCC, including call handling duration, travel time to the patient, and the urgency level of the situation, which collectively influence response time (Fig. 2). Among these factors, both the duration of call handling and the travel time to the patient emerge as influencers alongside the priority level set by EMCC, introducing variability into the overall response time.

A PDP revealed that specific reasons for contacting the EMCC, such as breathing problems and loss of consciousness, are associated with shorter call handling and travel times. This finding underscores that these variables not only directly impact response time but also exert indirect effects by influencing other factors. For example, within priority level 1, variations in response time are observed across different reasons for contacting the EMCC (Appendix A.18). The predicted response time differs from about 15 min for Unconsciousness and Cardiac arrest compared to over 30 min for Abdominal pain and Extremity/Wound/Minor trauma, all in the same priority level 1 (Fig. 4).

**Fig. 5:**
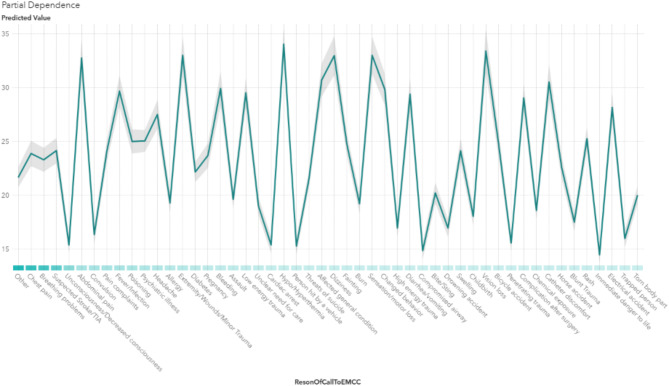
PDP of the Reason of Call to EMCC compared to predicted response time in the priority 1 level. Calls for abdominal pain and minor trauma experience longer response times despite being priority 1 while unconsciousness and cardiac arrest exhibit the shortest response times

By shedding light on these relationships, this study elucidates how various factors interact to shape emergency response times, offering insights crucial for optimising EMS on the basis of response time.

The EMS response time is subject to the influence of various factors beyond immediate call handling and travel logistics. Among these factors, the timing of incidents throughout the day is a significant determinant. The analysis revealed discernible fluctuations in response times across different hours, with particularly noteworthy impacts evident during periods of varying urgency levels, especially compared with instances of the highest urgency (Appendix A.22).

Furthermore, the geographical location of incidents plays a pivotal role in shaping response time dynamics across different urgency levels. Notably, the central region of Stockholm has markedly shorter response times during high-urgency scenarios. Conversely, during lower-urgency scenarios, this same area experiences prolonged response times in comparison to other geographical localities (Appendix A.34).

### Distribution of response time

**Fig. 6:**
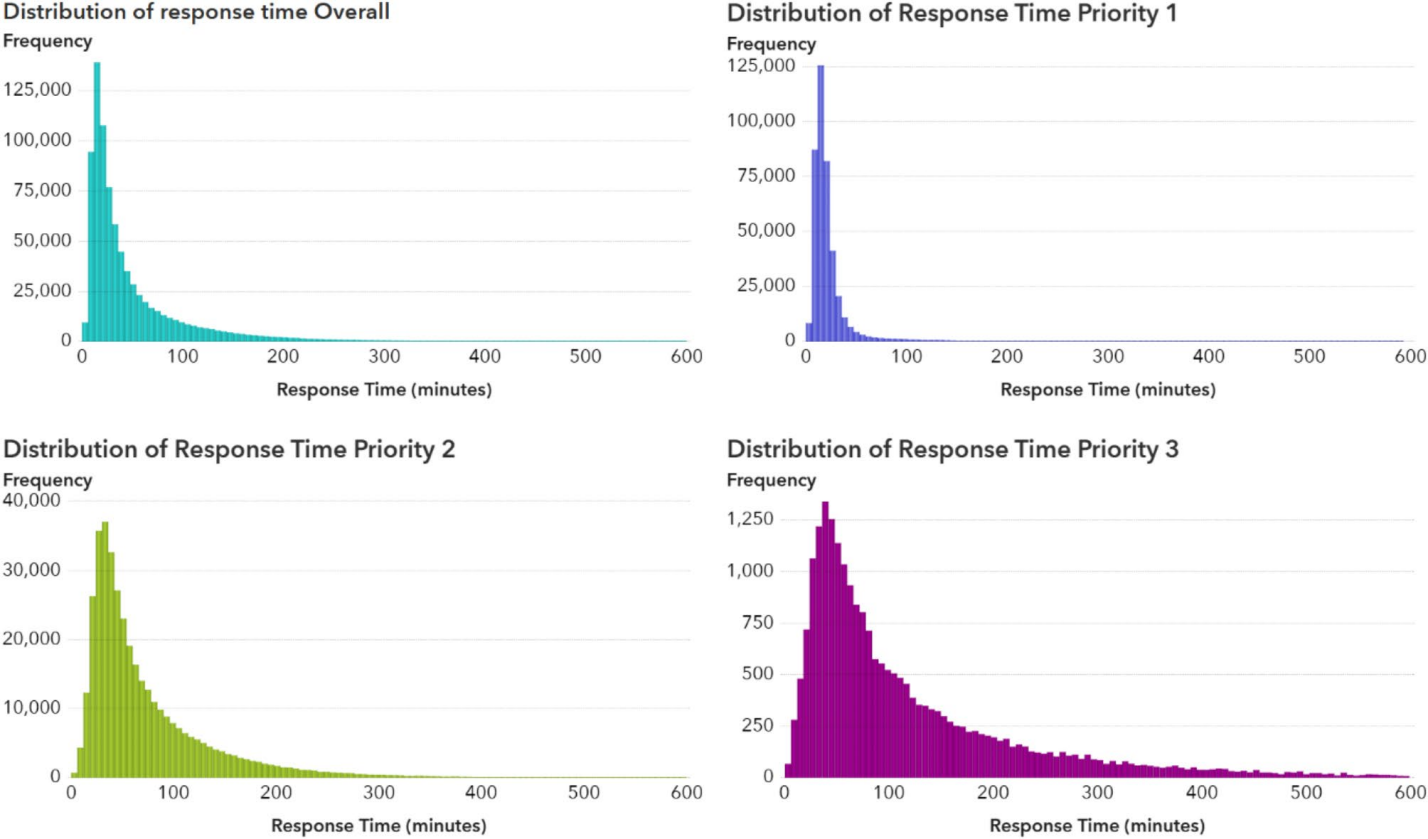
The histogram shows distribution of response time in different priority levels set by EMCC

Response time distribution was heavily skewed for priority levels 2 and 3, featuring a “long tail” of extended response times (Fig. 5). This skew can reduce ML model generalizability by overemphasizing outliers. Because the aim was to deepen the understanding of the complex factors influencing EMS response times, including these long-tail cases, rather than to build a perfect predictive model, this trade-off was considered acceptable (see Fig. 6).

### Model performance

Overall, the gradient boosting model produced an ASE of 1757.70 min in the test data. Performance improved when stratified by priority level, with Priority 1 yielding the best prediction accuracy (ASE = 545.99) and Priority 3 the lowest (ASE = 6752.75), reflecting that Priority 3 data were more skewed (Table 4).

**Table 4:**
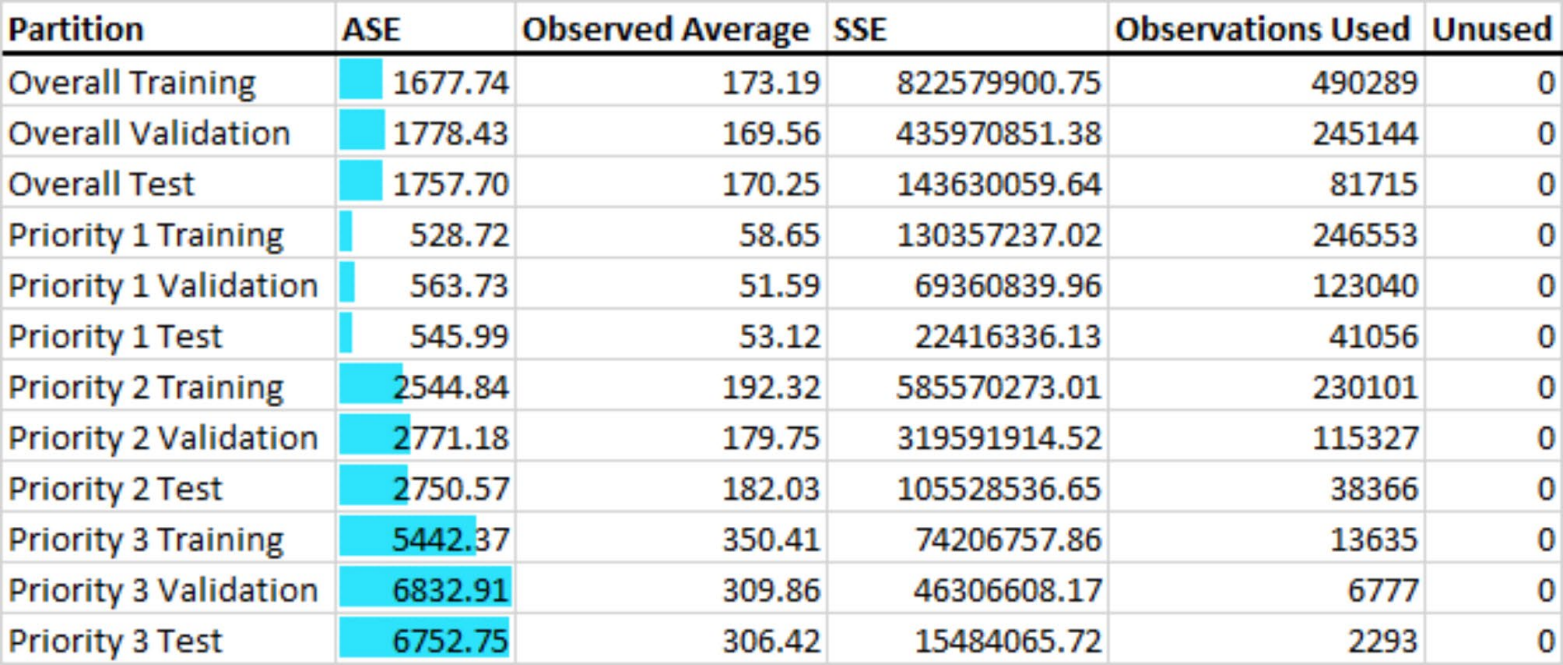
Gradient boosting machine learning models, overall and stratified by priority level set by EMCC

Linear regression indicated significant associations between a vast number of variables and response time (average squared error [ASE] = 2127.1291 in the test data; R² = 0.3706). This R² value, while not extremely high, aligns with the DAG’s suggestion that additional unmeasured factors also play a role. Of the tested ML models, gradient boosting demonstrated the best fit, though differences among models were slight. Table 5 (below) compares model performance (Appendix B.2).

**Table 5:** Model comparison where gradient boosting was selected as the best fit

Selected	Number Of Observations	Percentile	Predicted Average	ASE	Observed Average	SSE	Model
Yes	490,289	5	167.80	1690.09	172.90	828634299.32	Training: Gradient boosting
Yes	245,144	5	168.22	1789.91	169.40	438785173.01	Validation: Gradient boosting
Yes	81,715	5	167.29	1769.54	170.06	144598368.52	Test: Gradient boosting
No	490,289	5	168.66	1307.06	194.87	640835098.42	Training: Forest
No	245,144	5	166.32	1796.18	168.69	440322314.81	Validation: Forest
No	81,715	5	165.52	1786.66	169.21	145997039.91	Test: Forest
No	222,482	5	167.41	1788.88	169.01	397994152.56	Training: Neural network
No	110,779	5	168.11	1861.60	166.61	206226675.73	Validation: Neural network
No	37,213	5	168.51	1832.01	166.76	68174404.09	Test: Neural network
No	222,482	5	129.17	2116.45	158.05	470871881.72	Training: Linear regression
No	110,787	5	129.46	2138.34	157.61	236900262.60	Validation: Linear regression
No	37,214	5	129.65	2127.13	157.27	79158981.29	Test: Linear regression

## Discussion

### Key results

This study aimed deepen the understanding of the complex factors influencing EMS response times. Confirming previous research [9, 10], call handling and travel time are vital, but new detail emerged on how different call types within the same priority can yield substantial variations. Calls for unconsciousness produced shorter handling times, while abdominal pain or urinary issues were lengthier all in the highest priority level.

Environmental factors like precipitation also significantly influenced response times, aligning with prior findings [33, 35, 40, 42]. The Machine Learning model allowed for better recognition of these “uncontrollable” contributors to response time.

### Implications for EMS management

By leveraging machine learning for predictive modeling, EMS agencies can optimize resource allocation and improve response times. To operationalize these insights, EMS should develop a dynamic resource allocation system by implementing real-time monitoring dashboards that integrate machine-learning-driven predictive alerts for demand surges based on call type, weather conditions, and geographic trends. Shifting from fixed stationing to a fluid deployment model, where ambulances dynamically reposition based on real-time forecasts, would enhance efficiency.

Emergency call prioritization can be improved by deploying AI-assisted triage systems in Emergency Medical Communication Centers (EMCCs) to refine priority classification and reduce misallocations of high-priority responses. Training dispatchers to use machine learning-backed decision support tools would ensure that emergency calls are matched with appropriate response levels based on historical trends and real-time data. Integrating traffic and weather data for smarter routing is also essential. Automated rerouting protocols should be established to adjust dispatch recommendations based on live traffic feeds and adverse weather forecasts, while partnerships with municipal traffic authorities could facilitate the creation of EMS priority lanes or dynamic traffic signal preemption for ambulances in high-demand areas.

Addressing equity in response times requires a geospatial analysis to identify high-risk zones with prolonged response times and adjust resource distribution accordingly. Allocating additional EMS units or mobile response teams in underserved areas during peak demand periods or severe weather conditions would ensure a more balanced deployment of resources. Furthermore, improving data-driven decision-making through training and feedback mechanisms is crucial. Specialized training programs should be developed to ensure EMS personnel and dispatchers can interpret machine learning-based insights and adjust operational decisions accordingly. Establishing a feedback mechanism where EMS field teams report real-world response discrepancies would enable continuous model refinement for improved accuracy.

Strengthening collaboration between EMS and policymakers is necessary to translate these insights into structural improvements. Engaging local governments in policy discussions on urban planning would help ensure that new infrastructure investments align with machine learning-based response efficiency insights. Additionally, advocating for funding support to scale real-time machine learning deployment, particularly in regions with historically slow response times, could facilitate the implementation of these advanced strategies.

By systematically adopting these measures, EMS organizations can transition from reactive response models to proactive, data-driven emergency care strategies. These changes will enhance response efficiency, improve equity in service delivery, and ultimately lead to better patient outcomes.

### Methodological considerations

This study employed a robust methodological framework combining advanced machine learning techniques and traditional statistical approaches to analyze a complex dataset of over one million EMS missions. A key strength of the methodology was the use of feature engineering and the construction of a directed acyclic graph (DAG) to elucidate relationships among variables. This approach ensured a comprehensive understanding of the factors influencing response times.

The dataset’s partitioning into training, validation, and test sets allowed for rigorous model evaluation, and the use of Gradient Boosting models provided nuanced insights into feature importance. However, the exclusion of extreme outliers, such as response times exceeding 10 h, may limit the generalizability of the findings to atypical cases.

Ethical considerations were integral to the study’s design, with adherence to the STROBE checklist ensuring clarity and rigor. Limitations include the reliance on aggregate metrics, which may obscure finer temporal trends. The absence of traffic data and other operational constraints also highlights areas for future research.

### Challenges and considerations for real-time implementation

The real-time implementation ML predictions in EMS operations presents both significant opportunities and notable challenges. While the potential of ML to dynamically optimize resource allocation is clear, translating these insights into live operations requires addressing several practical considerations.

A key requirement for real-time deployment is robust computational infrastructure. Systems capable of processing large datasets and generating instantaneous predictions are essential. This may necessitate investments in advanced technologies such as cloud-based solutions or edge computing to meet the demanding computational requirements.

Seamless integration of diverse data sources is another critical factor for effective real-time predictions. Combining weather updates, traffic conditions, and real-time EMS resource availability requires interoperable systems and standardized data protocols to ensure smooth operation and data flow.

Operational constraints within EMS systems must also be carefully considered. Regulatory and logistical requirements, including variations in response protocols, resource limitations, and the need for rapid decision-making during high-stakes scenarios, necessitate models that are both adaptable and compliant with these constraints.

The adoption of real-time ML systems further depends on adequate training for EMS personnel and dispatchers. These individuals must be equipped to interpret ML predictions and incorporate them into their workflows. User-friendly interfaces and decision support tools can significantly enhance the usability and acceptance of these systems.

Lastly, continuous monitoring and feedback mechanisms are essential for maintaining the accuracy and reliability of real-time ML systems. Establishing feedback loops that refine models based on operational outcomes will ensure their long-term utility and trustworthiness.

By addressing these challenges, the implementation of real-time ML systems can transition from theoretical potential to practical reality, enhancing the efficiency and responsiveness of EMS operations.

### Limitations and directions for future research

The study identifies several limitations, including the exclusion of traffic data and operational constraints, but further elaboration is necessary to contextualize their impact and provide direction for future research. Traffic conditions are a critical factor influencing EMS response times, especially in urban environments. The absence of traffic data in the models could result in underestimating delays during periods of peak congestion. Future studies should aim to incorporate real-time traffic feeds or historical congestion patterns to enhance model accuracy.

Operational variability also poses challenges to the generalizability of the findings. Differences in EMS standard operating procedures, such as dispatch protocols and resource allocation strategies, could influence outcomes. Expanding the analysis to include regional or organizational variations would provide a more comprehensive understanding of these effects and their implications.

While the study includes weather data, its focus is limited to Stockholm. Regional weather patterns, such as extreme heat in arid climates or heavy snowfall in colder regions, may have distinct impacts on response times. Exploring these variations could improve the robustness and applicability of the models to diverse geographic settings.

The use of aggregated features, such as hourly averages, may obscure finer temporal trends. To address this, future research should investigate shorter time intervals to better capture fluctuations in EMS demand and response dynamics. This approach would allow for a more detailed understanding of system behaviour under varying conditions.

Finally, while the study highlights the potential for real-time machine learning applications, it does not address the technical and logistical challenges of implementing such systems in live EMS operations. Investigating successful case studies of real-time ML integration could provide valuable insights and practical solutions for overcoming these barriers.

By addressing these limitations, future research can build on the current findings to further advance the effectiveness and reliability of EMS resource planning and response strategies.

### Generalizability

The machine learning model developed in this study demonstrates a high level of adaptability for other EMS systems, as it is built on widely applicable factors such as call handling time, travel distance, urgency levels, weather conditions, and geographic variability. These determinants influence EMS response times universally, making the model broadly relevant to emergency medical services beyond Stockholm. The use of Gradient Boosting and feature engineering techniques ensures that the model captures complex interactions between these variables, allowing for meaningful insights into response time dynamics. Given that many urban EMS systems operate with structured dispatch protocols and centralized emergency communication centres, the core structure of the model can be readily applied in similar settings.

However, certain modifications would be necessary when adapting the model to EMS systems with different operational structures. Some EMS systems categorize priority levels differently or use alternative dispatch methods, which may require adjustments to the model’s classification framework. Additionally, EMS systems that utilize a tiered response structure, where basic life support and advanced life support teams are dispatched separately, may need to introduce additional features to capture these nuances in resource allocation.

Geographic and traffic-related variations also present challenges when applying the model across different regions. While the Stockholm EMS system operates in a metropolitan setting with both fixed and fluid deployment strategies, the model may require recalibration when applied to rural areas where response times are naturally longer due to increased distances between incidents and available EMS units. Furthermore, the absence of real-time traffic data in the Stockholm dataset means that the model may need to be supplemented with live traffic inputs in cities where congestion significantly impacts response times.

## Conclusion

This study provides a comprehensive analysis of the multifactorial determinants of EMS response times, demonstrating the significant influence of variables such as urgency levels, weather conditions, and resource availability. By leveraging advanced machine learning techniques, particularly Gradient Boosting models, the research offers a nuanced understanding of how these factors interact and provides a robust framework for predictive analytics in EMS operations.

Key findings underscore the importance of incorporating real-time data into dynamic resource allocation strategies. For high-priority calls, optimizing dispatch processes and leveraging predictive models can reduce response times and enhance patient outcomes. For lower-priority calls, addressing systemic inefficiencies, such as resource imbalances and weather-related delays, can improve equity and service delivery.

The implications for EMS management are substantial. Transitioning from static to adaptive deployment models can enable agencies to better respond to fluctuations in demand and external conditions. Integrating machine learning predictions into live operations offers a path to more efficient, equitable, and sustainable emergency care systems.

Future research should address limitations such as the exclusion of traffic data and operational constraints and explore the generalizability of these models to rural or smaller urban settings. Policymakers and EMS administrators are encouraged to consider the study’s findings to inform decisions on funding, resource allocation, and deployment strategies.

By bridging the gap between academic research and operational practice, this study lays the groundwork for a data-driven evolution in EMS planning, aiming to optimize response times and improve outcomes for all patients, regardless of urgency level or location.

## Electronic supplementary material

Below is the link to the electronic supplementary material.

Supplementary Material 1

## Data Availability

Due to the sensitive nature of the used data in this study, where data is confidential due to the Swedish Public Access to Information and Secrecy Act. It is possible to request the used data from Region Stockholm and their Centre of Health data.
