# Supplementary material for: Understanding EMS response times: a machine learning-based analysis
Source: BMC Med Inform Decis Mak. 2025 Mar 24;25:143. doi: 10.1186/s12911-025-02975-z (PMC11934472; doi:10.1186/s12911-025-02975-z)
Supplement: Supplementary file 1 — Supplementary Material 1 [file 12911_2025_2975_MOESM1_ESM.docx]

# Appendix

## Partial Dependence

### Priority set by EMCC


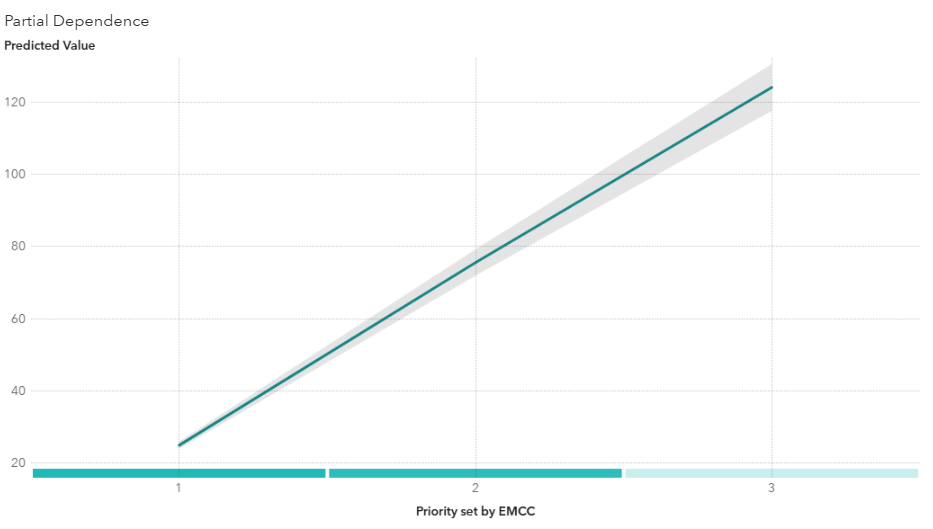


### H_AVG_of_Responsetime


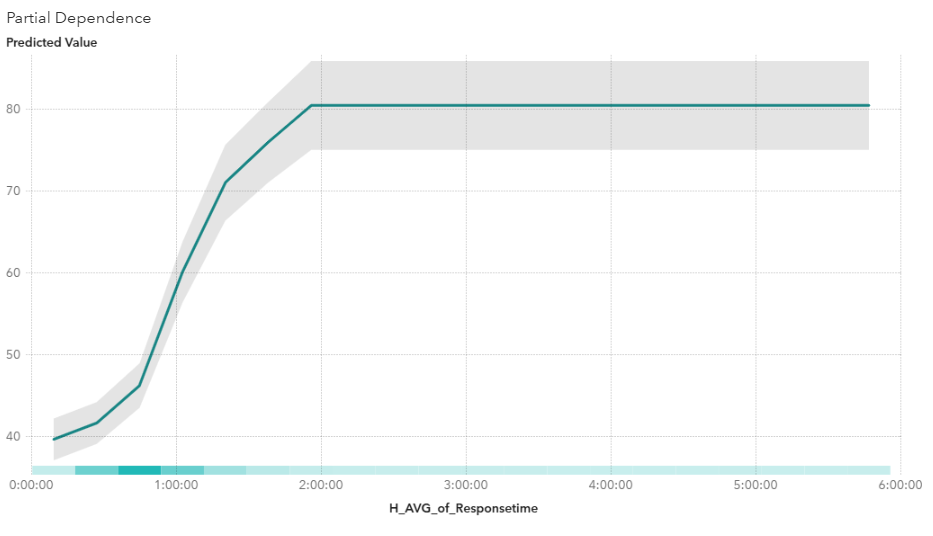


### H_AVG_of_Responsetime priority level 1


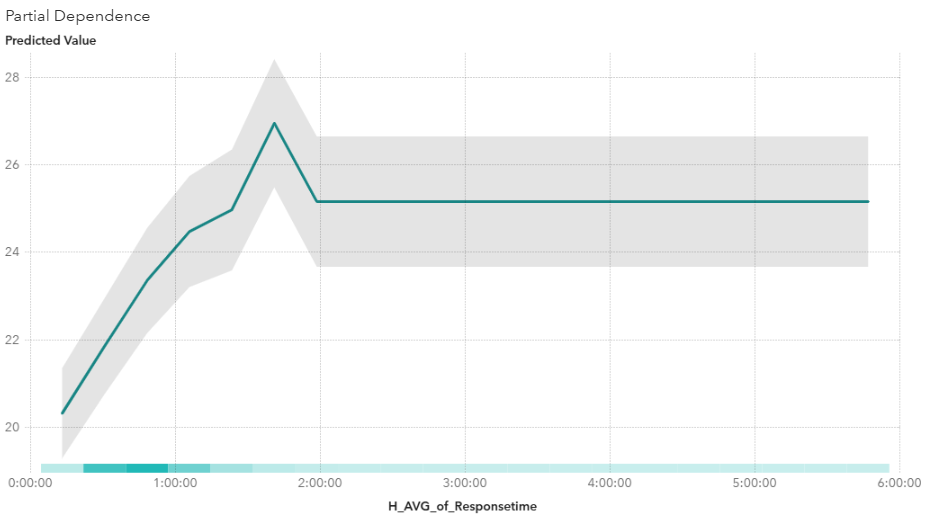


### H_AVG_of_Responsetime priority level 2


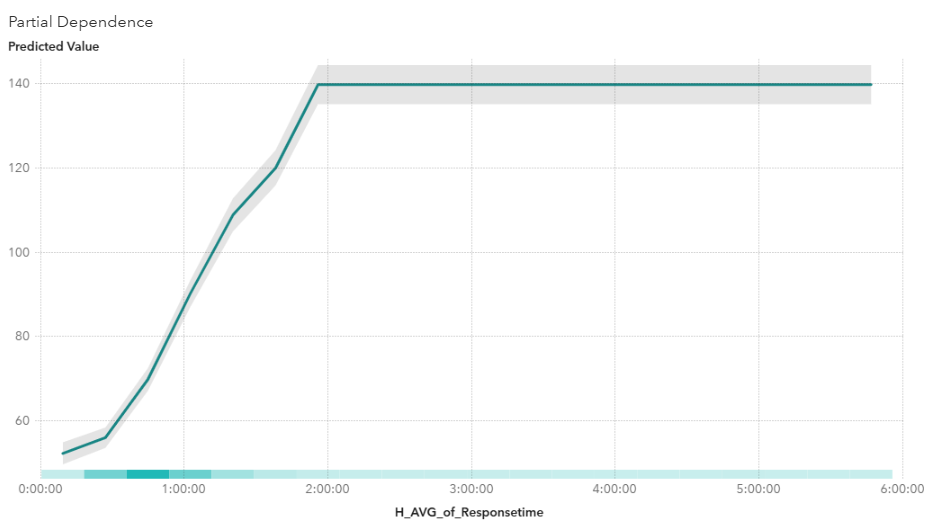


### H_AVG_of_Responsetime priority level 3


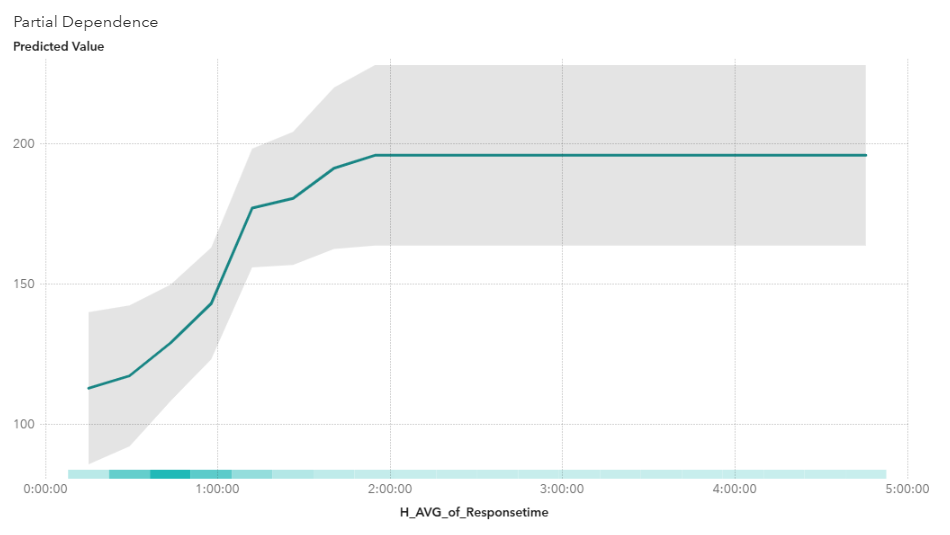


### H_AVG_of_Call_handelingtime


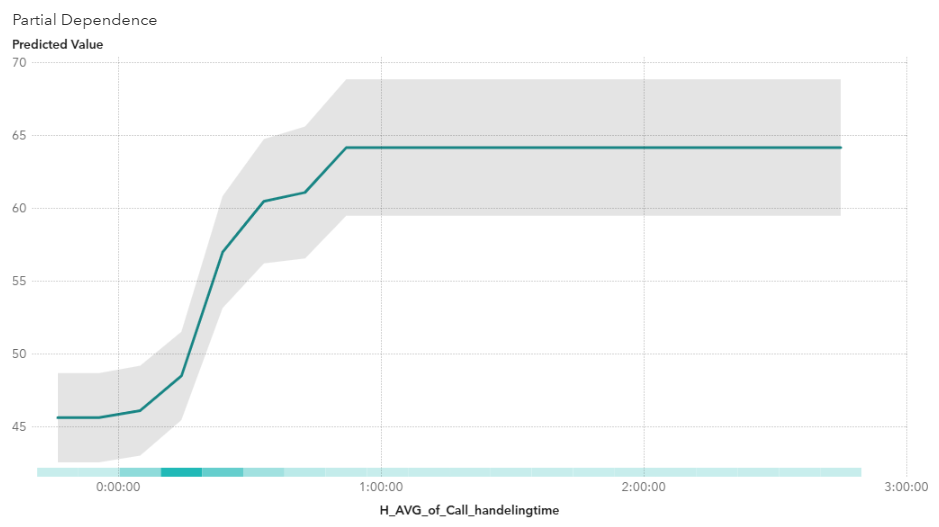


### H_AVG_of_Call_handelingtime priority level 1


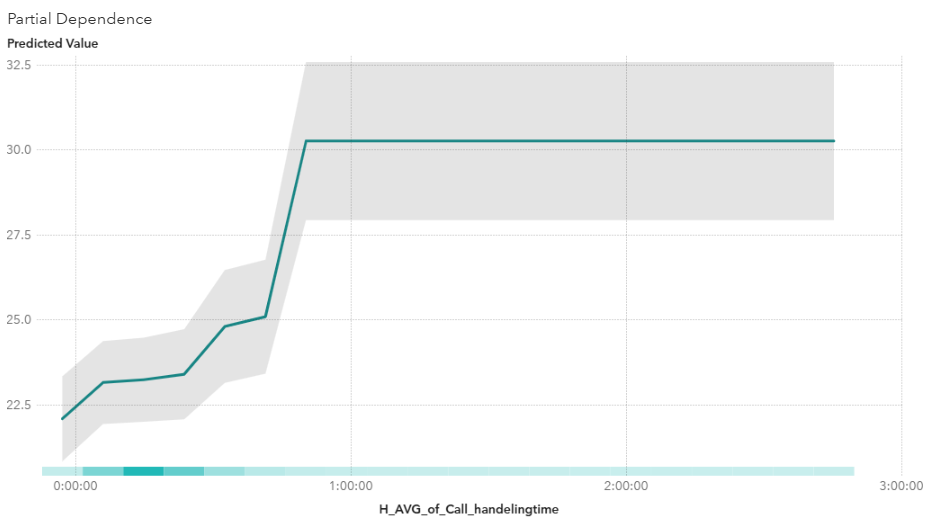


### H_AVG_of_Call_handelingtime priority level 2


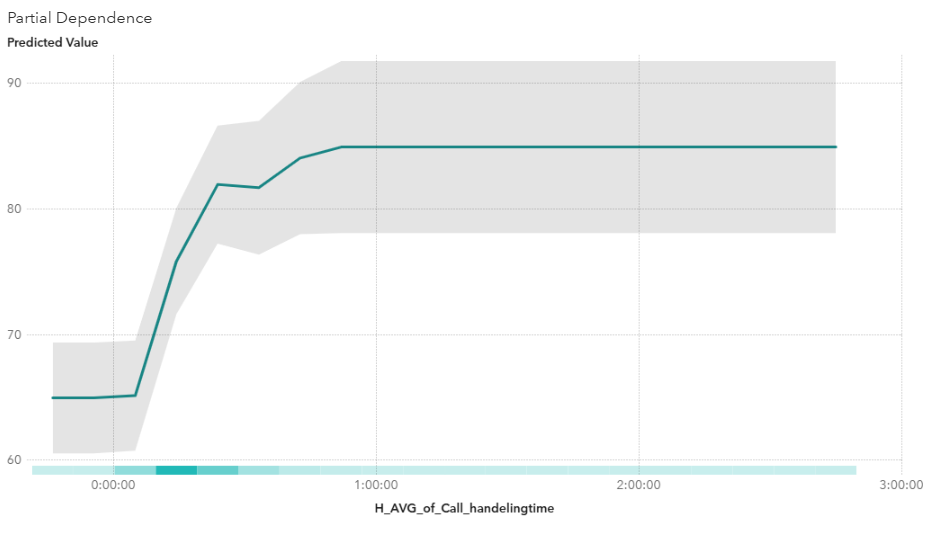


### H_AVG_of_Call_handelingtime priority level 3


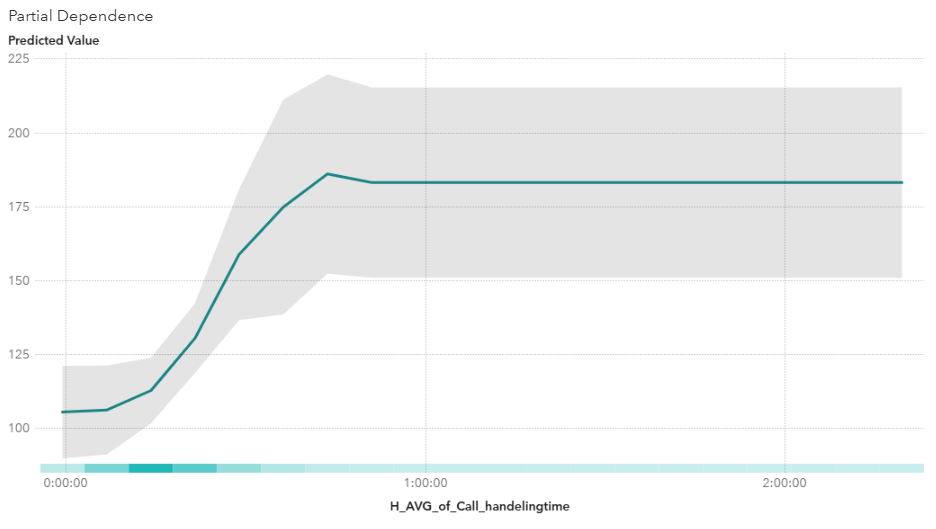


### TravelDistanceToPatient_km


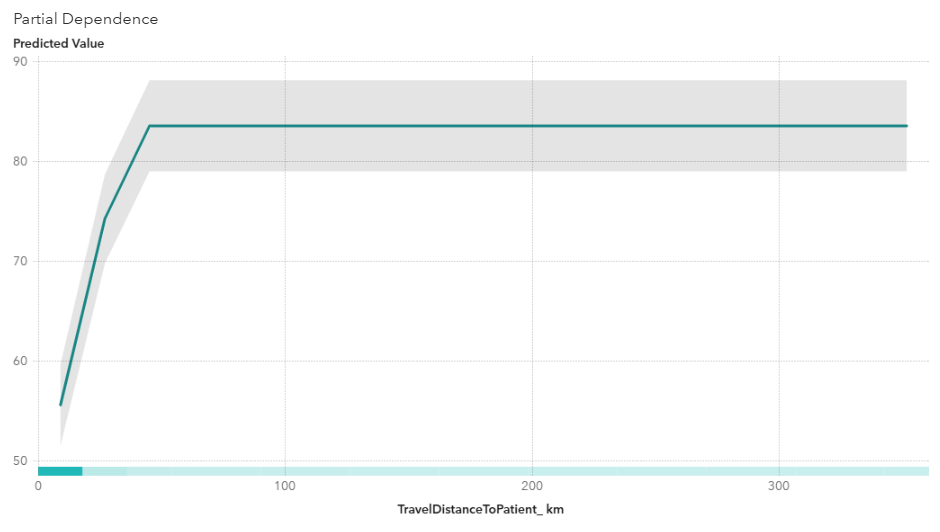


### TravelDistanceToPatient_km priority level 1


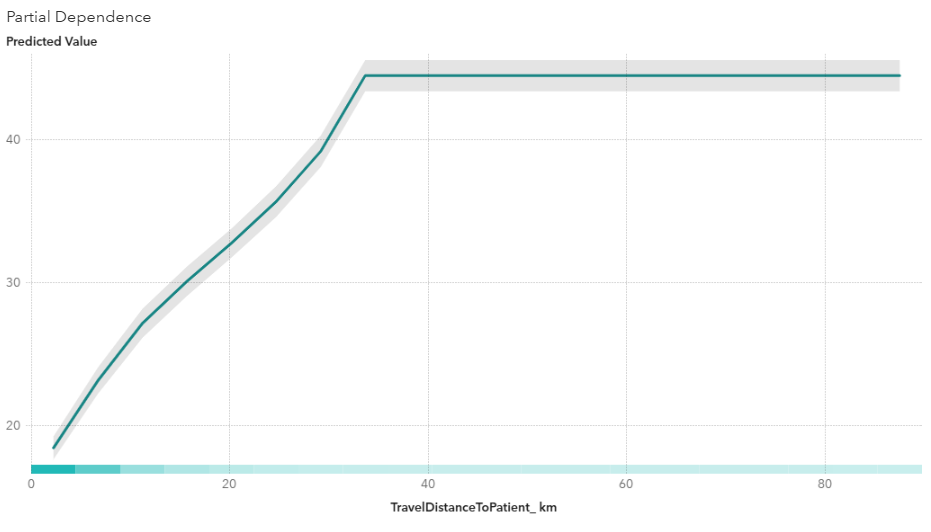


### TravelDistanceToPatient_km priority level 2


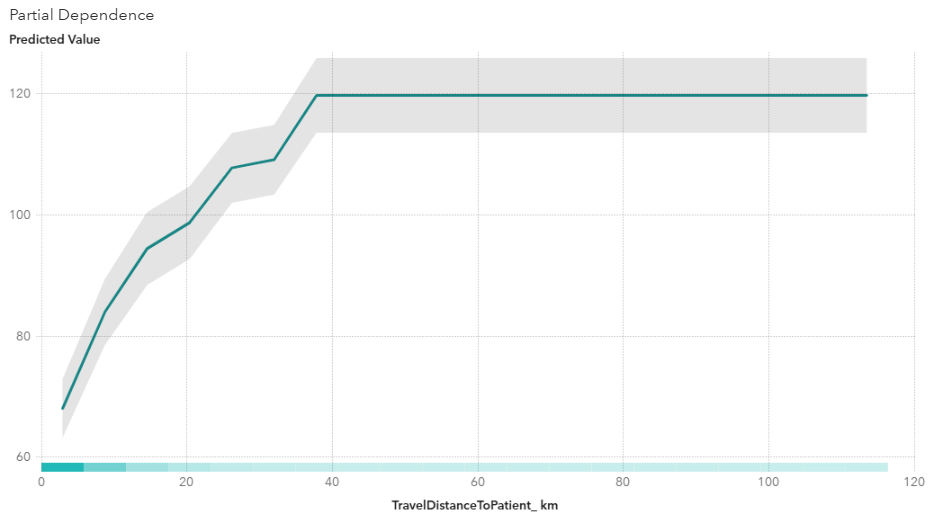


### TravelDistanceToPatient_km priority level 3


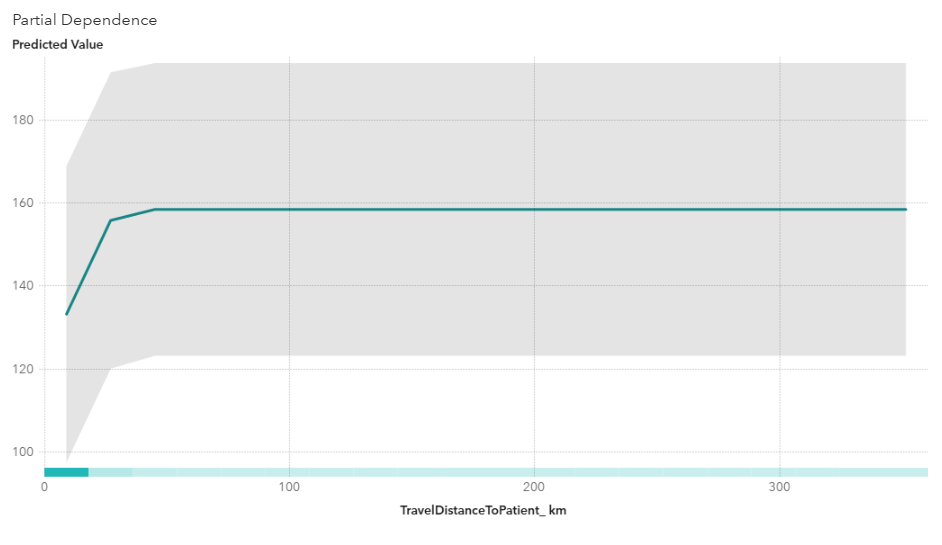


### Station


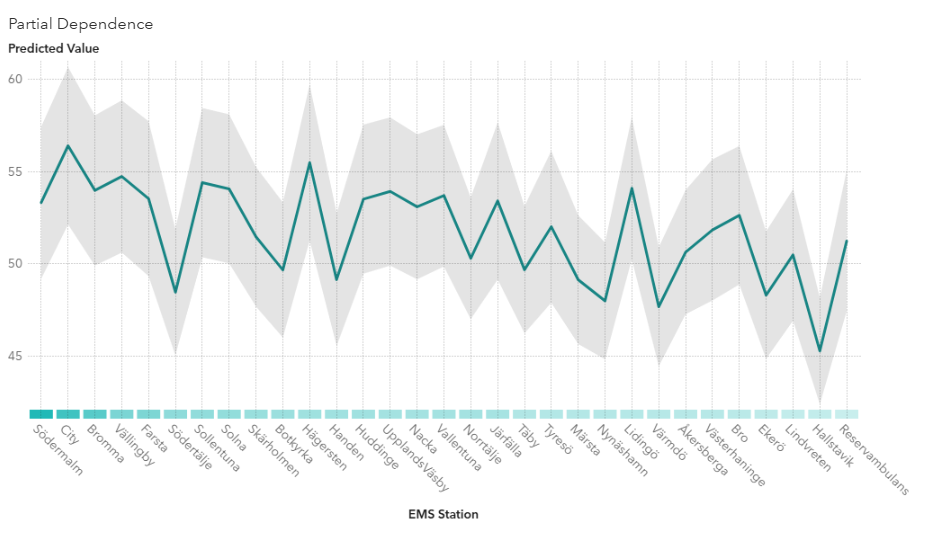


### Station priority level 1


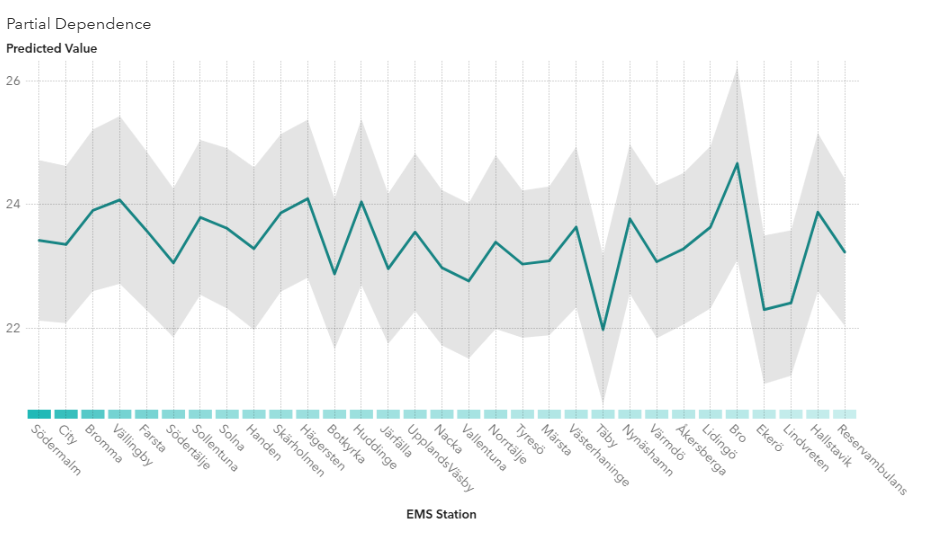


### Station priority level 2


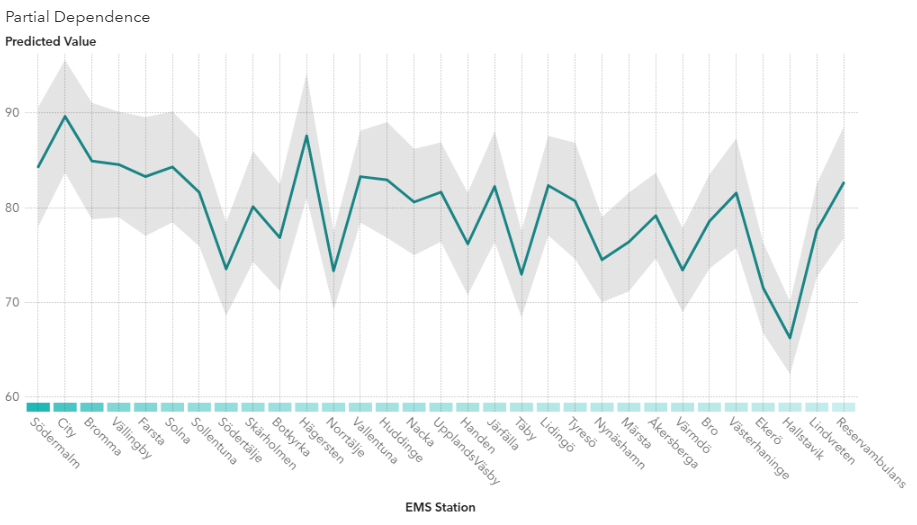


### Station priority level 3


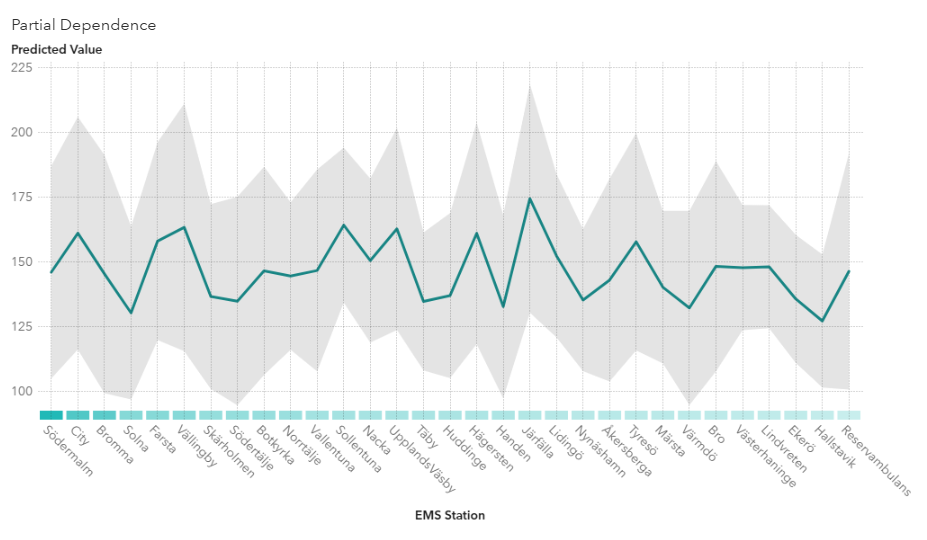


### Reason of Call to EMCC


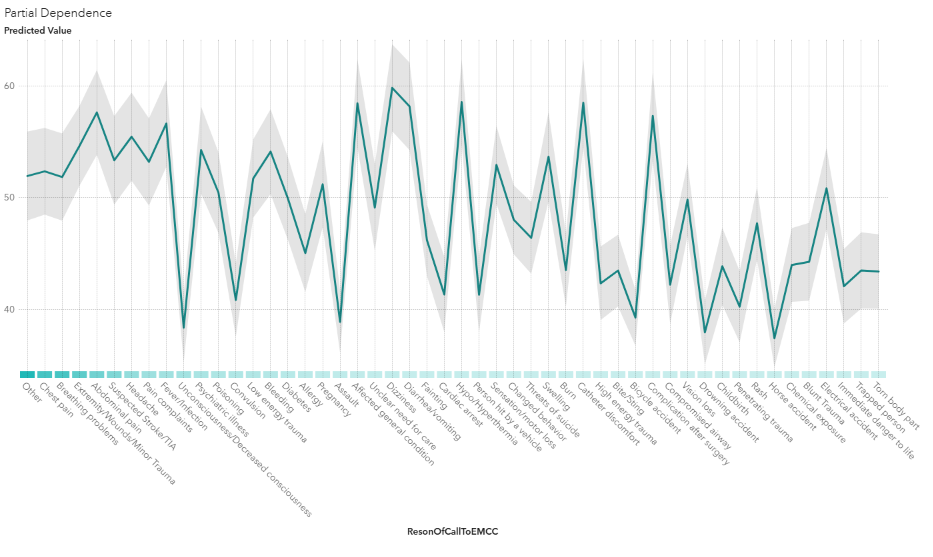


### Reason of Call to EMCC priority level 1


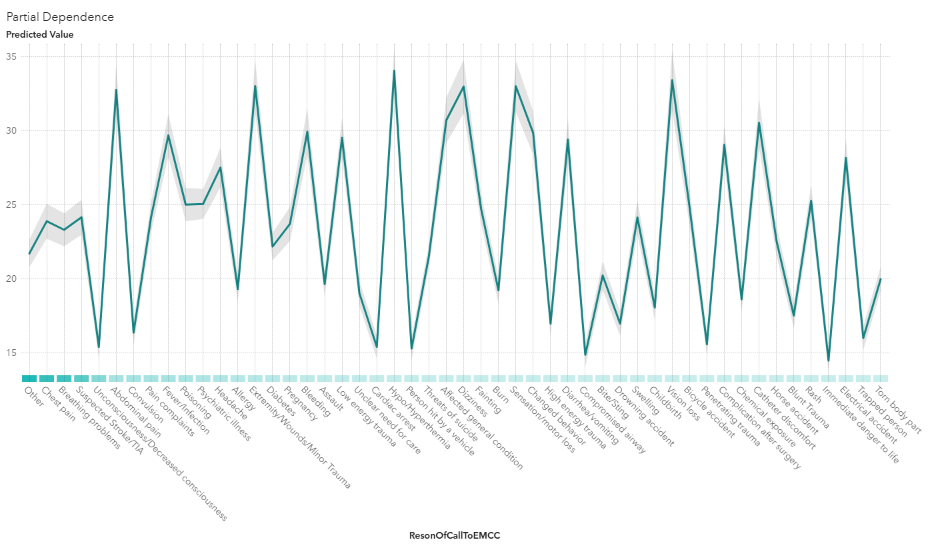


### Reason of Call to EMCC priority level 2


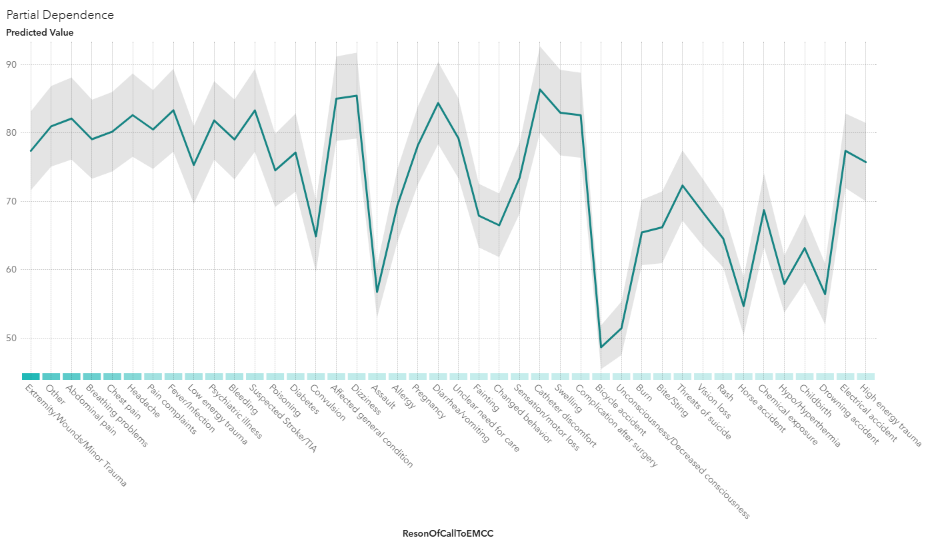


### Reason of Call to EMCC priority level 3


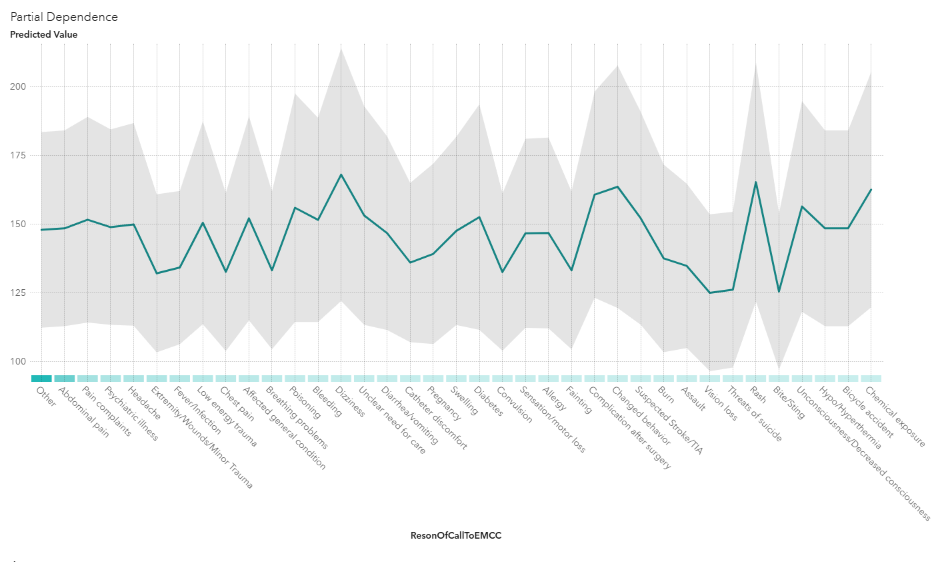


### Hour


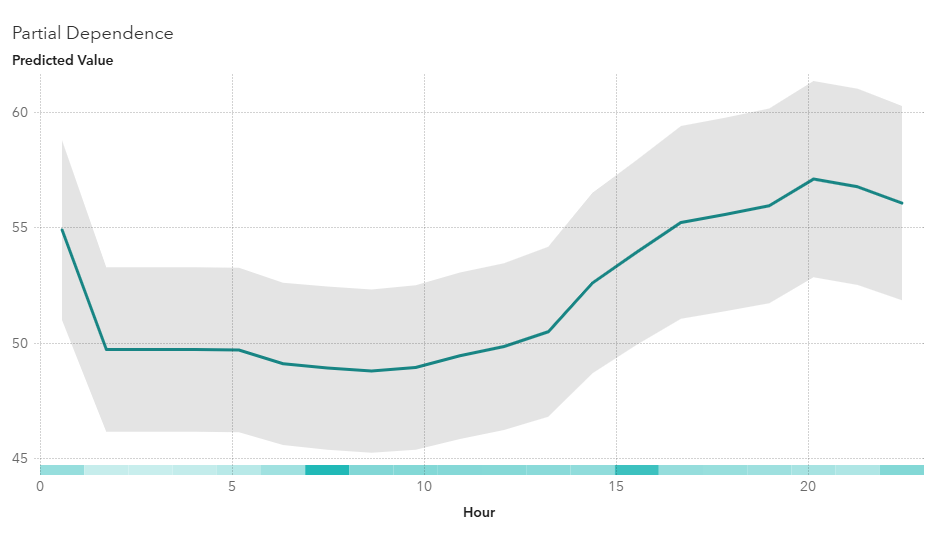


### Hour priority level 1


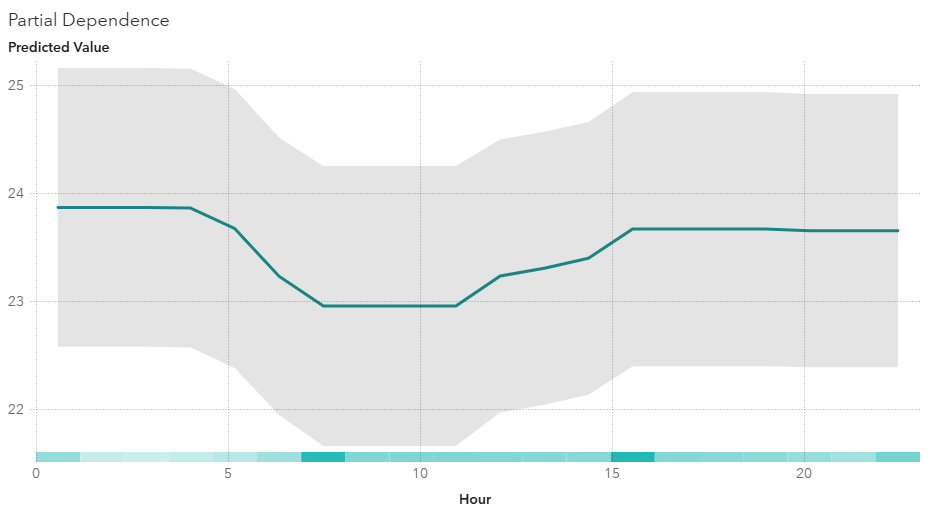


### Hour priority level 2


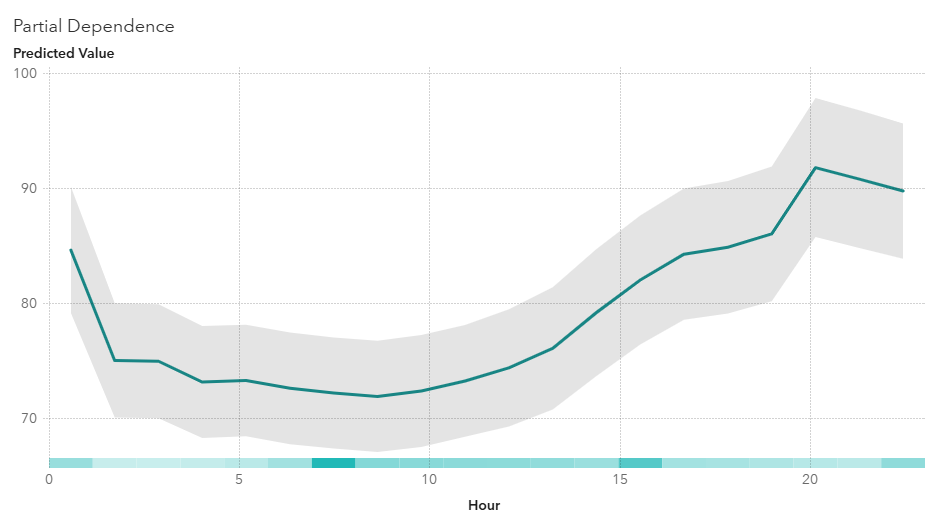


### Hour priority level 3


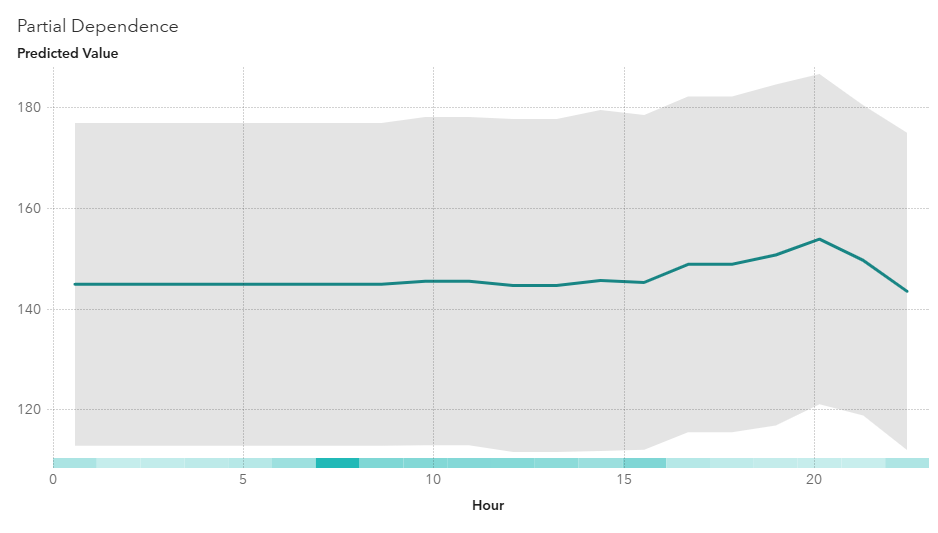


###
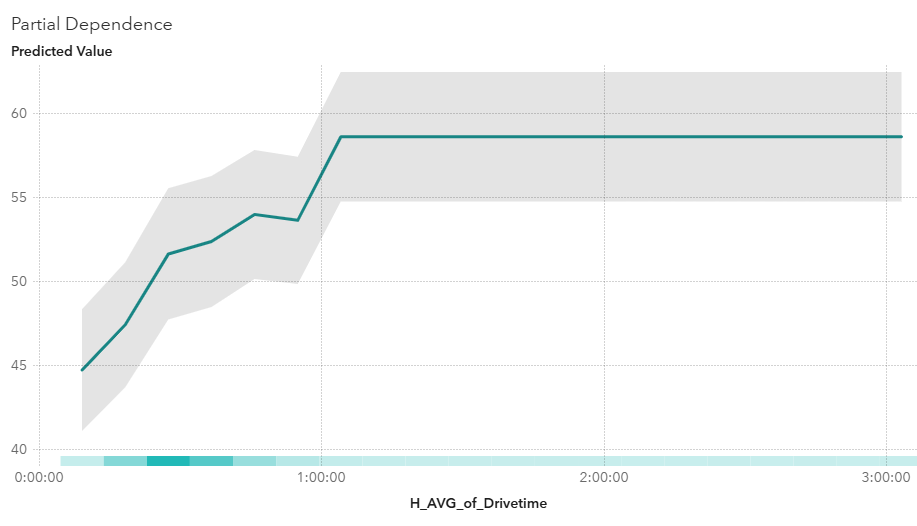
H_AVG_of_Drivetime

### H_AVG_of_Drivetime priority level 1


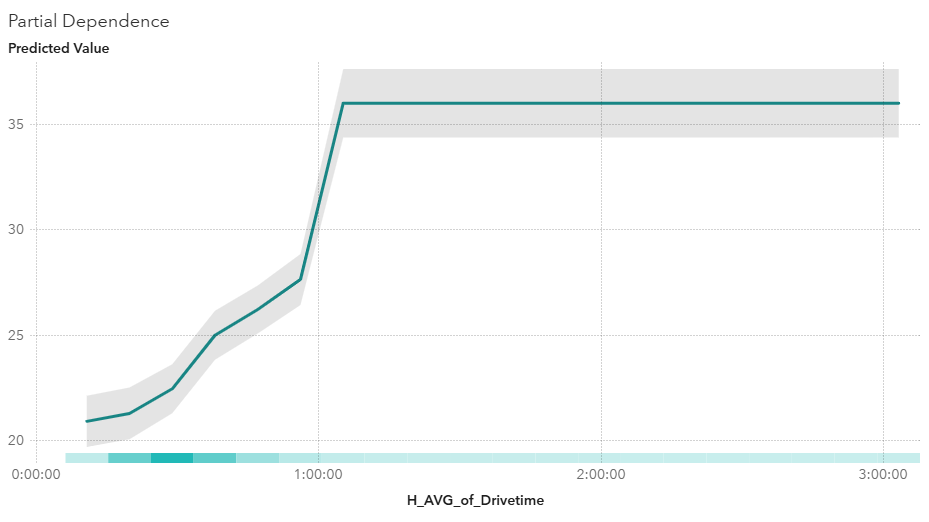


### H_AVG_of_Drivetime priority level 2


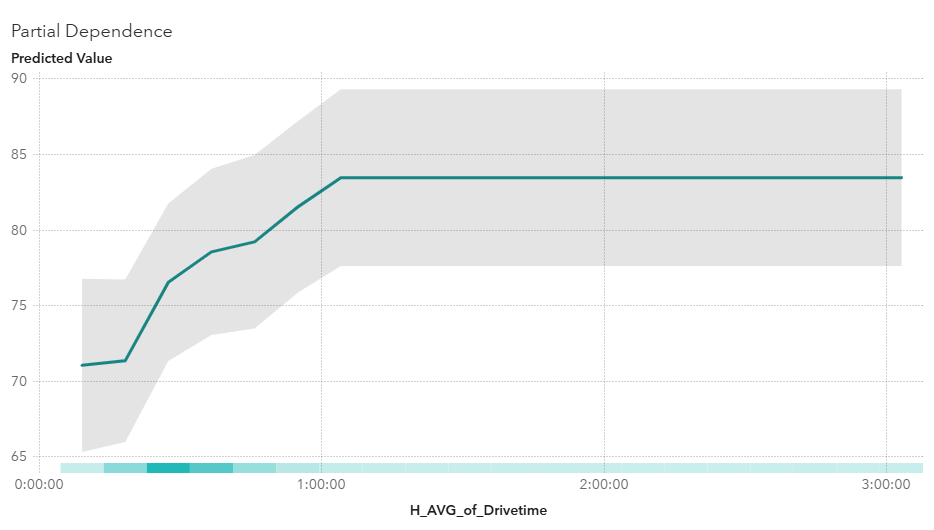


### H_AVG_of_Drivetime priority level 3


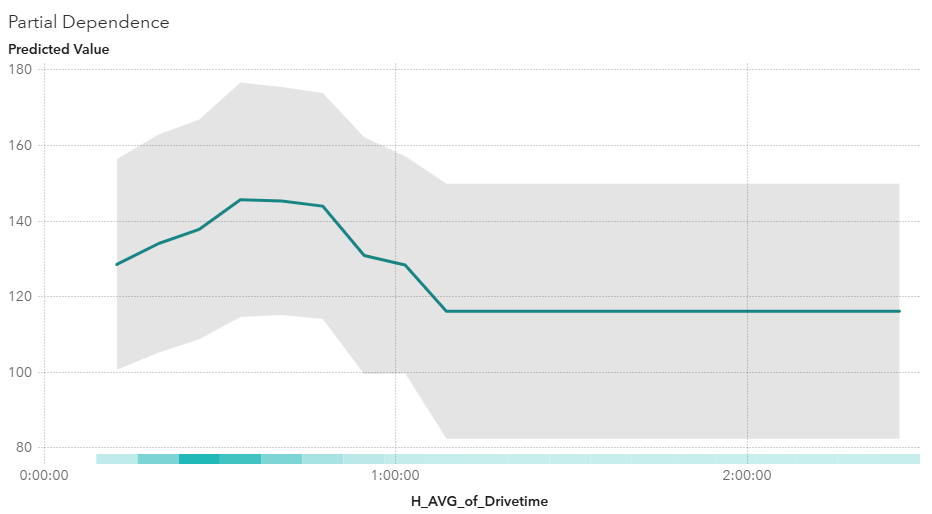


### Month


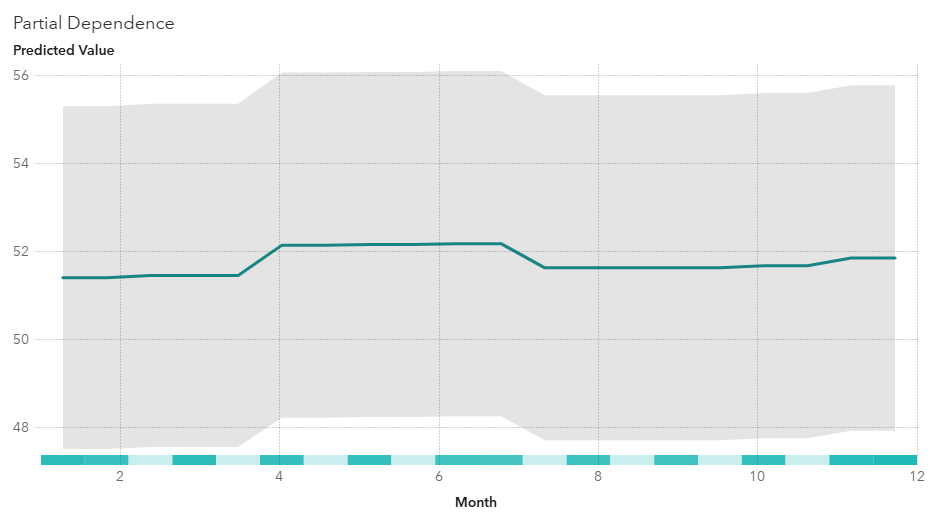


### Month priority level 1


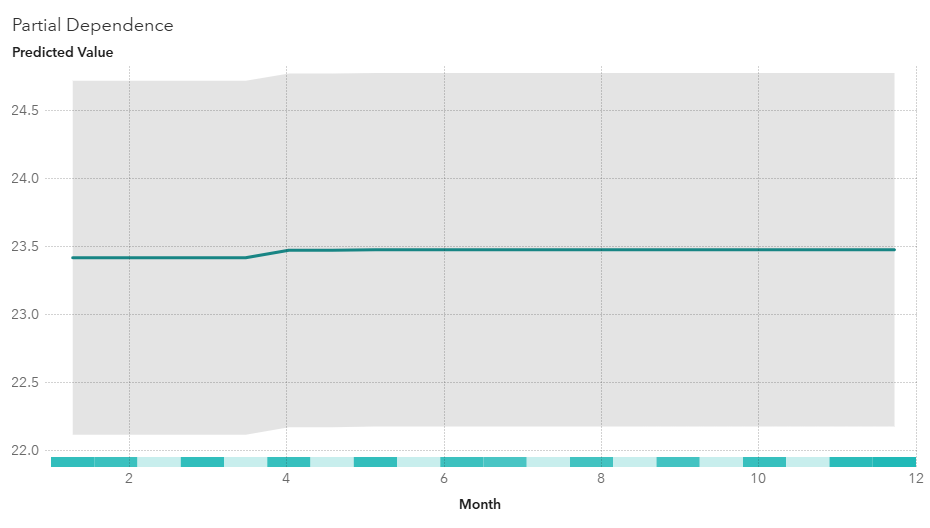


### Month priority level 2


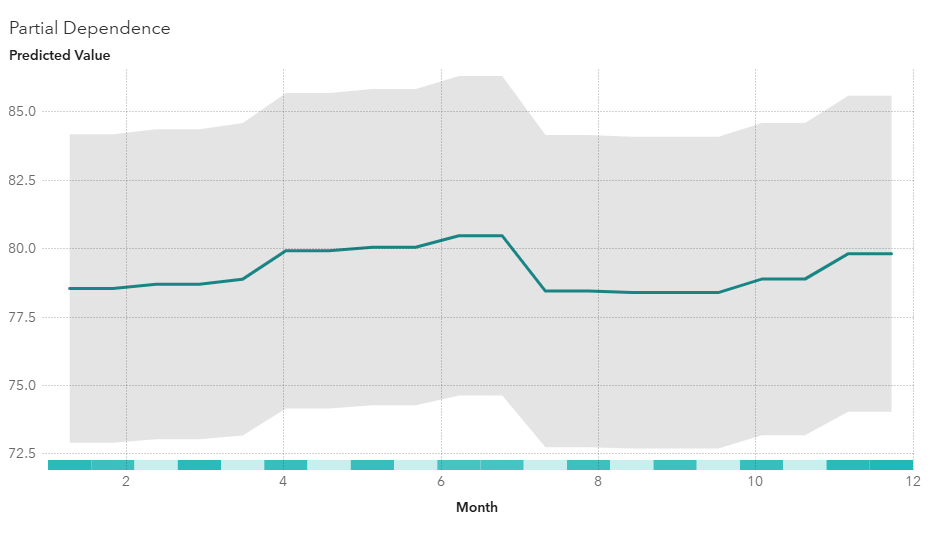


### Month priority level 3


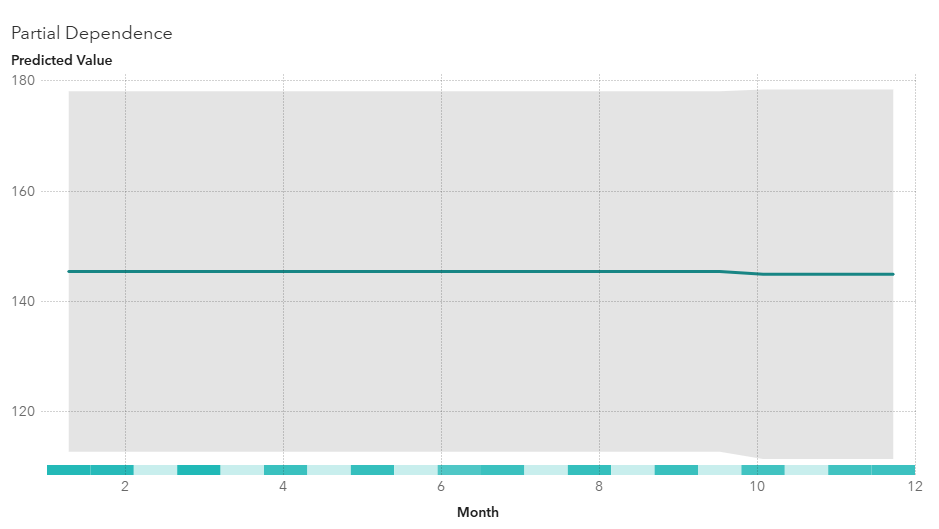


### Locality


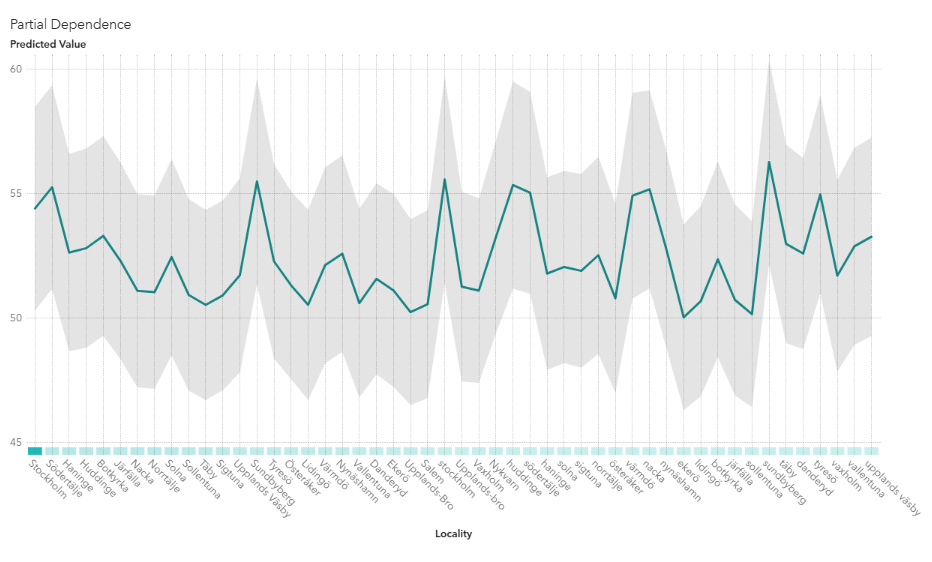


### Locality priority level 1


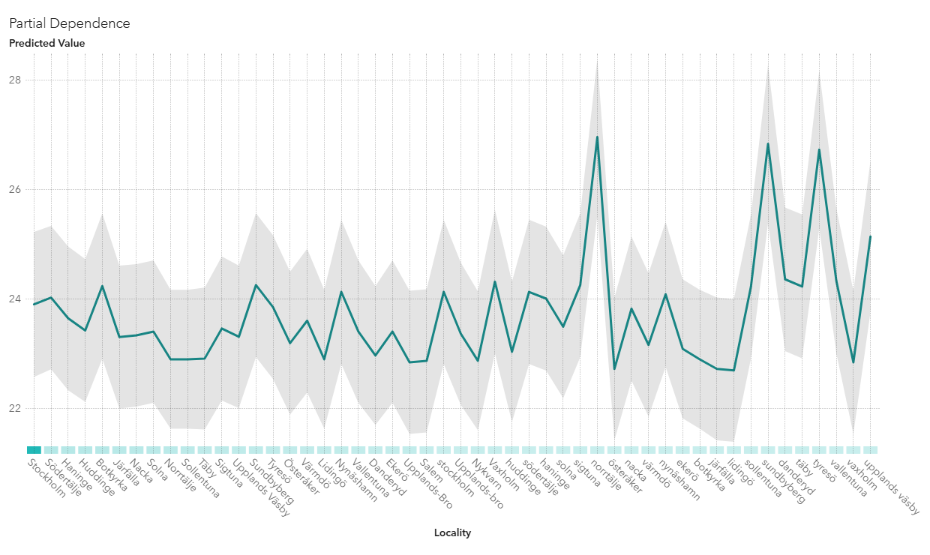


### Locality priority level 2


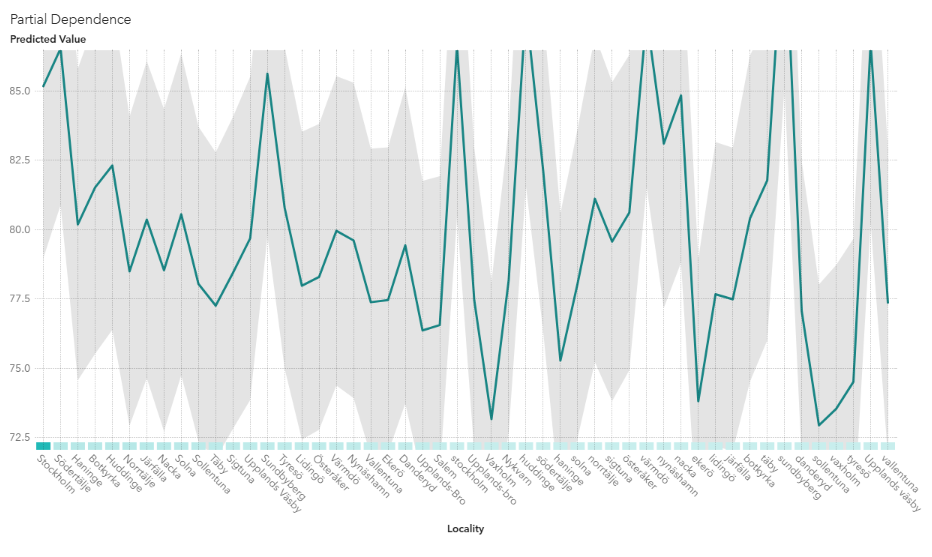


### Locality priority level 3


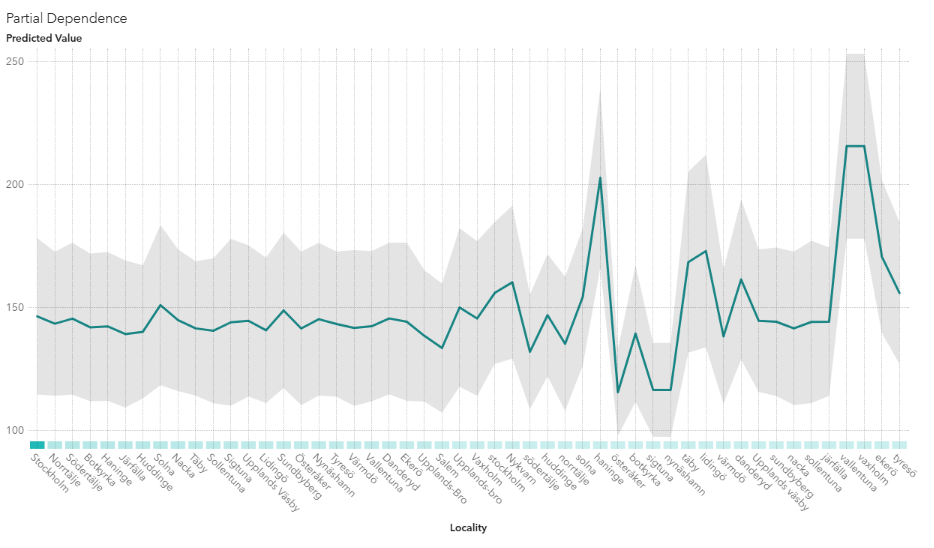


### H_SUM_of_Missions


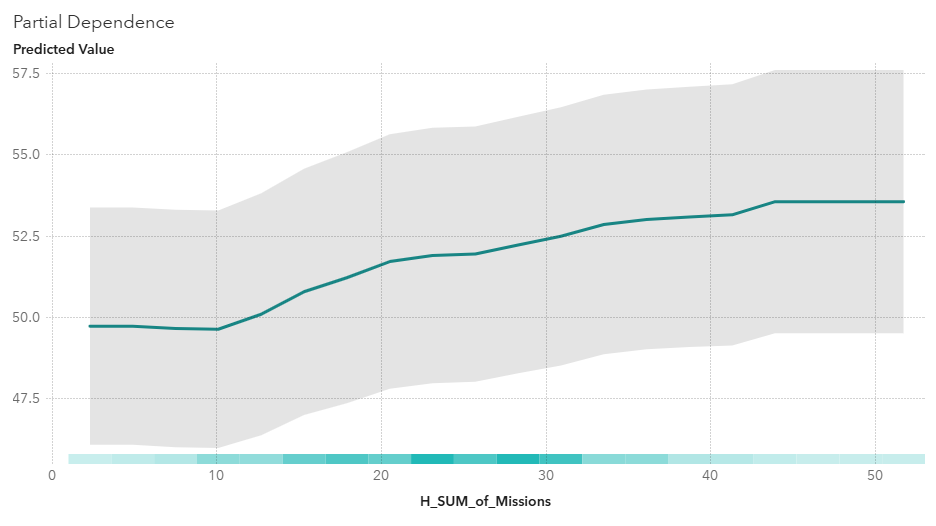


### H_SUM_of_Missions priority level 1


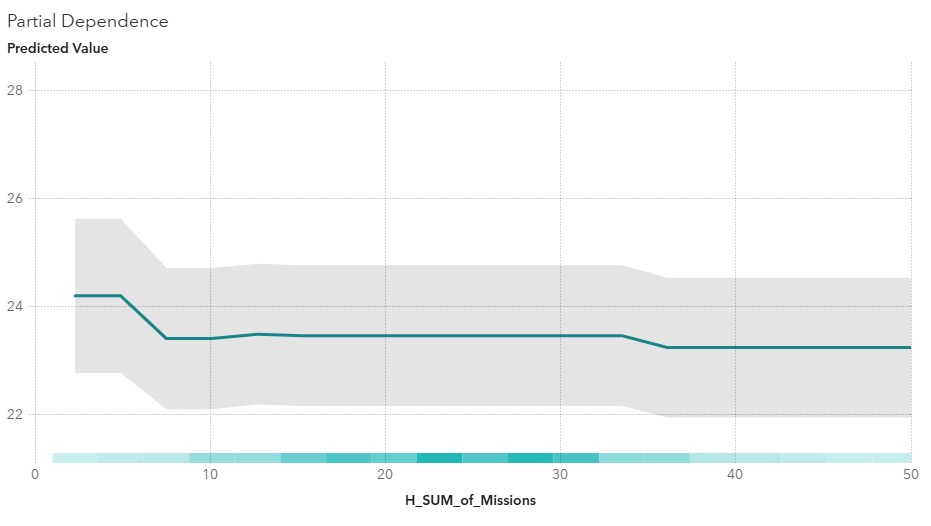


### H_SUM_of_Missions priority level 2


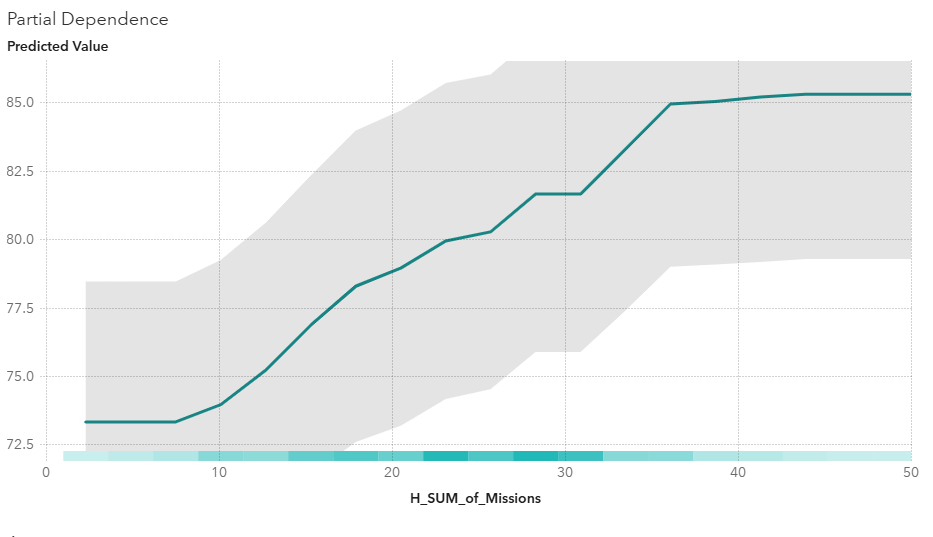


### H_SUM_of_Missions priority level 3


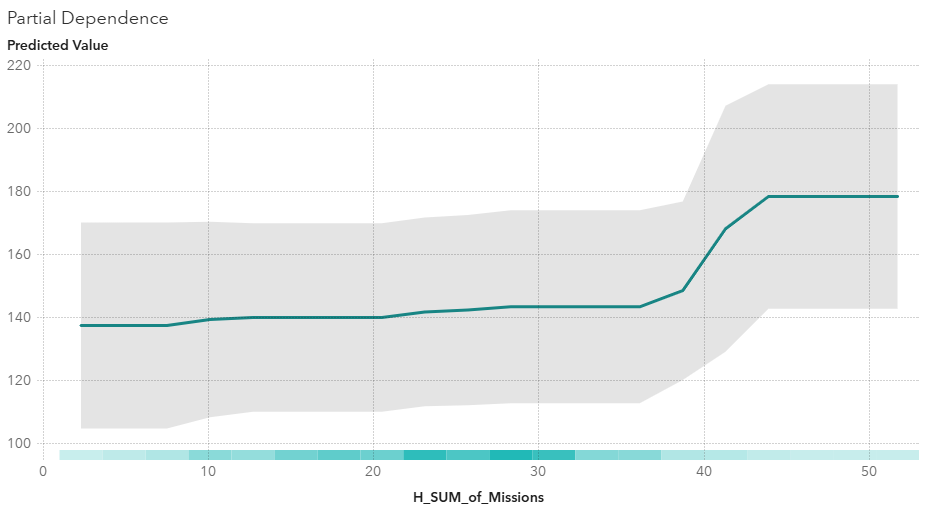


### Weekday


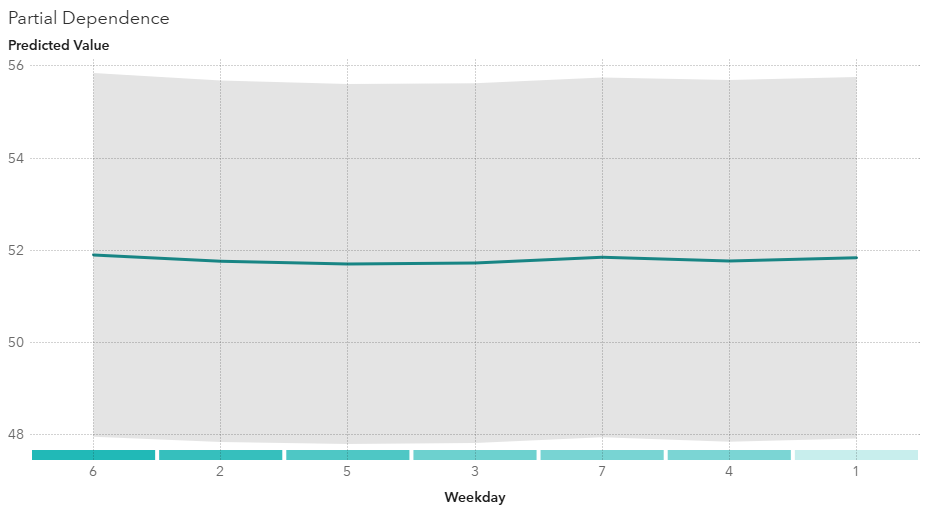


### Weekday priority level 1


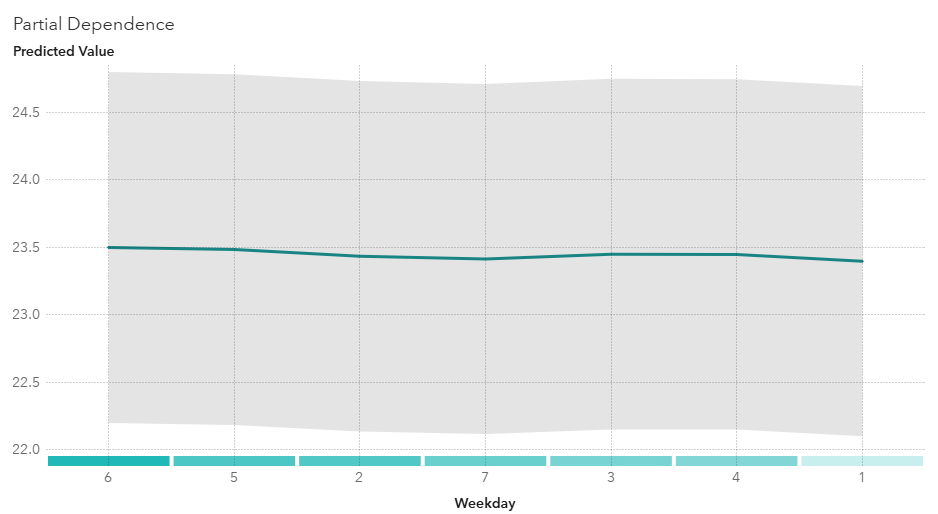


### Weekday priority level 2


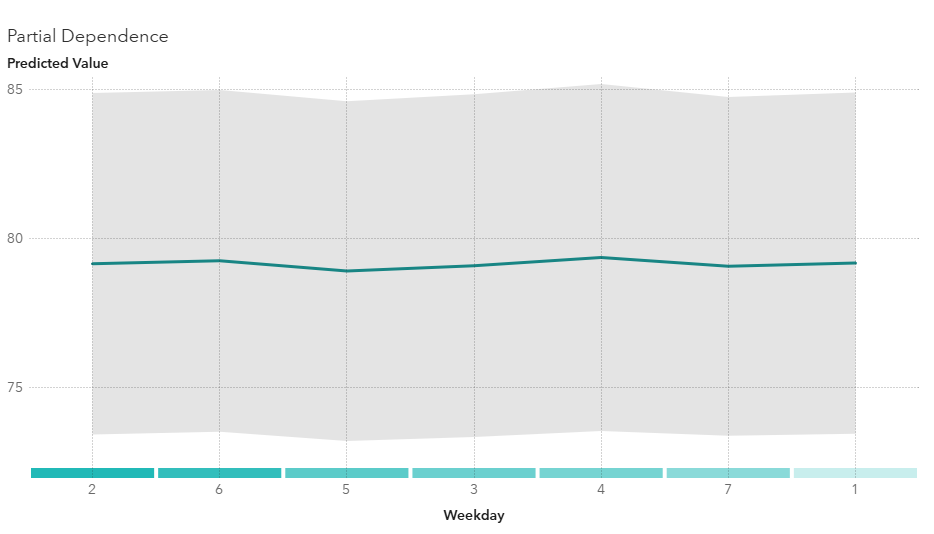


### Weekday priority level 3


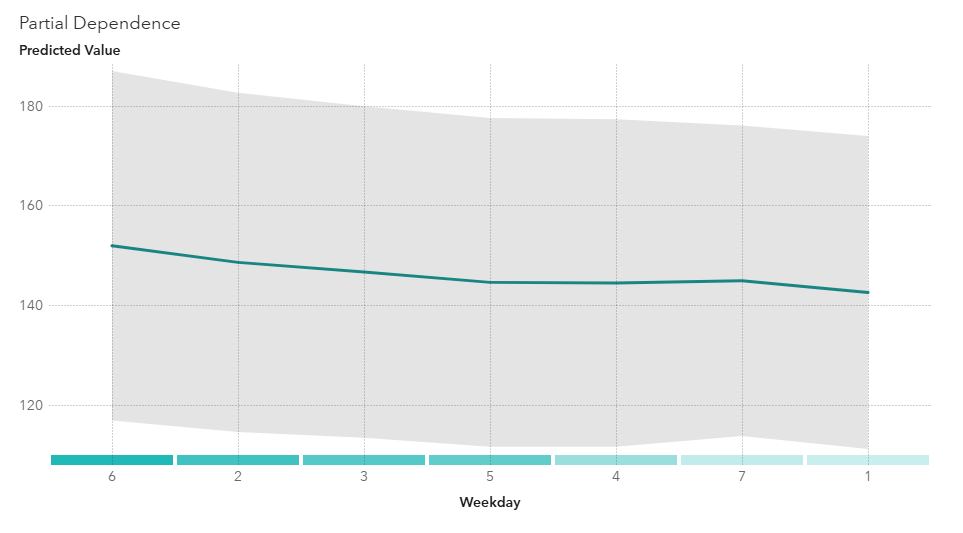


### H_AVG_of_DistArrivalkm


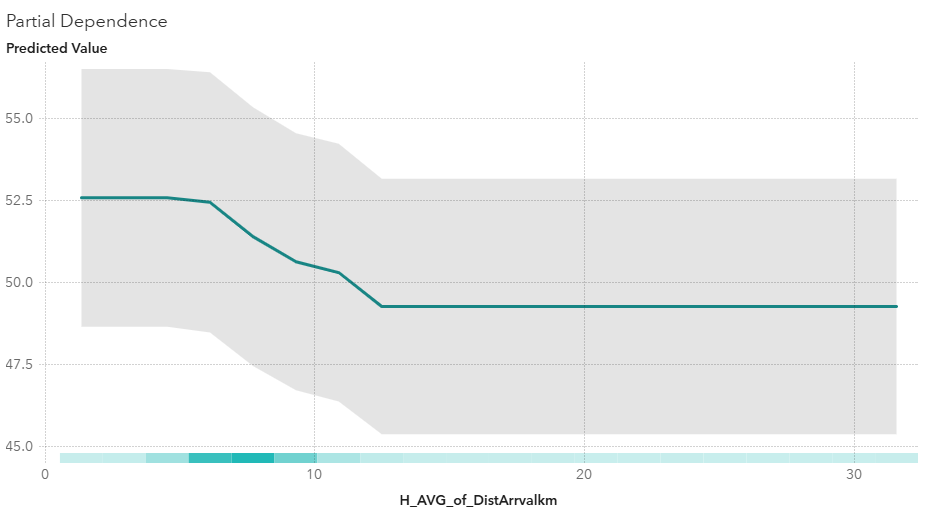


### H_AVG_of_DistArrivalkm priority level 1


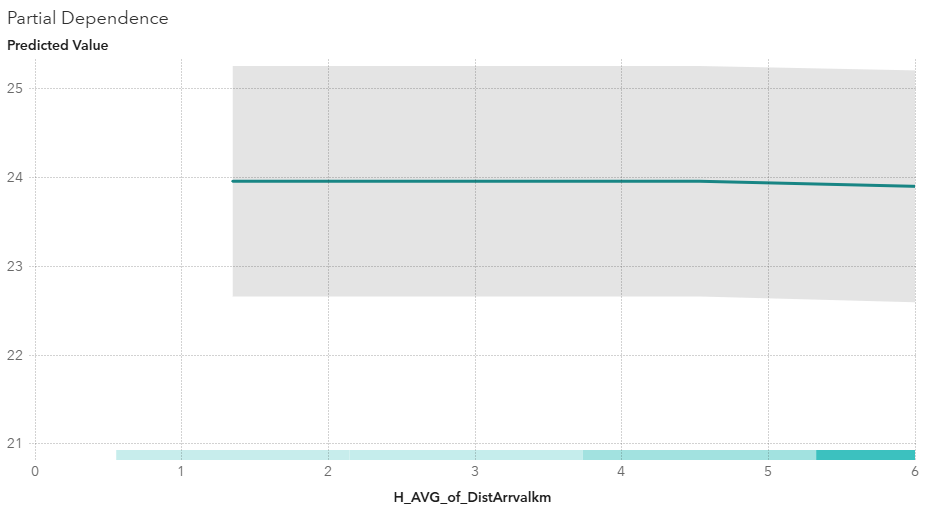


### H_AVG_of_DistArrivalkm priority level 2


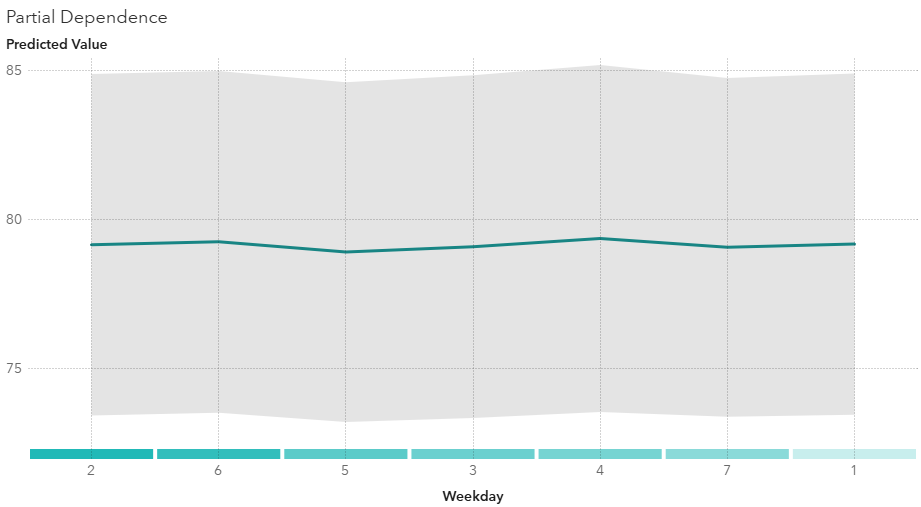


### H_AVG_of_DistArrivalkm priority level 3


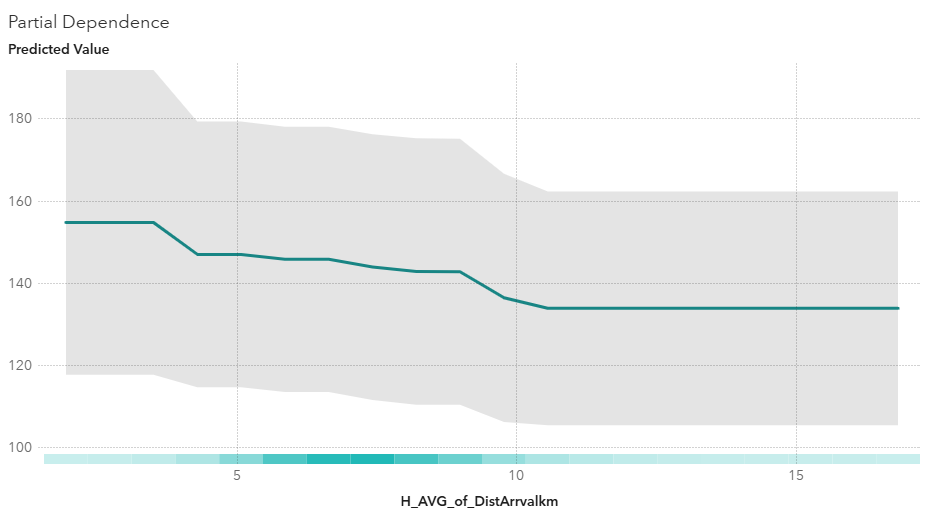


### Airtemperature_max


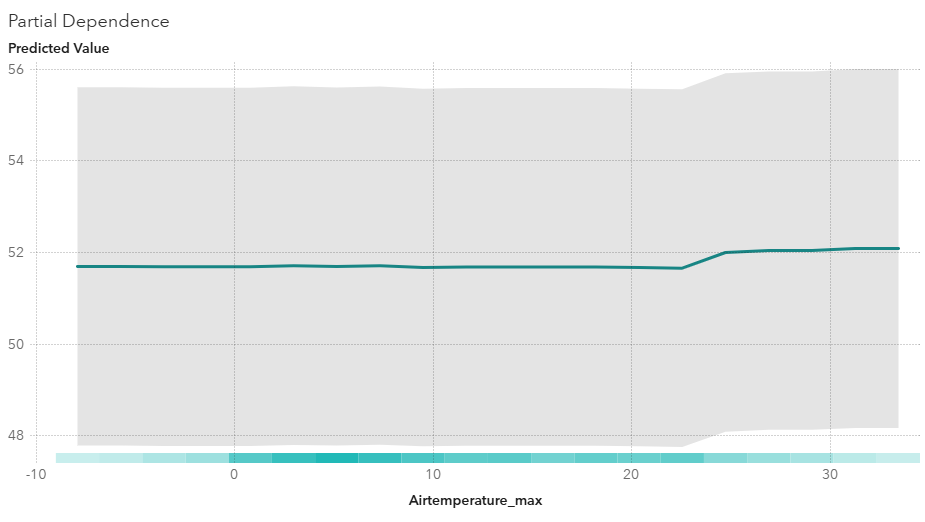


### Airtemperature_max priority level 1


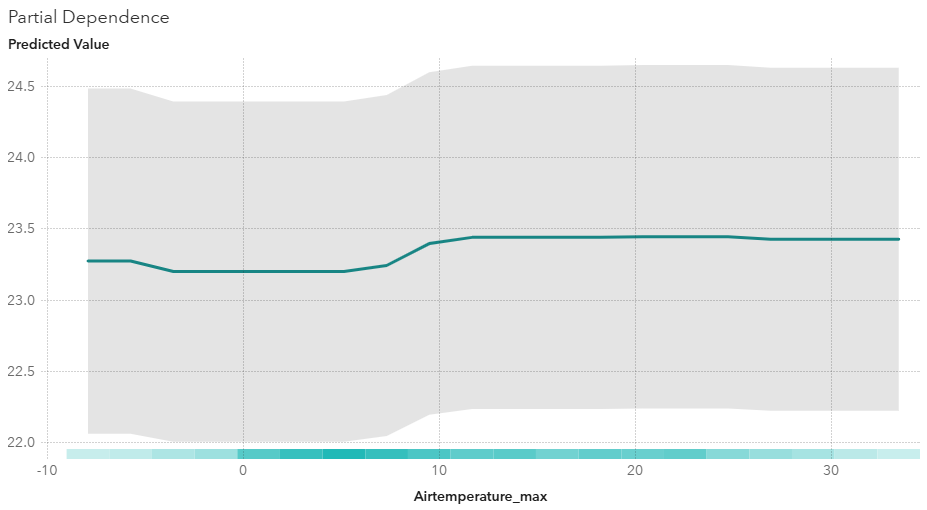


### Airtemperature_max priority level 2


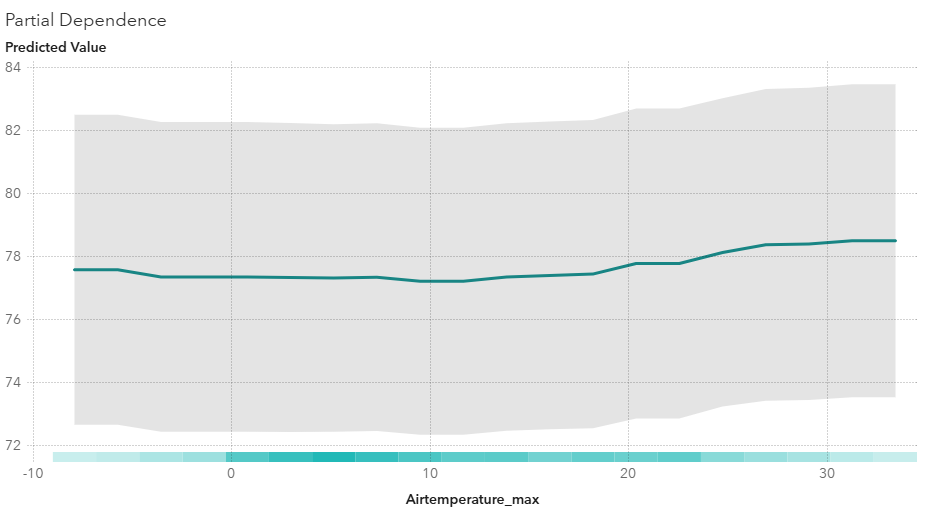


### Airtemperature_max priority level 3


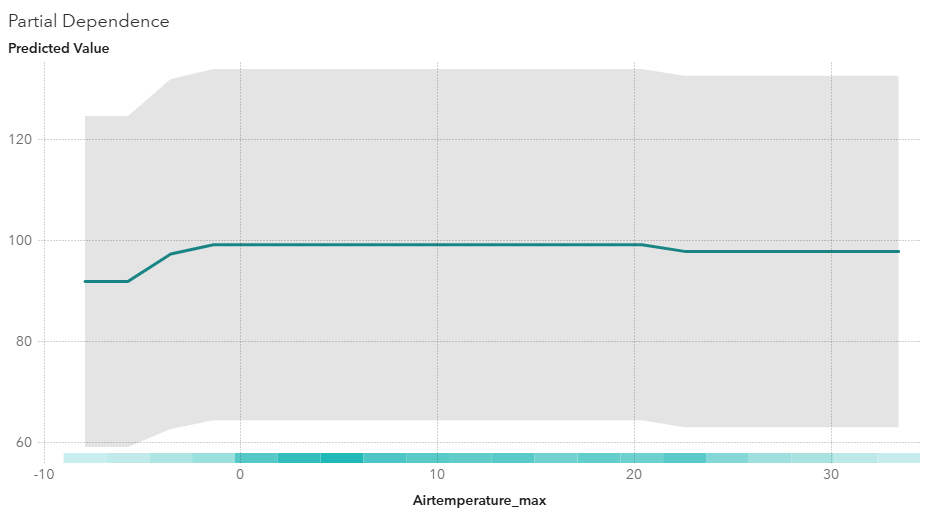


### Airtemperature_min


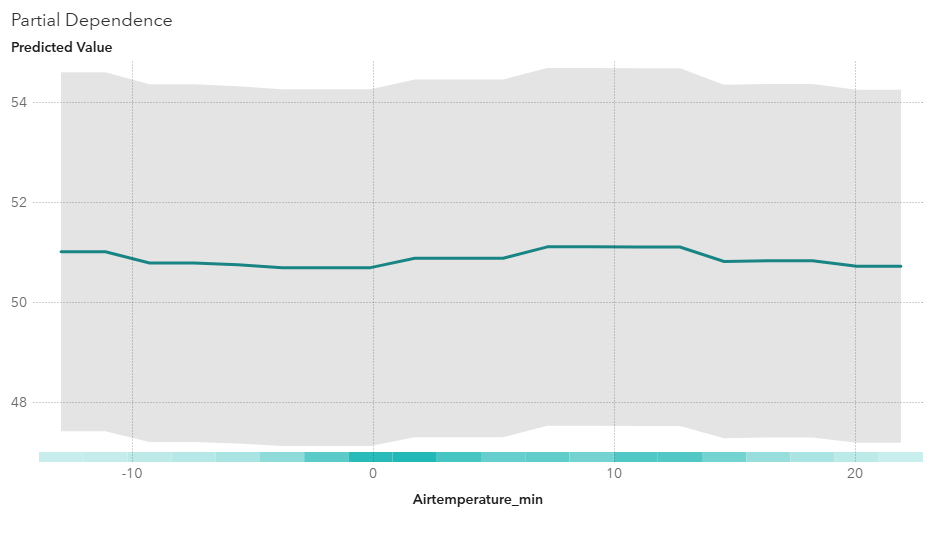


### Airtemperature_min priority level 1


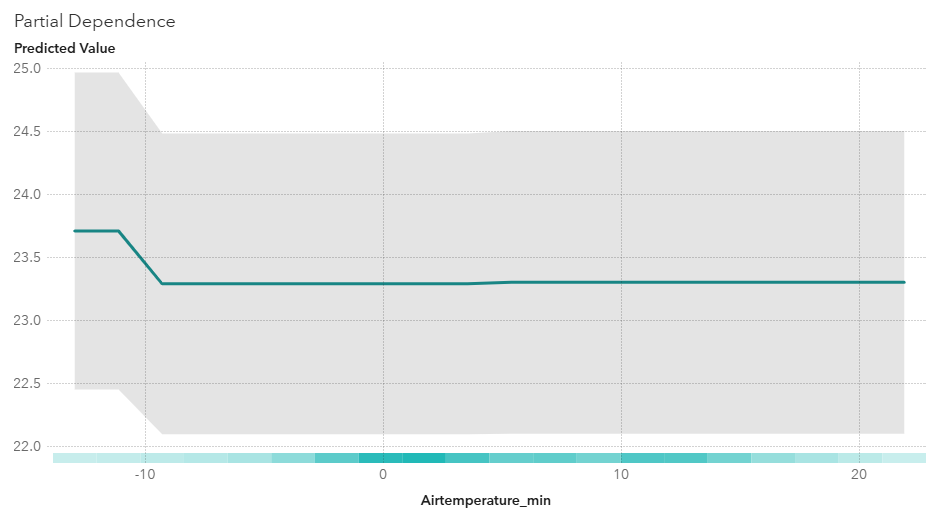


### Airtemperature_min priority level 2


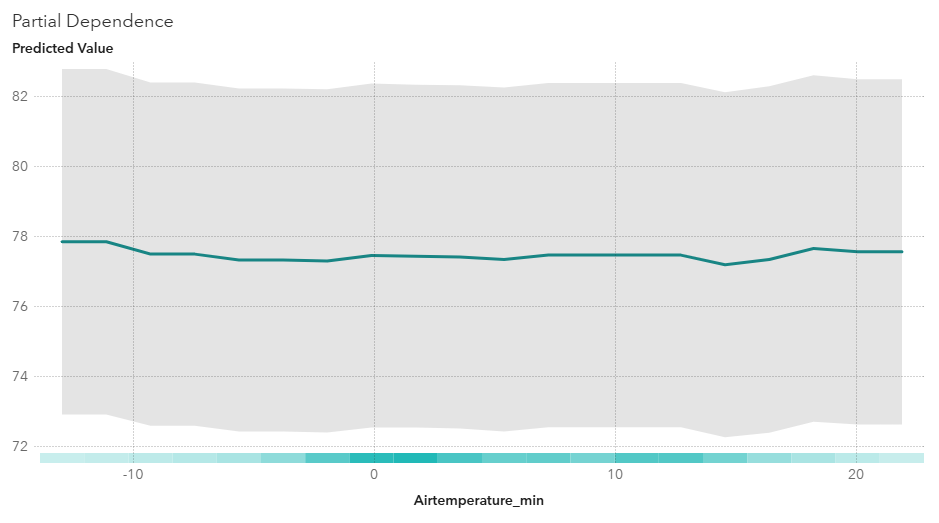


### Airtemperature_min priority level 3


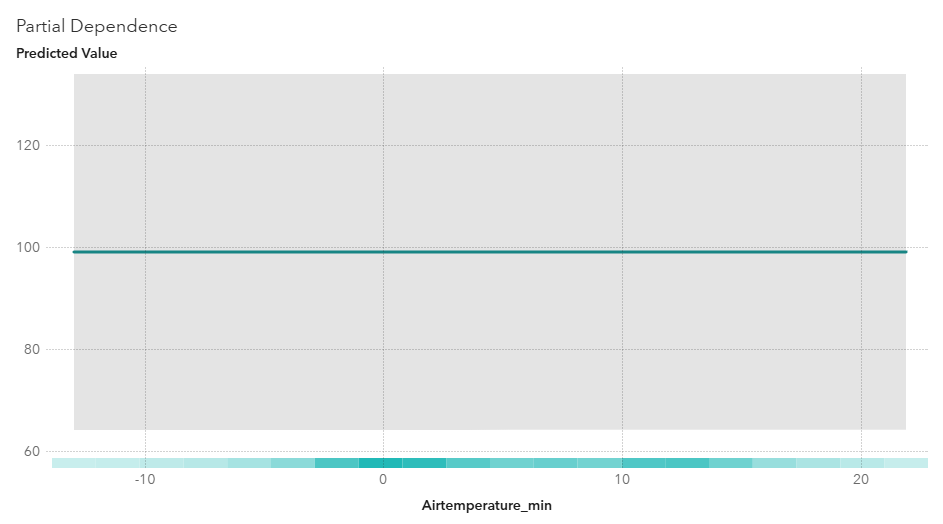


### H_SUM_of_MissionCanc


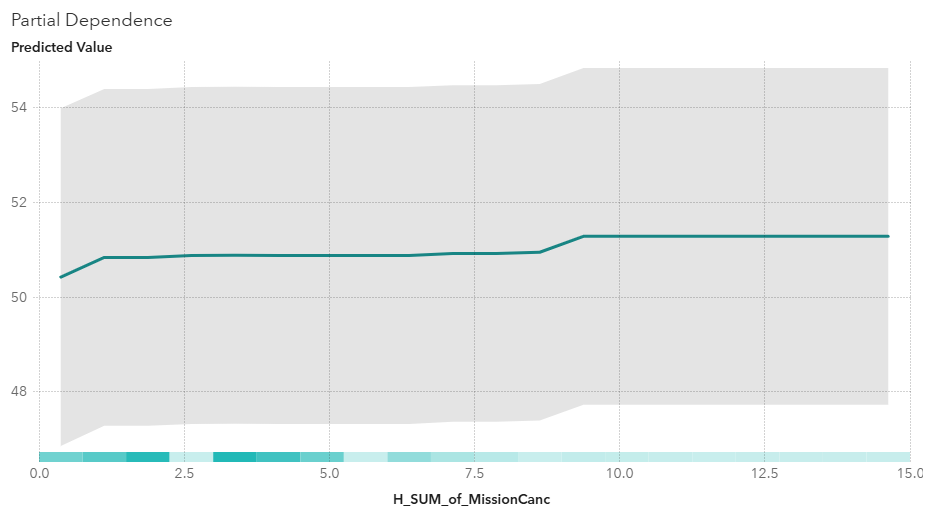


### H_SUM_of_MissionCanc priority level 1


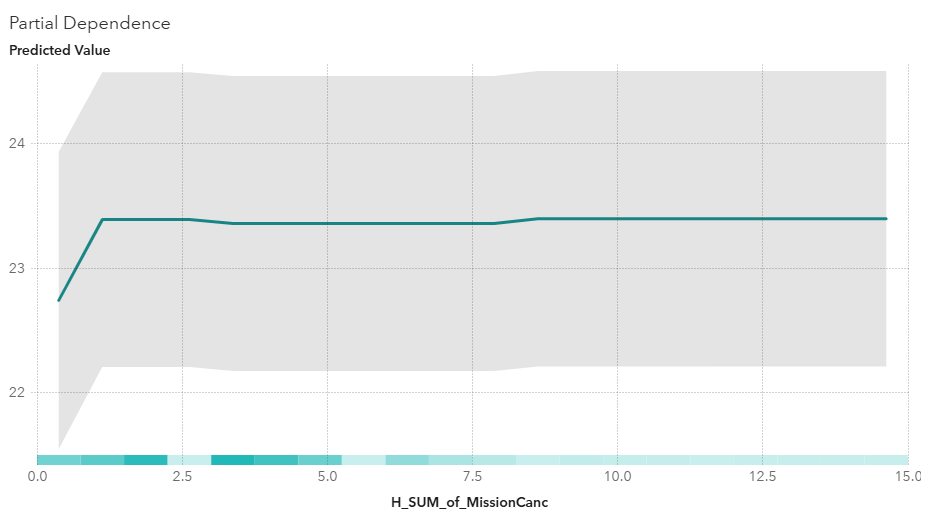


### H_SUM_of_MissionCanc priority level 2


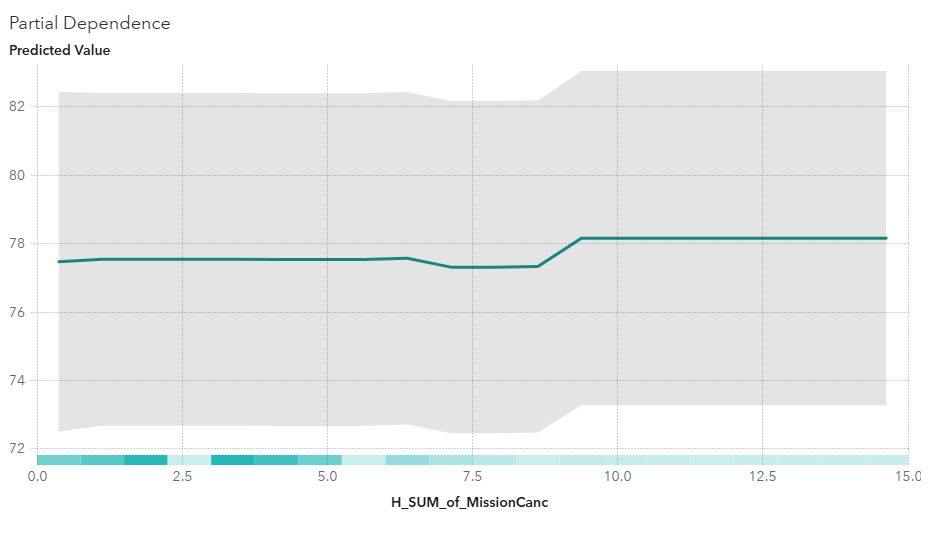


### H_SUM_of_MissionCanc priority level 3


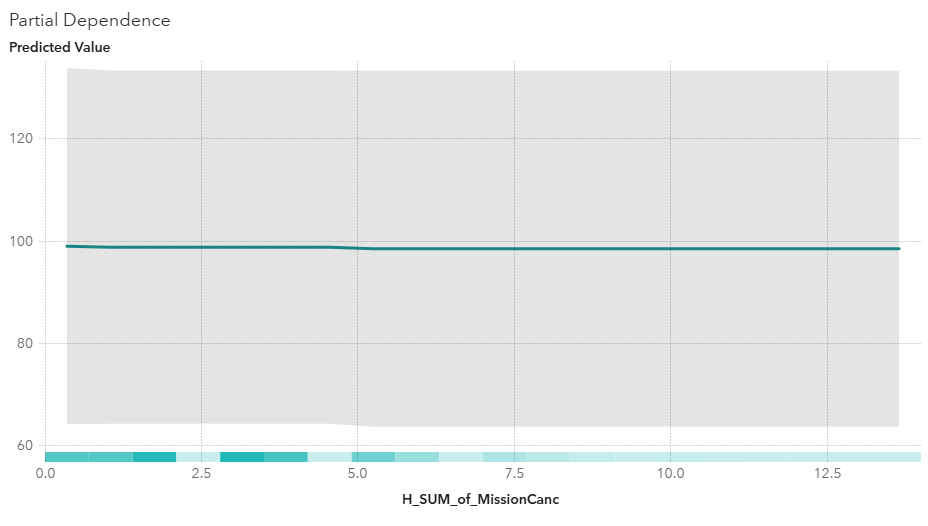


### H_AVG_of_Onscenetime


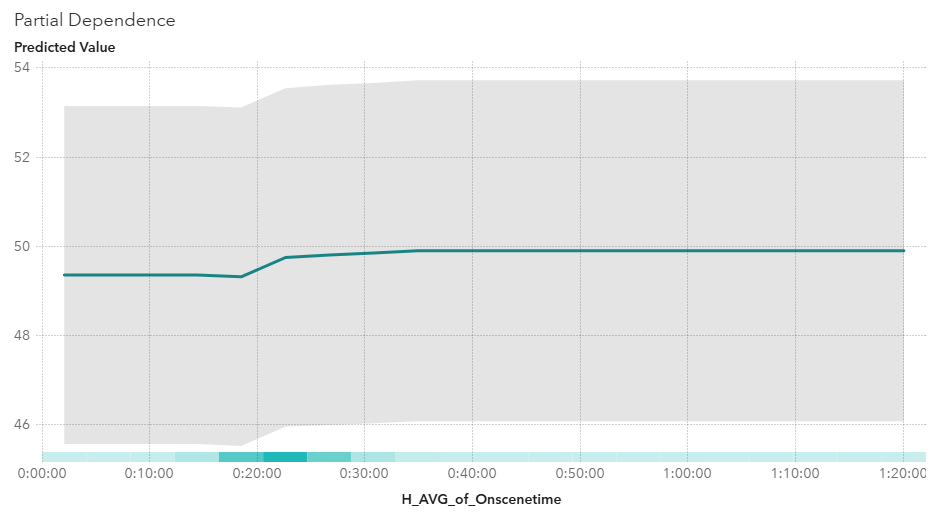


### H_AVG_of_Onscenetime priority level 1


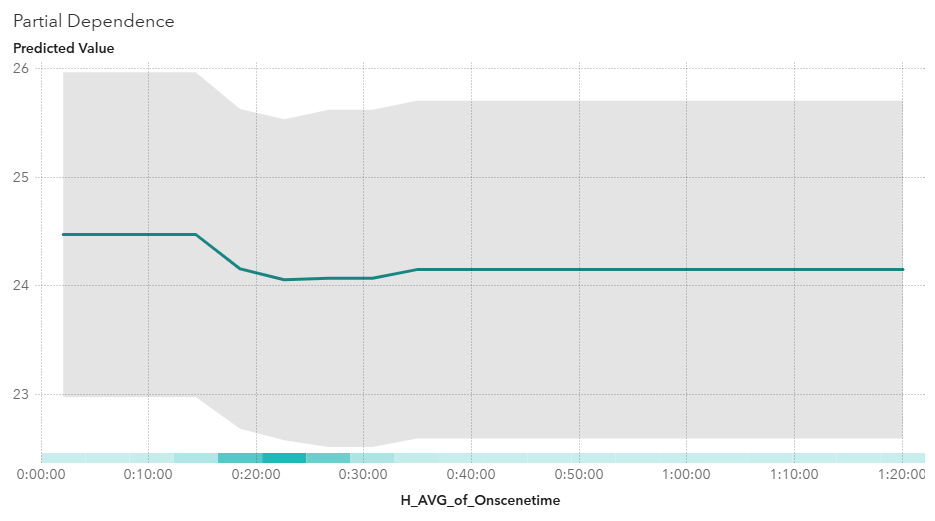


### H_AVG_of_Onscenetime priority level 2


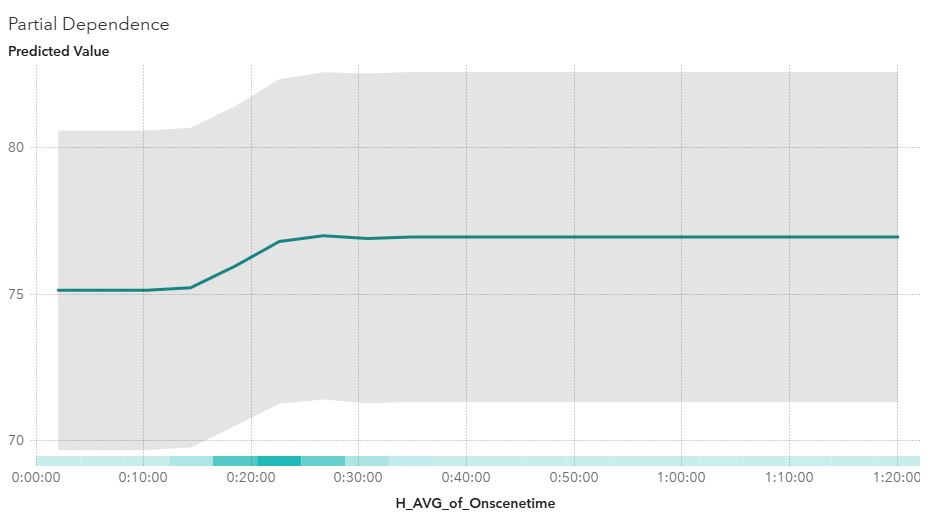


### H_AVG_of_Onscenetime priority level 3


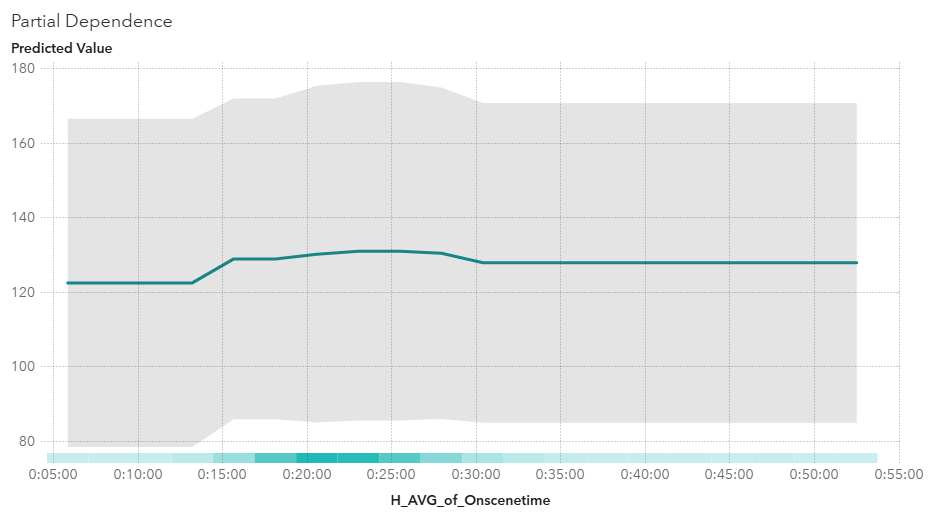


### H_SUM_of_Resources


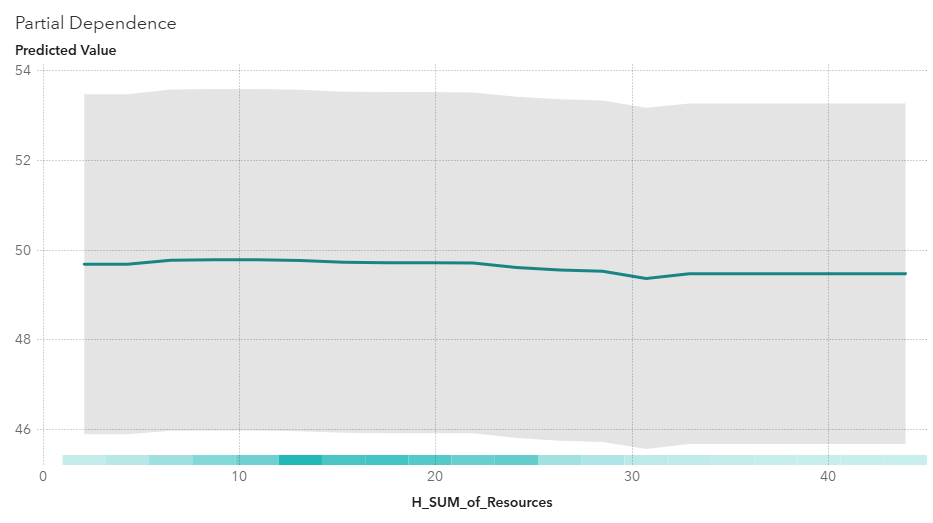


### H_SUM_of_Resources priority level 1


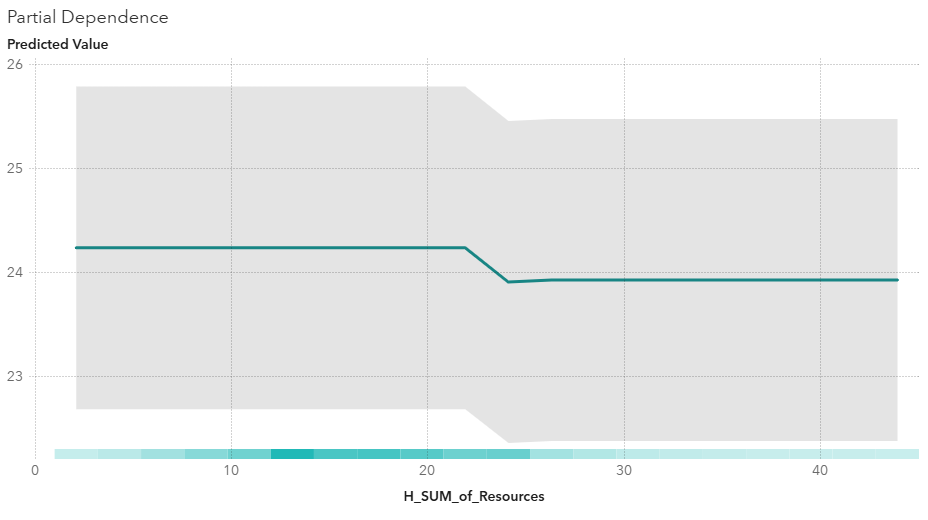


### H_SUM_of_Resources priority level 2


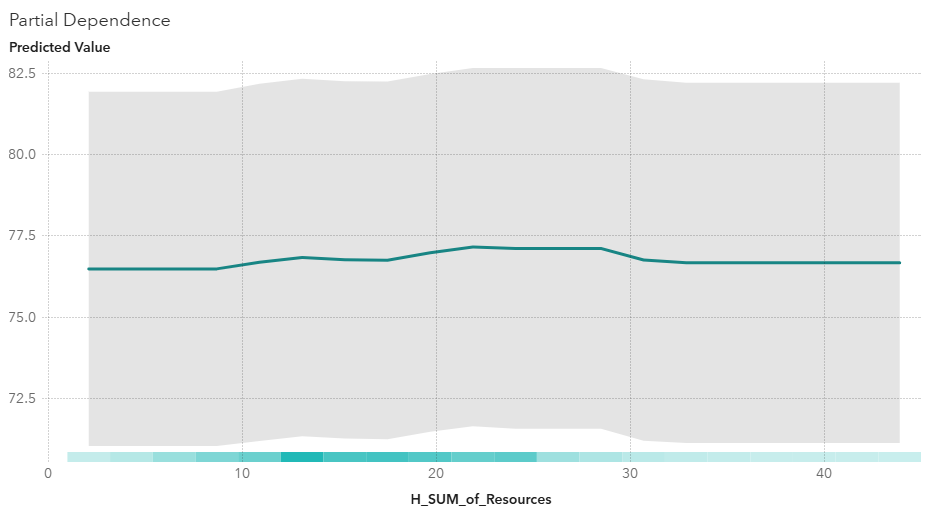


### H_SUM_of_Resources priority level 3


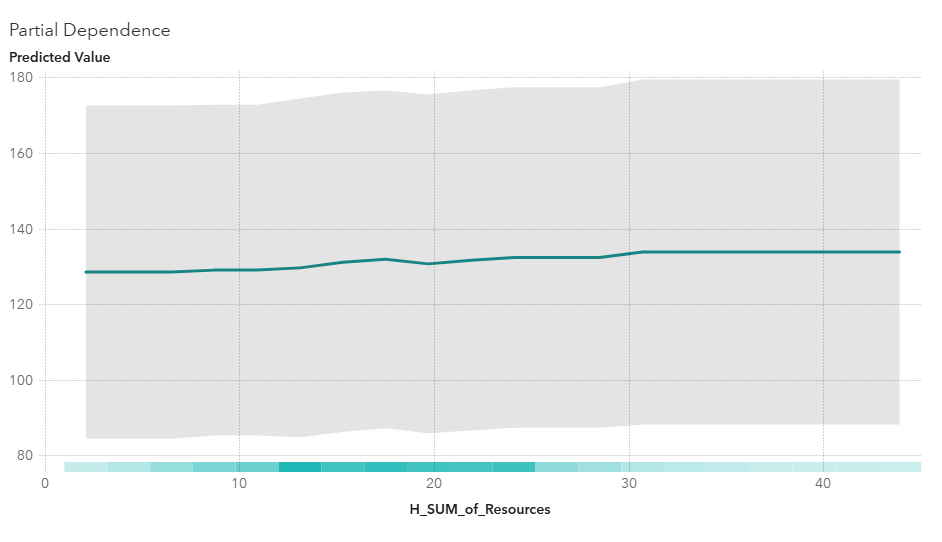


### PrecipitationType


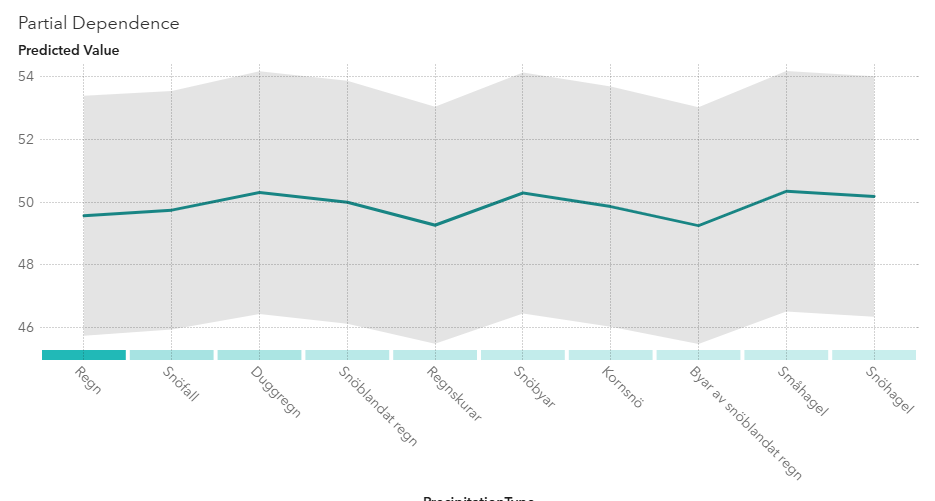


### PrecipitationType priority level 1


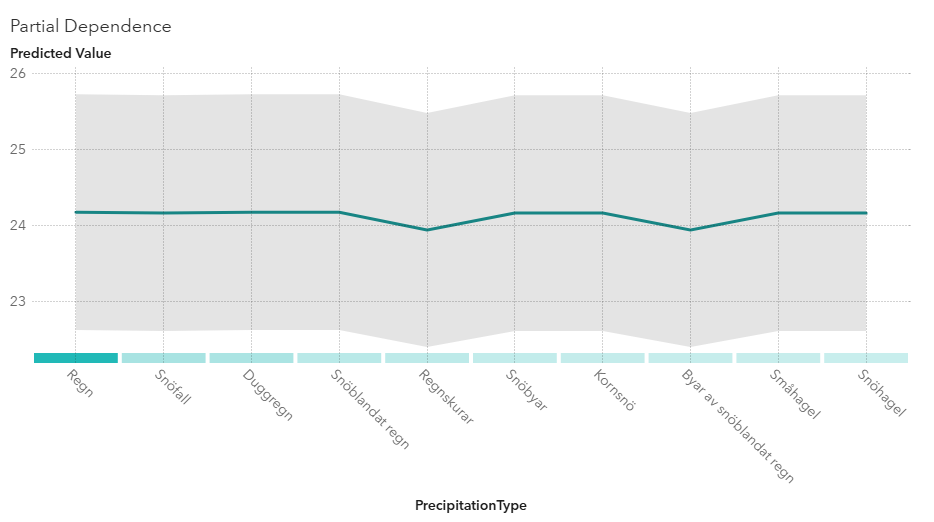


### PrecipitationType priority level 2


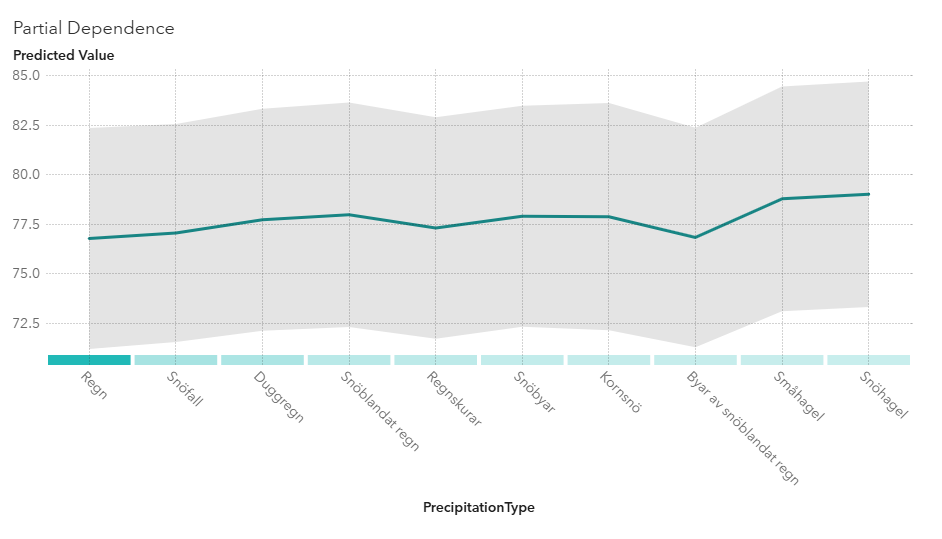


### PrecipitationType priority level 3


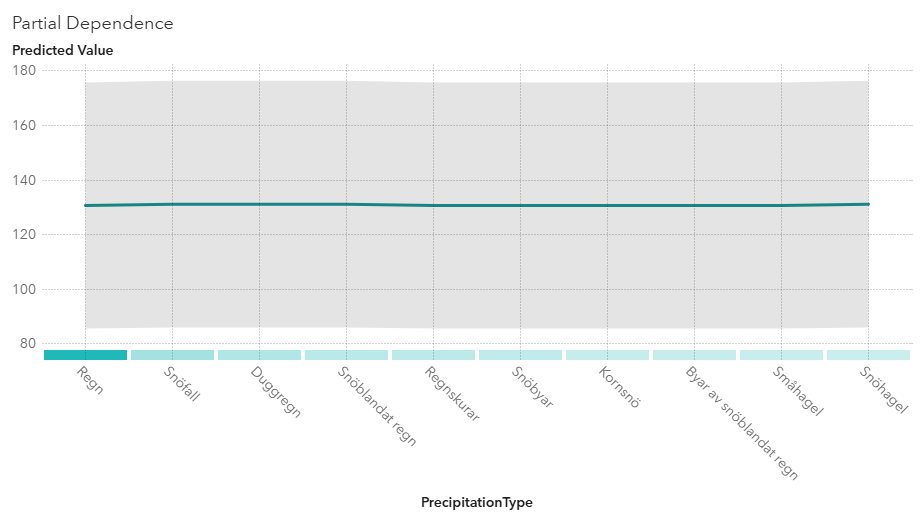


### Airtemperature


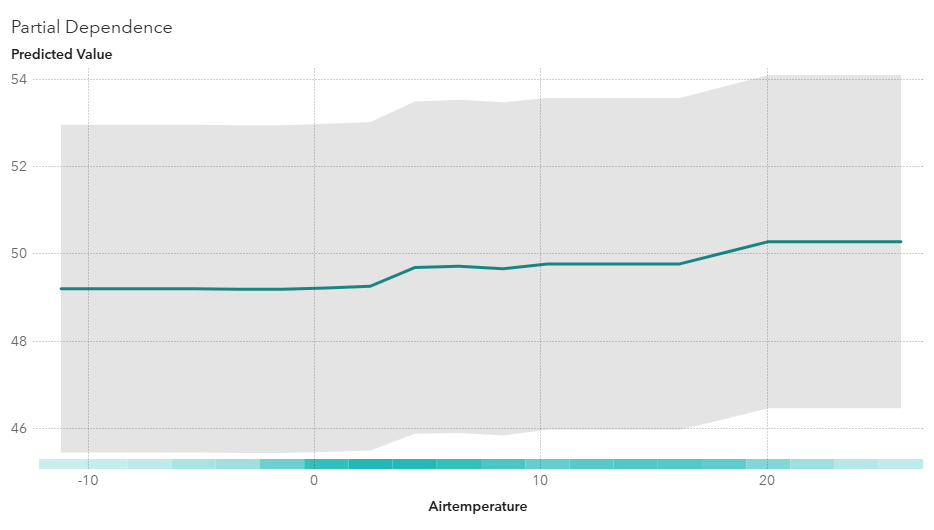


### Airtemperature priority level 1


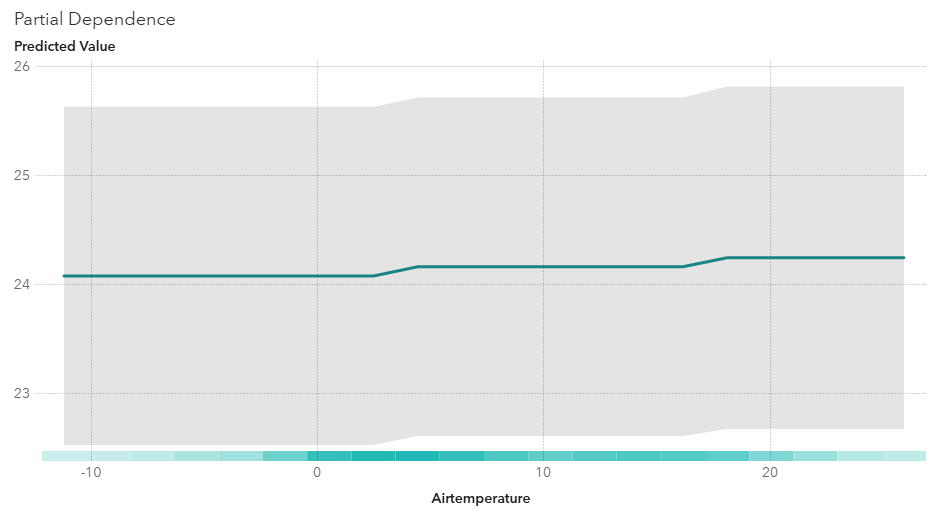


### Airtemperature priority level 2


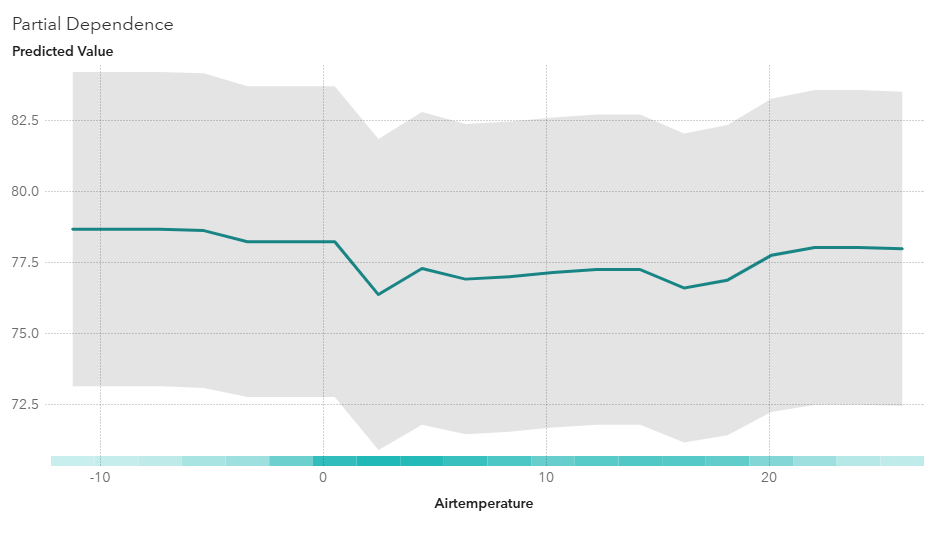


### Airtemperature priority level 3


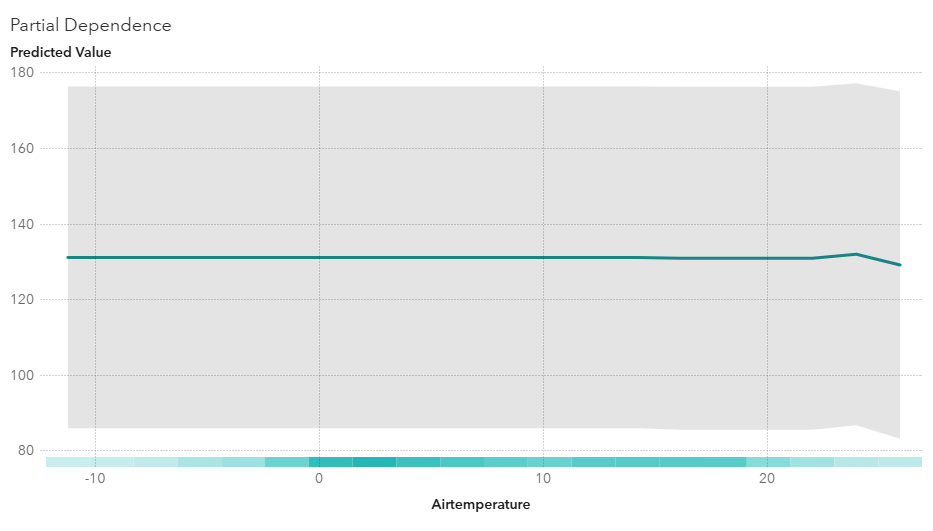


### H_AVG_of_Deliverytime


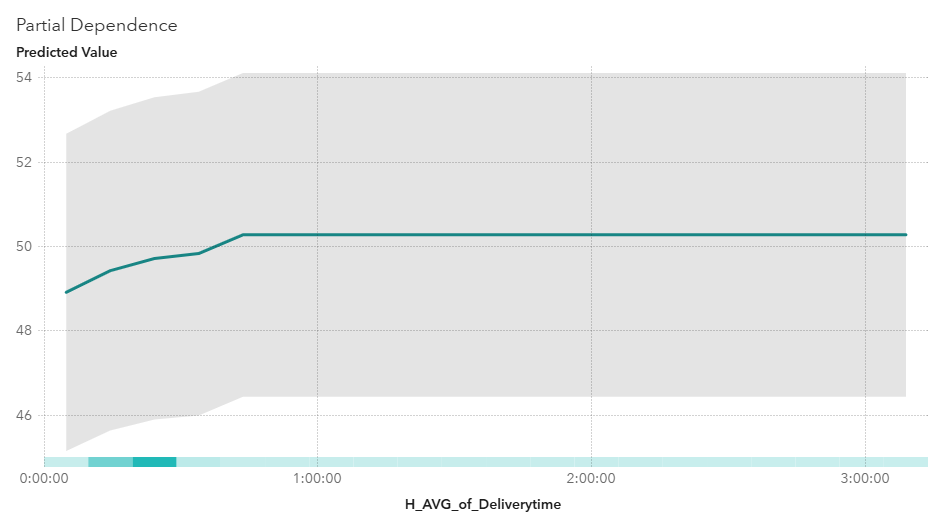


### H_AVG_of_Deliverytime priority level 1


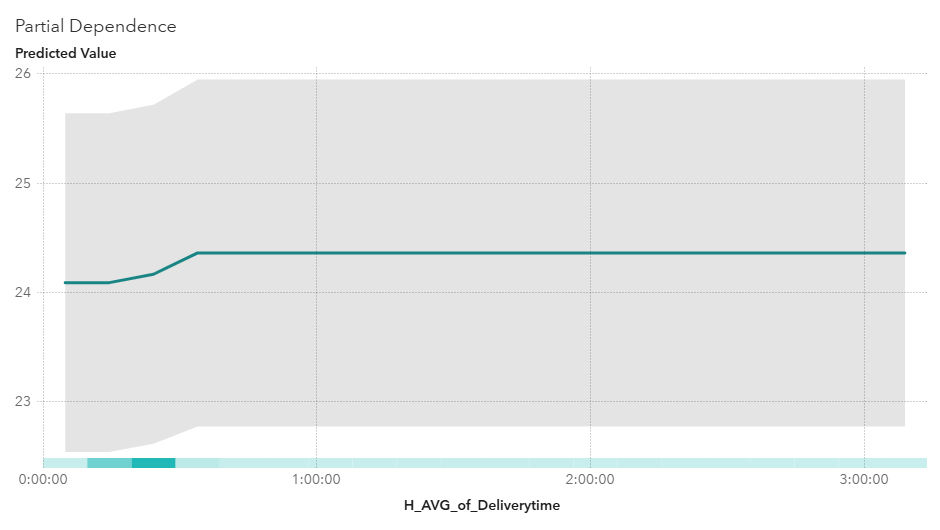


### H_AVG_of_Deliverytime priority level 2


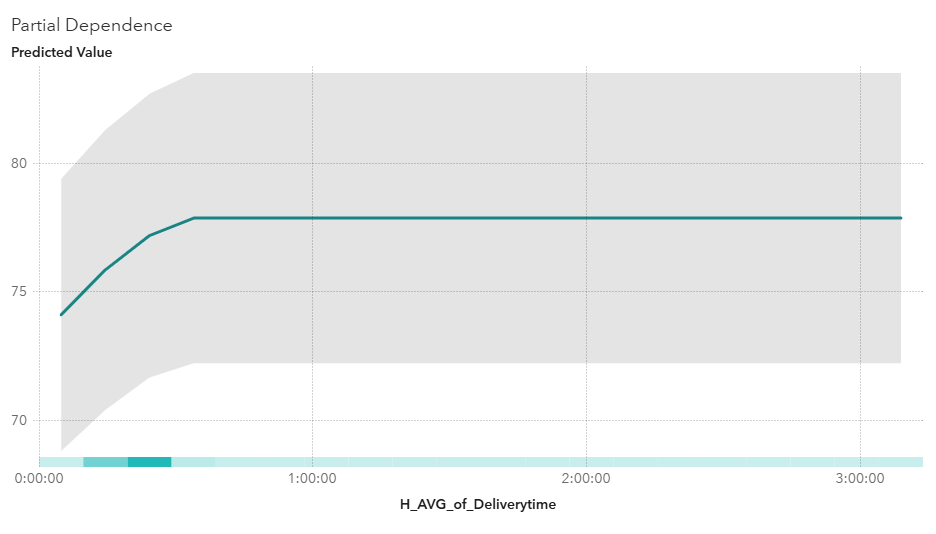


### H_AVG_of_Deliverytime priority level 3


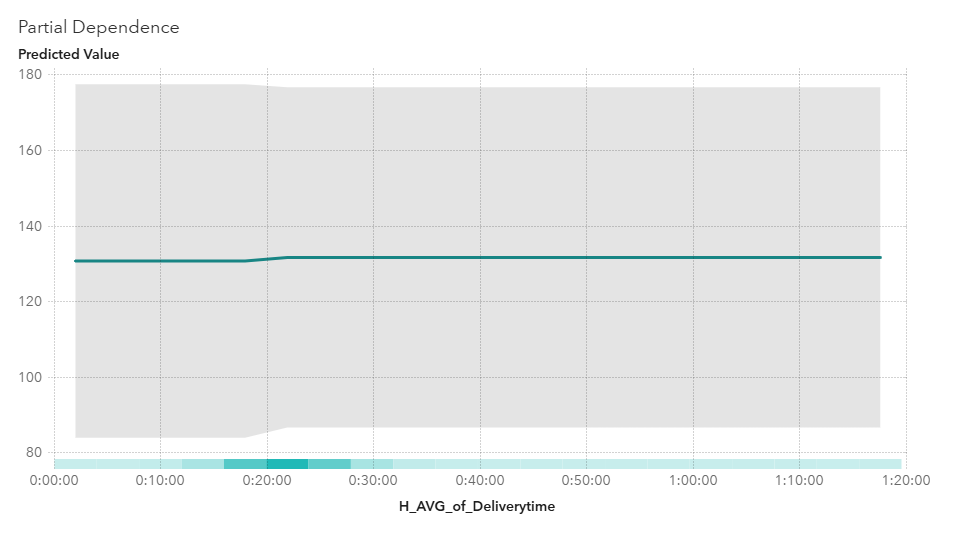


### H_AVG_of_Transportationtime


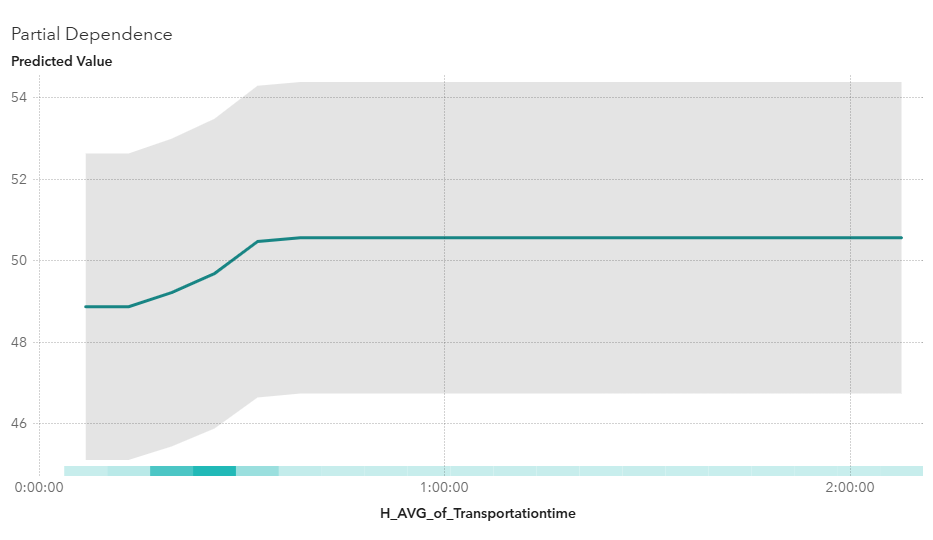


### H_AVG_of_Transportationtime priority level 1


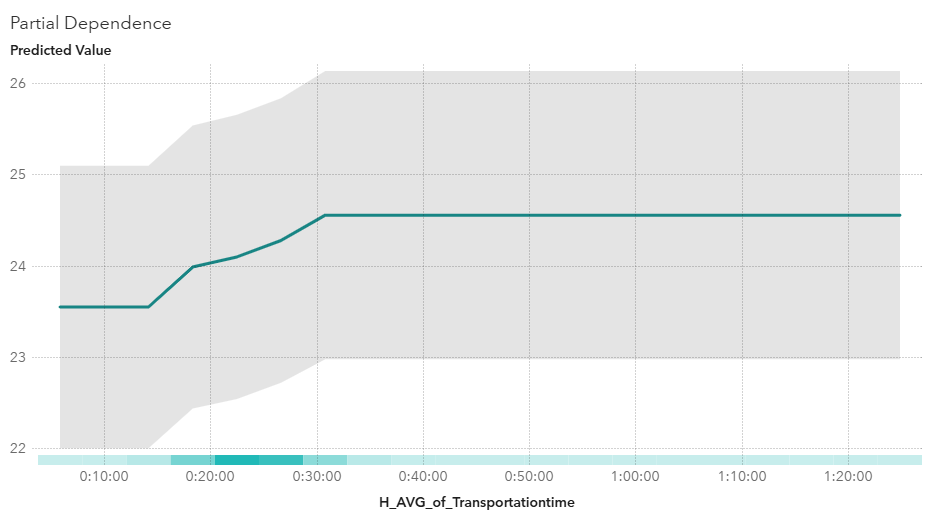


### H_AVG_of_Transportationtime priority level 2


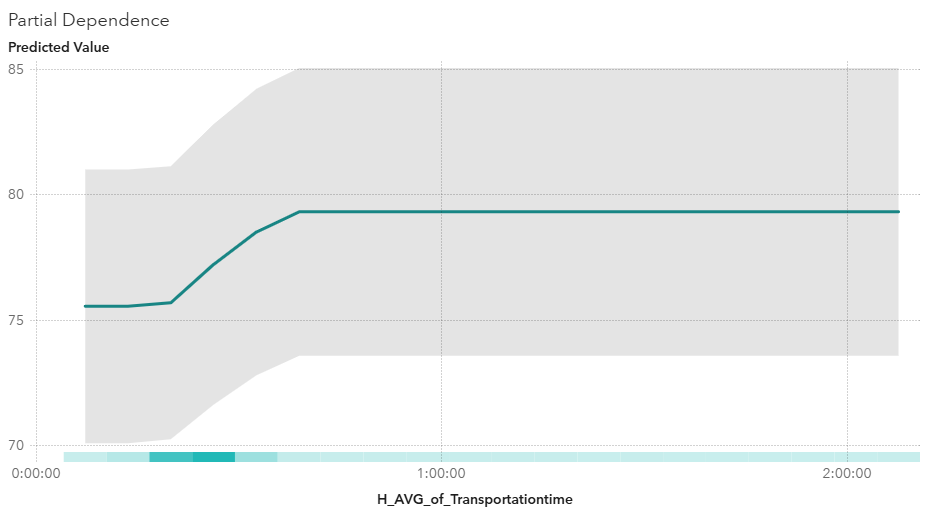


### H_AVG_of_Transportationtime priority level 3


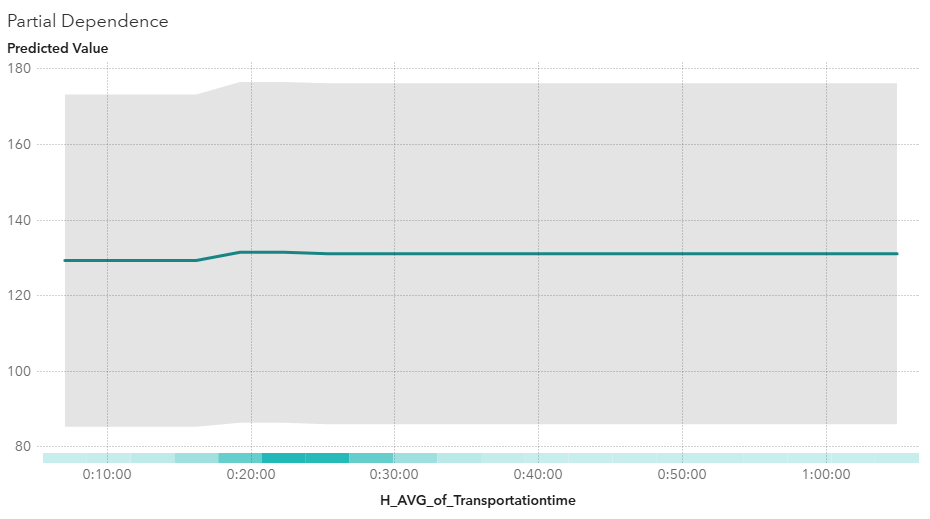


### Amount Precipitation


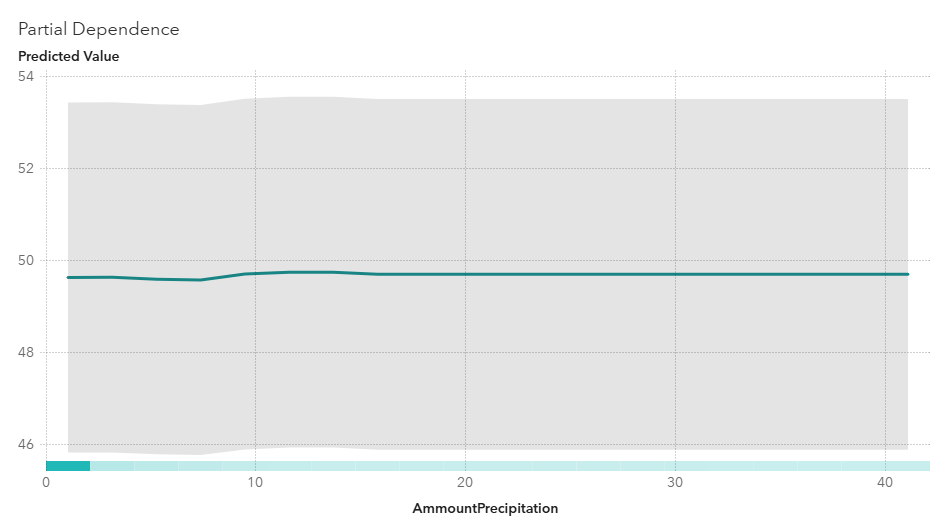


### Amount Precipitation priority level 1


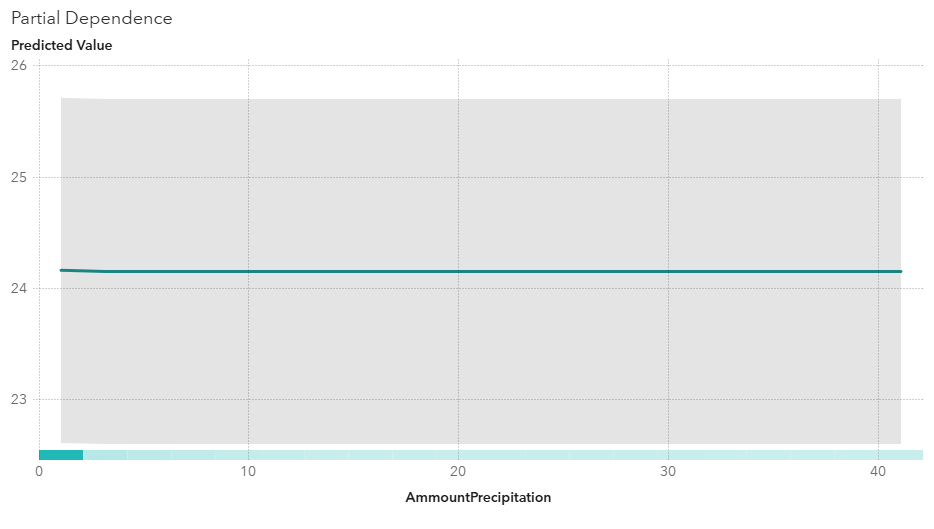


### Amount Precipitation priority level 2


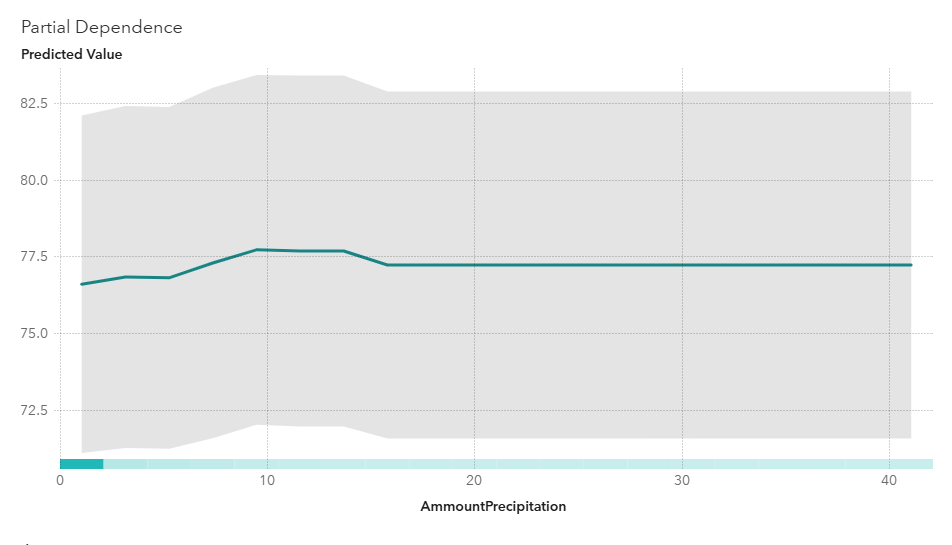


### Amount Precipitation priority level 3


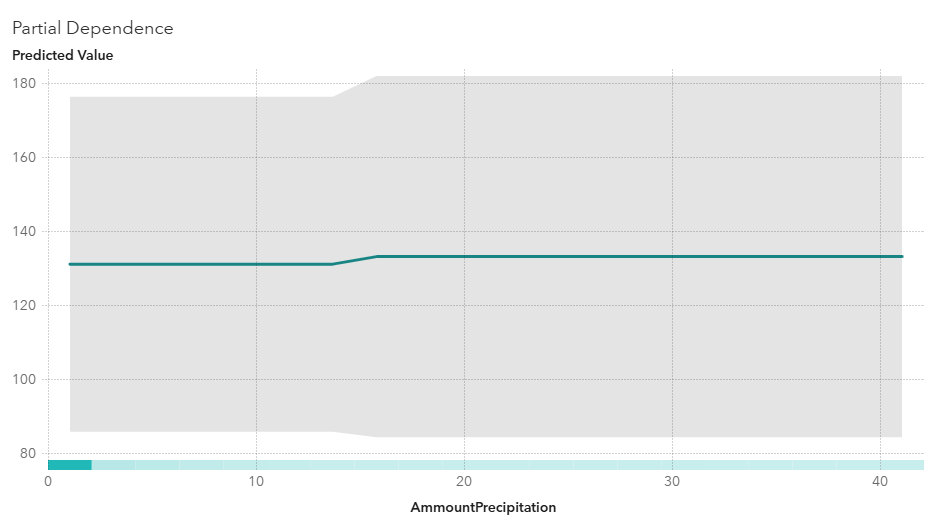


## Model statistics

### Linear regression statistics

#### Dimensions

| Description | Value |
| --- | --- |
| Number of Model Effects | 24 |
| Number of Classification Effects | 9 |
| Number of Columns in X | 212 |
| Rank of Cross-product Matrix | 190 |
| Number of Observations Read | 1 150 473 |
| Number of Observations Used | 520 614 |
| Number of Observations Used for Training | 312 215 |
| Number of Observations Used for Validation | 155 892 |

#### Overall ANOVA

| Source | Deg Freedom | Sum of Squares | Mean Square | F Value | Pr > F | R-Square |
| --- | --- | --- | --- | --- | --- | --- |
| Model | 189 | 410422027.1 | 2171545.117 | 971.9873606 | 0.00000 | 0.370575484 |
| Error | 312025 | 697104090.6 | 2234.128966 |  |  |  |
| Corrected Total | 312214 | 1107526118 |  |  |  |  |

#### Fit statistics

| Statistic | Value |
| --- | --- |
| Mean Square Error | 2234.129 |
| Root MSE | 47.26657 |
| F Value for Model | 971.9874 |
| Pr > F | <0.00001 |
| R-Square | 0.370575 |
| Adjusted R-square | 0.370194 |
| AIC | 2720086 |
| AICC | 2720086 |
| SBC | 2409893 |

#### Parameter estimates

| Parameter | Estimate | Standard Error | t Value | Pr > \|t\| |
| --- | --- | --- | --- | --- |
| Intercept | 55.58595646 | 6.379838631 | 8.712752732 | 0.00000 |
| PrecipitationType Byar av snöblandat regn | -1.853582133 | 1.92532192 | -0.962738809 | 0.33568 |
| PrecipitationType Duggregn | -0.966653118 | 1.71801402 | -0.562657293 | 0.57367 |
| PrecipitationType Kornsnö | -1.692173115 | 1.839855004 | -0.919731778 | 0.35771 |
| PrecipitationType Regn | -1.687834267 | 1.700485508 | -0.992560218 | 0.32093 |
| PrecipitationType Regnskurar | -2.165085247 | 1.747880748 | -1.238691627 | 0.21546 |
| PrecipitationType Småhagel | 0.025657286 | 2.086694691 | 0.012295659 | 0.99019 |
| PrecipitationType Snöblandat regn | -0.820062759 | 1.740108877 | -0.471270947 | 0.63745 |
| PrecipitationType Snöbyar | -1.653108539 | 1.795899991 | -0.920490309 | 0.35732 |
| PrecipitationType Snöfall | -1.596475089 | 1.724098946 | -0.925976489 | 0.35446 |
| PrecipitationType Snöhagel | 0 |  |  |  |
| Priority set by EMCC 1 | -95.5537063 | 0.528447485 | -180.8196822 | 0.00000 |
| Priority set by EMCC 2 | -44.0004219 | 0.522343966 | -84.23648926 | 0.00000 |
| Priority set by EMCC 3 | 0 |  |  |  |
| EMS Station Botkyrka | -5.146526835 | 1.264438377 | -4.070207713 | 0.00005 |
| EMS Station Bro | -0.447282161 | 1.421957818 | -0.314553748 | 0.75310 |
| EMS Station Bromma | 2.523037932 | 1.099970768 | 2.293731802 | 0.02181 |
| EMS Station City | 2.42402668 | 1.10268209 | 2.198300581 | 0.02793 |
| EMS Station Ekerö | -6.740937047 | 1.766756146 | -3.815431498 | 0.00014 |
| EMS Station Farsta | 0.704282734 | 1.141394446 | 0.617037113 | 0.53721 |
| EMS Station Hallstavik | -3.756196927 | 2.016503318 | -1.862727868 | 0.06250 |
| EMS Station Handen | -2.787711374 | 1.238730328 | -2.250458643 | 0.02442 |
| EMS Station Huddinge | -0.551977349 | 1.207196963 | -0.457238848 | 0.64750 |
| EMS Station Hägersten | 1.392935523 | 1.177967329 | 1.182490795 | 0.23701 |
| EMS Station Järfälla | 0.561606747 | 1.198906001 | 0.468432676 | 0.63948 |
| EMS Station Lidingö | -2.749129217 | 1.425417214 | -1.928648813 | 0.05378 |
| EMS Station Lindvreten | -1.673213828 | 1.506963689 | -1.110321264 | 0.26686 |
| EMS Station Märsta | 1.223587775 | 1.324159387 | 0.924048711 | 0.35546 |
| EMS Station Nacka | -2.145945468 | 1.240621043 | -1.729734863 | 0.08368 |
| EMS Station Norrtälje | -1.632900632 | 1.75017268 | -0.932994013 | 0.35082 |
| EMS Station Nynäshamn | -2.338372077 | 1.517797298 | -1.540635288 | 0.12341 |
| EMS Station Reservambulans | -0.928206333 | 1.857356606 | -0.499745891 | 0.61725 |
| EMS Station Skärholmen | -3.043001697 | 1.18531035 | -2.567261559 | 0.01025 |
| EMS Station Sollentuna | 2.496830973 | 1.064231489 | 2.346135215 | 0.01897 |
| EMS Station Solna | 1.362538278 | 1.136917714 | 1.198449334 | 0.23074 |
| EMS Station Södermalm | 0.426519035 | 1.105604217 | 0.385779132 | 0.69966 |
| EMS Station Södertälje | -6.444580716 | 1.332699271 | -4.835735153 | 0.00000 |
| EMS Station Tyresö | 0.652920727 | 1.310000821 | 0.498412457 | 0.61819 |
| EMS Station Täby | -2.556163947 | 1.104799138 | -2.313691113 | 0.02069 |
| EMS Station UpplandsVäsby | 2.004396444 | 1.161539919 | 1.725637157 | 0.08441 |
| EMS Station Vallentuna | -0.760536367 | 1.021466054 | -0.744553736 | 0.45654 |
| EMS Station Vällingby | 2.331392802 | 1.120314847 | 2.081015715 | 0.03743 |
| EMS Station Värmdö | -4.844865619 | 1.407095215 | -3.443168285 | 0.00058 |
| EMS Station Västerhaninge | -2.017050235 | 1.377240353 | -1.464559349 | 0.14304 |
| EMS Station Åkersberga | 0 |  |  |  |
| Locality Botkyrka | 13.53696884 | 4.668128028 | 2.899870946 | 0.00373 |
| Locality Danderyd | 7.728992755 | 4.671820392 | 1.65438568 | 0.09805 |
| Locality Ekerö | 6.469142496 | 4.816160537 | 1.34321571 | 0.17920 |
| Locality Enköping | 8.449002693 | 47.5067025 | 0.177848646 | 0.85884 |
| Locality Gnesta | 56.13467055 | 33.75452207 | 1.663026673 | 0.09631 |
| Locality HAninge | 22.44906499 | 33.75167847 | 0.665124403 | 0.50597 |
| Locality HUDDINGE | 54.51158157 | 33.74682487 | 1.615309937 | 0.10624 |
| Locality HUddinge | 12.28625322 | 47.49653847 | 0.258676813 | 0.79588 |
| Locality Haninge | 11.89944683 | 4.668106391 | 2.549095035 | 0.01080 |
| Locality Huddinge | 12.5503003 | 4.657327076 | 2.694743164 | 0.00704 |
| Locality Håbo | -16.65213044 | 24.09321219 | -0.691154435 | 0.48947 |
| Locality JÄRFÄLLA | 11.90330609 | 47.49498213 | 0.250622393 | 0.80211 |
| Locality JÄrfälla | -6.230265867 | 33.75195245 | -0.184589792 | 0.85355 |
| Locality Järfälla | 10.47724359 | 4.650330369 | 2.253010594 | 0.02426 |
| Locality Jönköping | 0 |  |  |  |
| Locality Knivsta | 21.47006092 | 27.68898571 | 0.775400773 | 0.43810 |
| Locality Lidingö | 7.288079234 | 4.710872529 | 1.547076298 | 0.12185 |
| Locality Linköping | 0 |  |  |  |
| Locality Ludvika | -185.2031782 | 47.56766108 | -3.893468251 | 0.00010 |
| Locality NAcka | 6.469710797 | 18.45959286 | 0.350479604 | 0.72598 |
| Locality NOrrtälje | 0 |  |  |  |
| Locality NYNÄSHAMN | -9.031596461 | 47.50636172 | -0.190113411 | 0.84922 |
| Locality Nacka | 10.91632082 | 4.662358343 | 2.341373188 | 0.01921 |
| Locality Norrtälje | 2.294920497 | 4.828597314 | 0.475276845 | 0.63459 |
| Locality Nykvarn | 8.649707481 | 4.867557026 | 1.777012049 | 0.07557 |
| Locality Nynäshamn | 6.12738162 | 4.750984177 | 1.289707857 | 0.19715 |
| Locality Osby | 0 |  |  |  |
| Locality Riks | -8.590766498 | 17.40516418 | -0.493575723 | 0.62161 |
| Locality SIgtuna | 0 |  |  |  |
| Locality SOLNA | 0 |  |  |  |
| Locality SOlna | 2.39974766 | 27.68100311 | 0.086692944 | 0.93092 |
| Locality STOCKHOLM | 32.26892371 | 17.34258621 | 1.86067541 | 0.06279 |
| Locality STockholm | 17.88308352 | 24.0861257 | 0.742464095 | 0.45781 |
| Locality Salem | 10.04413261 | 4.774758184 | 2.103589799 | 0.03542 |
| Locality Sigtuna | 4.599540851 | 4.666935668 | 0.985559086 | 0.32435 |
| Locality Sollentuna | 7.897323537 | 4.642044653 | 1.701259709 | 0.08890 |
| Locality Solna | 11.60350143 | 4.652275962 | 2.494155877 | 0.01263 |
| Locality Stenungsund | 0 |  |  |  |
| Locality Stockholm | 14.07839858 | 4.63243115 | 3.039095051 | 0.00237 |
| Locality Stockholms län | -25.07291278 | 47.49693078 | -0.527884905 | 0.59758 |
| Locality Strängnäs | -16.04808939 | 27.68951376 | -0.579572813 | 0.56220 |
| Locality Sundbyberg | 15.01005043 | 4.666588738 | 3.21649309 | 0.00130 |
| Locality SÖDERTÄLJE | 2.835635196 | 33.7506336 | 0.084017243 | 0.93304 |
| Locality Södertälje | 16.22005127 | 4.692309401 | 3.456730979 | 0.00055 |
| Locality Trosa | -9.862433559 | 27.69583942 | -0.356098019 | 0.72177 |
| Locality Tyresö | 11.33524157 | 4.685643547 | 2.419142953 | 0.01556 |
| Locality Täby | 6.929928369 | 4.629560884 | 1.496886755 | 0.13442 |
| Locality Upplands Väsby | 8.250610905 | 4.657202888 | 1.771580733 | 0.07647 |
| Locality Upplands väsby | 30.34804613 | 11.08808872 | 2.736995247 | 0.00620 |
| Locality Upplands-Bro | 4.734645545 | 4.749839086 | 0.996801251 | 0.31886 |
| Locality Upplands-bro | 6.939216958 | 4.802383673 | 1.444952638 | 0.14847 |
| Locality Uppsala | 16.85199341 | 24.15608032 | 0.697629466 | 0.48541 |
| Locality VAXHOLM | 0 |  |  |  |
| Locality VAllentuna | 0 |  |  |  |
| Locality VAxholm | 14.84323816 | 47.49502173 | 0.312521979 | 0.75464 |
| Locality Vallentuna | 5.728209913 | 4.65659021 | 1.230129699 | 0.21865 |
| Locality Vaxholm | 0.93006021 | 4.760882956 | 0.195354563 | 0.84512 |
| Locality VÄRMDÖ | 0 |  |  |  |
| Locality Värmdö | 11.13370084 | 4.715420186 | 2.361125924 | 0.01822 |
| Locality botkyrka | 10.81307745 | 7.316524745 | 1.477898023 | 0.13944 |
| Locality danderyd | 8.538032403 | 8.355343783 | 1.021864884 | 0.30685 |
| Locality ekerö | 1.537095393 | 6.896256445 | 0.222888375 | 0.82362 |
| Locality haninge | 8.735739292 | 5.566340742 | 1.56938637 | 0.11656 |
| Locality huddinge | 13.06379858 | 5.363738937 | 2.435576886 | 0.01487 |
| Locality järfälla | 7.540998457 | 7.246035456 | 1.040706812 | 0.29801 |
| Locality lidingö | 9.420915151 | 6.683493894 | 1.409579376 | 0.15866 |
| Locality nacka | 16.43133904 | 6.497645646 | 2.528814272 | 0.01145 |
| Locality norrtälje | 3.239770523 | 6.088191947 | 0.53214001 | 0.59463 |
| Locality nykvarn | 0.601382234 | 18.47323933 | 0.032554238 | 0.97403 |
| Locality nynäshamn | 10.47475495 | 6.532233026 | 1.60354888 | 0.10881 |
| Locality sIGTUNA | 17.39915283 | 47.50014947 | 0.366296802 | 0.71414 |
| Locality sOLNA | 0 |  |  |  |
| Locality sTOCKHOLM | 112.2066359 | 47.50340835 | 2.362075477 | 0.01817 |
| Locality salem | 0.496518331 | 16.43423124 | 0.030212446 | 0.97590 |
| Locality sigtuna | 5.445791446 | 5.995752592 | 0.908274877 | 0.36373 |
| Locality sollentuna | 10.76982764 | 7.73437706 | 1.392462193 | 0.16378 |
| Locality solna | 10.45866425 | 5.684911826 | 1.839723213 | 0.06581 |
| Locality stockholm | 16.12751096 | 4.75962063 | 3.38840261 | 0.00070 |
| Locality sundbyberg | 16.49535467 | 8.640963124 | 1.90897177 | 0.05627 |
| Locality sÖDERTÄLJE | 0 |  |  |  |
| Locality södertälje | 13.90291916 | 5.584167686 | 2.489703021 | 0.01279 |
| Locality tyresö | 10.43746139 | 8.724807535 | 1.196297036 | 0.23158 |
| Locality täby | 12.47483885 | 8.695772539 | 1.43458661 | 0.15141 |
| Locality upplands väsby | 27.30527201 | 11.7908107 | 2.315809549 | 0.02057 |
| Locality upplands-bro | 4.788762424 | 15.6584438 | 0.305826204 | 0.75974 |
| Locality vallentuna | 23.86445677 | 9.659462439 | 2.470578142 | 0.01349 |
| Locality vaxholm | -5.64186195 | 9.771247752 | -0.577394218 | 0.56367 |
| Locality vÄRMDÖ | 19.10458116 | 47.49507333 | 0.402243429 | 0.68751 |
| Locality värmdö | 24.09382154 | 6.475992474 | 3.720483252 | 0.00020 |
| Locality Östersund | 0 |  |  |  |
| Locality Österåker | 5.600832754 | 4.61348202 | 1.214014215 | 0.22474 |
| Locality Östhammar | 0 |  |  |  |
| Locality österåker | 0 |  |  |  |
| Weekday 1 | -0.737178152 | 0.333323229 | -2.211601497 | 0.02699 |
| Weekday 2 | -0.839781262 | 0.318921853 | -2.633188207 | 0.00846 |
| Weekday 3 | -0.54000591 | 0.321039277 | -1.682055587 | 0.09256 |
| Weekday 4 | -0.913061785 | 0.324984507 | -2.809554811 | 0.00496 |
| Weekday 5 | -0.598909431 | 0.321377316 | -1.86357095 | 0.06238 |
| Weekday 6 | -0.282164927 | 0.31826087 | -0.886583786 | 0.37530 |
| Weekday 7 | 0 |  |  |  |
| ResonOfCallToEMCC Abdominal pain | 3.366952865 | 4.022767394 | 0.836974285 | 0.40261 |
| ResonOfCallToEMCC Affected general condition | 9.710823976 | 4.138840802 | 2.346266609 | 0.01896 |
| ResonOfCallToEMCC Allergy | -6.875377733 | 4.082584962 | -1.684074624 | 0.09217 |
| ResonOfCallToEMCC Assault | -13.11526089 | 4.119020482 | -3.184072753 | 0.00145 |
| ResonOfCallToEMCC Bicycle accident | -20.32029119 | 5.552032393 | -3.659973458 | 0.00025 |
| ResonOfCallToEMCC Bite/Sting | -8.086529393 | 5.55815688 | -1.454894054 | 0.14570 |
| ResonOfCallToEMCC Bleeding | 1.070219751 | 4.058826206 | 0.263677156 | 0.79203 |
| ResonOfCallToEMCC Blunt Trauma | -6.47885604 | 11.06898998 | -0.585315919 | 0.55834 |
| ResonOfCallToEMCC Breathing problems | 0.693901804 | 4.018018569 | 0.172697511 | 0.86289 |
| ResonOfCallToEMCC Burn | -11.68298693 | 4.740905752 | -2.46429428 | 0.01373 |
| ResonOfCallToEMCC Cardiac arrest | -8.957415586 | 4.388820534 | -2.040961921 | 0.04126 |
| ResonOfCallToEMCC Catheter discomfort | 12.77848498 | 4.724316474 | 2.704832552 | 0.00683 |
| ResonOfCallToEMCC Changed behavior | -6.74434612 | 4.46347481 | -1.511007994 | 0.13079 |
| ResonOfCallToEMCC Chemical exposure | -10.49737082 | 8.567742625 | -1.22522014 | 0.22049 |
| ResonOfCallToEMCC Chest pain | 0.850796957 | 4.018340592 | 0.211728433 | 0.83232 |
| ResonOfCallToEMCC Childbirth | -2.954208739 | 6.396789141 | -0.461826813 | 0.64421 |
| ResonOfCallToEMCC Complication after surgery | 10.91592042 | 5.161185904 | 2.115002371 | 0.03443 |
| ResonOfCallToEMCC Compromised airway | -13.08664015 | 5.47438525 | -2.390522323 | 0.01683 |
| ResonOfCallToEMCC Convulsion | -8.865758214 | 4.049155171 | -2.189532838 | 0.02856 |
| ResonOfCallToEMCC Diabetes | -0.74277133 | 4.075025299 | -0.182274041 | 0.85537 |
| ResonOfCallToEMCC Diarrhea/vomiting | 8.171849228 | 4.335039179 | 1.88506929 | 0.05942 |
| ResonOfCallToEMCC Dizziness | 8.979353219 | 4.172670697 | 2.151943892 | 0.03140 |
| ResonOfCallToEMCC Drowning accident | -9.610110361 | 6.237953675 | -1.540587004 | 0.12342 |
| ResonOfCallToEMCC Electrical accident | 1.109039485 | 12.14841123 | 0.091290907 | 0.92726 |
| ResonOfCallToEMCC Extremity/Wounds/Minor Trauma | -1.430162415 | 4.021285619 | -0.355648056 | 0.72210 |
| ResonOfCallToEMCC Fainting | -5.975629794 | 4.317012666 | -1.384204832 | 0.16630 |
| ResonOfCallToEMCC Fever/Infection | 5.030595186 | 4.035254975 | 1.246661045 | 0.21252 |
| ResonOfCallToEMCC Headache | 3.530663872 | 4.02979053 | 0.876140793 | 0.38095 |
| ResonOfCallToEMCC High energy trauma | -8.458782961 | 5.216244676 | -1.621623119 | 0.10489 |
| ResonOfCallToEMCC Horse accident | -23.32200214 | 8.655303576 | -2.694533119 | 0.00705 |
| ResonOfCallToEMCC Hypo/Hyperthermia | 5.291167714 | 4.356219089 | 1.214623876 | 0.22451 |
| ResonOfCallToEMCC Immediate danger to life | -11.89589079 | 12.84897348 | -0.925824216 | 0.35454 |
| ResonOfCallToEMCC Low energy trauma | -0.594299721 | 4.050729116 | -0.146714259 | 0.88336 |
| ResonOfCallToEMCC Other | -0.541912366 | 4.016672268 | -0.134915754 | 0.89268 |
| ResonOfCallToEMCC Pain complaints | 1.929235164 | 4.0323465 | 0.478439828 | 0.63234 |
| ResonOfCallToEMCC Penetrating trauma | -10.77281671 | 8.334267475 | -1.292593109 | 0.19615 |
| ResonOfCallToEMCC Person hit by a vehicle | -9.503034115 | 4.442099025 | -2.139311632 | 0.03241 |
| ResonOfCallToEMCC Poisoning | -1.407424008 | 4.049701996 | -0.347537673 | 0.72819 |
| ResonOfCallToEMCC Pregnancy | -0.548178442 | 4.113363612 | -0.133267684 | 0.89398 |
| ResonOfCallToEMCC Psychiatric illness | 1.720088931 | 4.043152826 | 0.425432578 | 0.67052 |
| ResonOfCallToEMCC Rash | -1.775095636 | 7.739881717 | -0.229344026 | 0.81860 |
| ResonOfCallToEMCC Sensation/motor loss | 1.511053698 | 4.412504512 | 0.34244808 | 0.73201 |
| ResonOfCallToEMCC Suspected Stroke/TIA | 1.288574767 | 4.024018319 | 0.3202209 | 0.74880 |
| ResonOfCallToEMCC Swelling | -0.872902552 | 4.721771594 | -0.184867593 | 0.85333 |
| ResonOfCallToEMCC Threats of suicide | -3.856203722 | 4.450603952 | -0.86644504 | 0.38625 |
| ResonOfCallToEMCC Torn body part | 0 |  |  |  |
| ResonOfCallToEMCC Trapped person | 4.993805338 | 27.58522571 | 0.181031882 | 0.85634 |
| ResonOfCallToEMCC Unclear need for care | -3.04425206 | 4.166336276 | -0.730678433 | 0.46498 |
| ResonOfCallToEMCC Unconsciousness/Decreased consciousness | -8.337056943 | 4.037831139 | -2.064736403 | 0.03895 |
| ResonOfCallToEMCC Vision loss | 0 |  |  |  |
| Airtemperature | 0.018580055 | 0.140660998 | 0.13209102 | 0.89491 |
| Airtemperature_max | 0.067167495 | 0.074602494 | 0.900338461 | 0.36794 |
| Airtemperature_min | -0.079985325 | 0.080164638 | -0.997763191 | 0.31839 |
| AmmountPrecipitation | 0.066540052 | 0.019557581 | 3.402263834 | 0.00067 |
| H_AVG_of_DistArrvalkm | -0.662634769 | 0.053616994 | -12.35867056 | 0.00000 |
| H_SUM_of_MissionCanc | 0.016960039 | 0.046335988 | 0.366023035 | 0.71435 |
| H_SUM_of_Missions | 0.82015294 | 0.075819846 | 10.81712743 | 0.00000 |

#### Type III test

| Effect | DF | DenDF | F Value | Pr > F |
| --- | --- | --- | --- | --- |
| PrecipitationType | 9 | 312,025 | 1.673047506 | 0.08938 |
| Priority set by EMCC | 2 | 312,025 | 42724.30275 | 0.00001 |
| EMS Station | 30 | 312,025 | 12.90274397 | 0.00001 |
| Locality | 78 | 312,025 | 7.035194021 | 0.00001 |
| Weekday | 6 | 312,025 | 2.01022987 | 0.06062 |
| ResonOfCallToEMCC | 48 | 312,025 | 34.28546924 | 0.00001 |
| Airtemperature | 1 | 312,025 | 0.017448038 | 0.89491 |
| Airtemperature_max | 1 | 312,025 | 0.810609345 | 0.36794 |
| Airtemperature_min | 1 | 312,025 | 0.995531386 | 0.31839 |
| AmmountPrecipitation | 1 | 312,025 | 11.5753992 | 0.00067 |
| H_AVG_of_DistArrvalkm | 1 | 312,025 | 152.736738 | 0.00001 |
| H_SUM_of_MissionCanc | 1 | 312,025 | 0.133972862 | 0.71435 |
| H_SUM_of_Missions | 1 | 312,025 | 117.0102459 | 0.00001 |
| H_SUM_of_Resources | 1 | 312,025 | 114.0254352 | 0.00001 |
| Hour | 1 | 312,025 | 582.6041912 | 0.00001 |
| Month | 1 | 312,025 | 2.526366008 | 0.11196 |
| H_AVG_of_Call_handelingtime | 0 | 312,025 |  |  |
| H_AVG_of_Deliverytime | 1 | 312,025 | 0.019460678 | 0.88905 |
| H_AVG_of_Drivetime | 0 | 312,025 |  |  |
| H_AVG_of_Onscenetime | 1 | 312,025 | 0.309665147 | 0.57789 |
| H_AVG_of_Responsetime | 0 | 312,025 |  |  |
| H_AVG_of_Transportationtime | 1 | 312,025 | 0.585355135 | 0.44422 |
| TravelDistanceToPatient_ km | 1 | 312,025 | 8564.739672 | 0.00001 |

#### Assessment

| Percentile | Training Observations | Training Predicted Average | Training Observed Average | Validation Observations | Validation Predicted Average | Validation Observed Average |
| --- | --- | --- | --- | --- | --- | --- |
| 5 | 15611 | 129.58 | 161.00 | 7795 | 129.44 | 158.52 |
| 10 | 15611 | 104.44 | 112.43 | 7795 | 104.62 | 113.82 |
| 15 | 15611 | 93.05 | 98.37 | 7795 | 92.96 | 98.94 |
| 20 | 15611 | 85.06 | 85.90 | 7795 | 84.86 | 85.19 |
| 25 | 15611 | 78.79 | 74.66 | 7795 | 78.58 | 73.62 |
| 30 | 15611 | 73.46 | 65.69 | 7795 | 73.32 | 66.25 |
| 35 | 15611 | 68.73 | 58.18 | 7795 | 68.62 | 57.81 |
| 40 | 15611 | 64.28 | 51.68 | 7795 | 64.15 | 51.18 |
| 45 | 15611 | 59.83 | 45.87 | 7795 | 59.61 | 45.02 |
| 50 | 15611 | 54.93 | 40.07 | 7795 | 54.71 | 39.15 |
| 55 | 15611 | 49.14 | 33.36 | 7795 | 48.90 | 33.47 |
| 60 | 15611 | 41.99 | 28.98 | 7795 | 41.83 | 28.87 |
| 65 | 15611 | 34.43 | 25.59 | 7795 | 34.35 | 25.71 |
| 70 | 15611 | 27.82 | 23.87 | 7795 | 27.84 | 24.10 |
| 75 | 15611 | 22.15 | 22.48 | 7795 | 22.17 | 22.28 |
| 80 | 15611 | 17.16 | 21.15 | 7795 | 17.17 | 21.08 |
| 85 | 15611 | 12.42 | 19.79 | 7795 | 12.43 | 19.41 |
| 90 | 15611 | 7.62 | 18.48 | 7795 | 7.55 | 18.49 |
| 95 | 15611 | 2.07 | 17.10 | 7795 | 2.04 | 16.95 |
| 100 | 15606 | -7.71 | 14.58 | 7787 | -7.73 | 14.64 |

#### Assessment statistics

| Partition | ASE | Observed Average | SSE | Observations Used | Unused |
| --- | --- | --- | --- | --- | --- |
| Training | 2,232.7694 | 161.0022 | 697,104,090.5751 | 312,215 | 378,069 |
| Validation | 2,238.6432 | 158.5247 | 348,986,564.4583 | 155,892 | 189,250 |
| Test | 2,217.2440 | 153.9765 | 116,420,833.2910 | 52,507 | 62,540 |

### Gradient Boosting model statistics

#### Overall

##### Variable importance

| Variable | Importance | Standard Deviation |
| --- | --- | --- |
| Priority set by EMCC | 31,267,339.6033 | 40,150,578.2814 |
| H_AVG_of_Responsetime | 9,771,576.8120 | 10,892,769.5457 |
| H_AVG_of_Call_handelingtime | 9,196,869.1868 | 16,797,179.7600 |
| TravelDistanceToPatient_ km | 3,260,152.3358 | 1,161,858.8296 |
| EMS Station | 1,966,382.5414 | 489,350.2304 |
| Hour | 710,004.1145 | 653,923.3281 |
| ResonOfCallToEMCC | 633,218.8821 | 314,031.7585 |
| H_AVG_of_Drivetime | 384,293.6147 | 217,319.3691 |
| Locality | 210,131.0734 | 143,520.6892 |
| H_SUM_of_Missions | 158,529.7463 | 159,017.5768 |
| H_AVG_of_DistArrvalkm | 82,830.4638 | 46,933.3107 |
| Weekday | 81,590.6039 | 47,119.1766 |
| H_AVG_of_Onscenetime | 63,562.1282 | 43,082.0855 |
| H_SUM_of_MissionCanc | 63,403.2513 | 86,107.0433 |
| Airtemperature_min | 56,932.4125 | 80,380.4795 |
| H_AVG_of_Transportationtime | 56,249.6651 | 47,326.3803 |
| Airtemperature_max | 54,403.4899 | 65,220.4884 |
| H_AVG_of_Deliverytime | 39,819.1436 | 34,446.9003 |
| H_SUM_of_Resources | 32,985.0250 | 58,842.9534 |
| Airtemperature | 32,618.3629 | 43,222.9416 |
| Month | 29,226.9991 | 58,924.4273 |
| PrecipitationType | 20,229.5481 | 28,280.0651 |
| AmmountPrecipitation | 17,869.7847 | 25,717.8865 |
| Priority set by EMCC | 31,267,339.6033 | 40,150,578.2814 |

##### Iteration History

| Number of Trees | Average Squared Error | Validation Average Squared Error |
| --- | --- | --- |
| 1 | 2,918.9408 | 2,922.8840 |
| 2 | 2,544.5111 | 2,551.6657 |
| 3 | 2,315.4025 | 2,326.1354 |
| 4 | 2,172.3693 | 2,185.3744 |
| 5 | 2,081.5357 | 2,096.1881 |
| 6 | 2,023.1382 | 2,039.2416 |
| 7 | 1,985.0641 | 2,002.7468 |
| 8 | 1,958.8746 | 1,977.8610 |
| 9 | 1,940.6045 | 1,960.3965 |
| 10 | 1,927.4020 | 1,948.7607 |
| 11 | 1,916.1128 | 1,938.4864 |
| 12 | 1,907.0845 | 1,931.3392 |
| 13 | 1,899.6518 | 1,925.0032 |
| 14 | 1,893.1572 | 1,920.2273 |
| 15 | 1,886.9503 | 1,915.3346 |
| 16 | 1,882.8369 | 1,912.2554 |
| 17 | 1,879.1132 | 1,909.2577 |
| 18 | 1,874.8920 | 1,906.2023 |
| 19 | 1,871.1221 | 1,904.3678 |
| 20 | 1,868.3013 | 1,902.6031 |
| 21 | 1,865.3413 | 1,900.7788 |
| 22 | 1,863.0925 | 1,899.6582 |
| 23 | 1,860.9148 | 1,898.2305 |
| 24 | 1,858.0798 | 1,896.8817 |
| 25 | 1,856.5902 | 1,896.0930 |
| 26 | 1,854.3366 | 1,894.7930 |
| 27 | 1,852.3438 | 1,893.9045 |
| 28 | 1,850.1928 | 1,893.0218 |
| 29 | 1,848.9873 | 1,892.2438 |
| 30 | 1,847.4941 | 1,891.6512 |
| 31 | 1,846.7788 | 1,891.1065 |
| 32 | 1,844.5894 | 1,890.0871 |
| 33 | 1,842.0719 | 1,888.4407 |
| 34 | 1,840.9412 | 1,887.8996 |
| 35 | 1,839.2113 | 1,887.5104 |
| 36 | 1,837.8260 | 1,887.1953 |
| 37 | 1,836.7429 | 1,886.8113 |
| 38 | 1,836.3536 | 1,886.6268 |
| 39 | 1,834.6474 | 1,886.3299 |
| 40 | 1,833.2252 | 1,885.7177 |
| 41 | 1,832.1311 | 1,885.2780 |
| 42 | 1,830.5031 | 1,884.5835 |
| 43 | 1,829.2310 | 1,884.5276 |
| 44 | 1,828.0825 | 1,883.9778 |
| 45 | 1,826.5488 | 1,883.4812 |
| 46 | 1,825.5736 | 1,883.1676 |
| 47 | 1,825.1306 | 1,883.1127 |
| 48 | 1,823.5899 | 1,882.7509 |
| 49 | 1,823.1796 | 1,882.6151 |
| 50 | 1,821.3019 | 1,882.0476 |

##### Assessment

| Percentile | Training Observations | Training Predicted Average | Training Observed Average | Validation Observations | Validation Predicted Average | Validation Observed Average | Test Observations | Test Predicted Average | Test Observed Average |
| --- | --- | --- | --- | --- | --- | --- | --- | --- | --- |
| 5 | 34515 | 168.58 | 172.46 | 17258 | 168.32 | 168.43 | 5753 | 167.95 | 166.66 |
| 10 | 34515 | 117.29 | 117.40 | 17258 | 117.34 | 118.20 | 5753 | 117.16 | 117.64 |
| 15 | 34515 | 97.41 | 97.37 | 17258 | 97.13 | 97.57 | 5753 | 97.26 | 97.17 |
| 20 | 34515 | 84.18 | 83.33 | 17258 | 84.00 | 83.65 | 5753 | 84.18 | 84.61 |
| 25 | 34515 | 73.94 | 73.72 | 17258 | 73.88 | 73.43 | 5753 | 74.01 | 73.36 |
| 30 | 34515 | 65.22 | 64.90 | 17258 | 65.10 | 65.01 | 5753 | 65.37 | 65.50 |
| 35 | 34515 | 57.76 | 57.33 | 17258 | 57.68 | 56.97 | 5753 | 57.88 | 58.36 |
| 40 | 34515 | 51.03 | 50.91 | 17258 | 50.90 | 50.69 | 5753 | 51.16 | 51.25 |
| 45 | 34515 | 44.65 | 44.00 | 17258 | 44.54 | 44.30 | 5753 | 44.87 | 44.06 |
| 50 | 34515 | 38.45 | 38.20 | 17258 | 38.41 | 38.20 | 5753 | 38.73 | 38.45 |
| 55 | 34515 | 33.09 | 32.57 | 17258 | 33.13 | 32.69 | 5753 | 33.40 | 33.35 |
| 60 | 34515 | 29.10 | 28.80 | 17258 | 29.18 | 29.00 | 5753 | 29.34 | 28.59 |
| 65 | 34515 | 26.04 | 25.68 | 17258 | 26.13 | 26.01 | 5753 | 26.19 | 26.12 |
| 70 | 34515 | 23.69 | 23.39 | 17258 | 23.75 | 23.20 | 5753 | 23.80 | 23.67 |
| 75 | 34515 | 21.79 | 21.61 | 17258 | 21.83 | 21.64 | 5753 | 21.85 | 21.18 |
| 80 | 34515 | 20.10 | 19.95 | 17258 | 20.14 | 20.07 | 5753 | 20.14 | 19.98 |
| 85 | 34515 | 18.52 | 18.52 | 17258 | 18.55 | 18.54 | 5753 | 18.55 | 18.52 |
| 90 | 34515 | 16.89 | 16.99 | 17258 | 16.91 | 17.16 | 5753 | 16.91 | 17.16 |
| 95 | 34515 | 14.94 | 15.10 | 17258 | 14.96 | 15.39 | 5753 | 14.96 | 15.37 |
| 100 | 34499 | 11.16 | 11.61 | 17240 | 11.16 | 11.77 | 5740 | 11.15 | 11.64 |

##### Iteration history

| Number of Trees | Average Squared Error | Validation Average Squared Error |
| --- | --- | --- |
| 1 | 632.3327 | 614.8730 |
| 2 | 617.3509 | 599.7475 |
| 3 | 606.6062 | 588.8315 |
| 4 | 597.4099 | 579.4697 |
| 5 | 591.7438 | 573.7693 |
| 6 | 586.4248 | 568.8247 |
| 7 | 582.4223 | 564.7644 |
| 8 | 579.4945 | 562.0843 |
| 9 | 577.0827 | 559.6796 |
| 10 | 575.2927 | 558.0628 |
| 11 | 573.9772 | 557.0894 |
| 12 | 572.3675 | 555.4488 |
| 13 | 571.1792 | 554.5267 |
| 14 | 569.9955 | 553.4408 |
| 15 | 568.9379 | 552.7580 |
| 16 | 568.2188 | 552.3306 |
| 17 | 567.4928 | 551.7022 |
| 18 | 566.7773 | 551.3036 |
| 19 | 566.3905 | 551.0290 |
| 20 | 565.8626 | 550.5703 |
| 21 | 565.4922 | 550.3054 |
| 22 | 565.0103 | 550.2014 |
| 23 | 564.5549 | 549.9834 |
| 24 | 564.2954 | 549.8634 |
| 25 | 563.8866 | 549.7114 |
| 26 | 563.3176 | 549.4014 |
| 27 | 562.9522 | 549.2365 |
| 28 | 562.5537 | 549.3037 |
| 29 | 562.3665 | 549.2667 |
| 30 | 562.1434 | 549.2279 |
| 31 | 561.5567 | 548.9417 |
| 32 | 561.2194 | 548.7721 |
| 33 | 560.9985 | 548.7131 |
| 34 | 560.5846 | 548.5807 |
| 35 | 560.4585 | 548.6029 |
| 36 | 559.9404 | 548.2848 |
| 37 | 559.6707 | 548.2569 |
| 38 | 559.3811 | 548.0875 |
| 39 | 559.0542 | 548.0720 |
| 40 | 558.5171 | 547.7933 |
| 41 | 558.1541 | 547.7452 |
| 42 | 557.7514 | 547.6513 |
| 43 | 557.6167 | 547.6152 |
| 44 | 557.4755 | 547.6138 |
| 45 | 557.1638 | 547.4814 |
| 46 | 556.8889 | 547.4999 |
| 47 | 556.5932 | 547.3945 |
| 48 | 556.4432 | 547.3423 |
| 49 | 556.3871 | 547.3295 |
| 50 | 555.9817 | 547.2797 |

##### Assessment

| Percentile | Training Observations | Training Predicted Average | Training Observed Average | Validation Observations | Validation Predicted Average | Validation Observed Average | Test Observations | Test Predicted Average | Test Observed Average |
| --- | --- | --- | --- | --- | --- | --- | --- | --- | --- |
| 5 | 17392 | 49.40 | 51.82 | 8696 | 49.26 | 49.99 | 2880 | 49.53 | 49.66 |
| 10 | 17392 | 35.83 | 35.66 | 8696 | 35.78 | 36.02 | 2880 | 35.86 | 36.57 |
| 15 | 17392 | 32.07 | 31.90 | 8696 | 32.10 | 31.84 | 2880 | 32.11 | 31.77 |
| 20 | 17392 | 29.55 | 29.09 | 8696 | 29.60 | 29.27 | 2880 | 29.55 | 29.45 |
| 25 | 17392 | 27.49 | 27.48 | 8696 | 27.57 | 27.31 | 2880 | 27.47 | 27.65 |
| 30 | 17392 | 25.84 | 25.88 | 8696 | 25.91 | 26.05 | 2880 | 25.84 | 25.27 |
| 35 | 17392 | 24.54 | 24.21 | 8696 | 24.59 | 24.48 | 2880 | 24.55 | 24.78 |
| 40 | 17392 | 23.45 | 23.30 | 8696 | 23.49 | 22.94 | 2880 | 23.48 | 23.17 |
| 45 | 17392 | 22.49 | 22.44 | 8696 | 22.52 | 22.35 | 2880 | 22.49 | 22.59 |
| 50 | 17392 | 21.59 | 21.35 | 8696 | 21.65 | 21.54 | 2880 | 21.57 | 20.65 |
| 55 | 17392 | 20.78 | 20.51 | 8696 | 20.83 | 20.66 | 2880 | 20.77 | 20.09 |
| 60 | 17392 | 19.99 | 19.66 | 8696 | 20.03 | 19.73 | 2880 | 20.01 | 19.86 |
| 65 | 17392 | 19.22 | 19.07 | 8696 | 19.26 | 19.02 | 2880 | 19.24 | 18.80 |
| 70 | 17392 | 18.45 | 18.40 | 8696 | 18.49 | 18.20 | 2880 | 18.44 | 18.62 |
| 75 | 17392 | 17.66 | 17.66 | 8696 | 17.69 | 17.56 | 2880 | 17.62 | 18.22 |
| 80 | 17392 | 16.82 | 16.59 | 8696 | 16.84 | 17.04 | 2880 | 16.79 | 16.90 |
| 85 | 17392 | 15.88 | 15.82 | 8696 | 15.90 | 15.90 | 2880 | 15.86 | 15.84 |
| 90 | 17392 | 14.77 | 14.78 | 8696 | 14.80 | 14.96 | 2880 | 14.77 | 14.65 |
| 95 | 17392 | 13.20 | 13.18 | 8696 | 13.23 | 13.27 | 2880 | 13.20 | 12.84 |
| 100 | 17388 | 9.94 | 10.14 | 8684 | 9.93 | 10.37 | 2870 | 9.85 | 10.02 |

#### Priority 2

##### Iteration history

| Number of Trees | Average Squared Error | Validation Average Squared Error |
| --- | --- | --- |
| 1 | 3,850.5625 | 3,867.9604 |
| 2 | 3,532.9810 | 3,558.0396 |
| 3 | 3,335.0710 | 3,366.8757 |
| 4 | 3,211.1796 | 3,248.2415 |
| 5 | 3,129.6768 | 3,172.0129 |
| 6 | 3,077.0992 | 3,124.3420 |
| 7 | 3,041.1237 | 3,092.0159 |
| 8 | 3,015.4782 | 3,071.2059 |
| 9 | 2,996.5657 | 3,056.0873 |
| 10 | 2,981.4164 | 3,042.9875 |
| 11 | 2,967.4226 | 3,032.2574 |
| 12 | 2,955.7060 | 3,023.6565 |
| 13 | 2,944.7790 | 3,016.1250 |
| 14 | 2,935.7734 | 3,010.2974 |
| 15 | 2,927.2937 | 3,005.1871 |
| 16 | 2,920.8013 | 3,002.0098 |
| 17 | 2,914.3560 | 2,998.9812 |
| 18 | 2,907.6347 | 2,995.4610 |
| 19 | 2,902.5604 | 2,992.5276 |
| 20 | 2,897.5461 | 2,990.2440 |
| 21 | 2,892.2699 | 2,987.1472 |
| 22 | 2,888.2343 | 2,985.3558 |
| 23 | 2,884.2810 | 2,984.4945 |
| 24 | 2,880.4181 | 2,983.2227 |
| 25 | 2,875.2221 | 2,981.5880 |
| 26 | 2,872.0619 | 2,979.9002 |
| 27 | 2,868.1225 | 2,979.1400 |
| 28 | 2,864.3586 | 2,977.8705 |
| 29 | 2,859.5045 | 2,974.5018 |
| 30 | 2,856.2002 | 2,973.4890 |
| 31 | 2,851.4823 | 2,972.4706 |
| 32 | 2,847.8839 | 2,971.6245 |
| 33 | 2,843.7202 | 2,970.2821 |
| 34 | 2,840.1167 | 2,969.7022 |
| 35 | 2,836.3509 | 2,968.5552 |
| 36 | 2,834.3122 | 2,968.3297 |
| 37 | 2,832.4721 | 2,968.1419 |
| 38 | 2,829.5612 | 2,967.8864 |
| 39 | 2,826.3500 | 2,967.4456 |
| 40 | 2,823.6138 | 2,967.0175 |
| 41 | 2,821.7151 | 2,966.6984 |
| 42 | 2,818.6992 | 2,966.1666 |
| 43 | 2,816.2645 | 2,965.7924 |
| 44 | 2,813.3844 | 2,965.6623 |
| 45 | 2,810.5650 | 2,964.5954 |
| 46 | 2,808.4174 | 2,963.9840 |
| 47 | 2,805.0649 | 2,964.0843 |
| 48 | 2,801.9659 | 2,963.2671 |
| 49 | 2,798.5414 | 2,962.9756 |
| 50 | 2,795.6928 | 2,962.4405 |

##### Assessment

| Percentile | Training Observations | Training Predicted Average | Training Observed Average | Validation Observations | Validation Predicted Average | Validation Observed Average | Test Observations | Test Predicted Average | Test Observed Average |
| --- | --- | --- | --- | --- | --- | --- | --- | --- | --- |
| 5 | 16130 | 182.56 | 190.63 | 8064 | 182.28 | 178.96 | 2704 | 181.50 | 176.57 |
| 10 | 16130 | 137.11 | 138.87 | 8064 | 137.22 | 137.87 | 2704 | 136.71 | 138.53 |
| 15 | 16130 | 119.04 | 119.13 | 8064 | 119.05 | 119.29 | 2704 | 118.67 | 119.74 |
| 20 | 16130 | 107.00 | 107.00 | 8064 | 106.85 | 108.20 | 2704 | 106.62 | 106.15 |
| 25 | 16130 | 97.95 | 98.36 | 8064 | 97.75 | 98.41 | 2704 | 97.69 | 97.92 |
| 30 | 16130 | 90.75 | 89.97 | 8064 | 90.51 | 91.13 | 2704 | 90.51 | 92.46 |
| 35 | 16130 | 84.72 | 83.59 | 8064 | 84.48 | 84.10 | 2704 | 84.49 | 85.63 |
| 40 | 16130 | 79.39 | 78.65 | 8064 | 79.26 | 78.70 | 2704 | 79.24 | 79.24 |
| 45 | 16130 | 74.49 | 73.17 | 8064 | 74.39 | 74.25 | 2704 | 74.40 | 74.78 |
| 50 | 16130 | 69.91 | 69.38 | 8064 | 69.81 | 68.84 | 2704 | 69.86 | 69.55 |
| 55 | 16130 | 65.63 | 65.22 | 8064 | 65.55 | 65.80 | 2704 | 65.56 | 65.64 |
| 60 | 16130 | 61.69 | 60.52 | 8064 | 61.60 | 62.34 | 2704 | 61.62 | 62.64 |
| 65 | 16130 | 58.03 | 57.39 | 8064 | 57.95 | 57.33 | 2704 | 57.81 | 58.47 |
| 70 | 16130 | 54.53 | 53.62 | 8064 | 54.44 | 53.76 | 2704 | 54.33 | 54.08 |
| 75 | 16130 | 51.10 | 50.75 | 8064 | 51.01 | 50.89 | 2704 | 50.94 | 50.89 |
| 80 | 16130 | 47.69 | 47.05 | 8064 | 47.62 | 47.52 | 2704 | 47.57 | 46.72 |
| 85 | 16130 | 44.16 | 43.83 | 8064 | 44.11 | 44.29 | 2704 | 44.08 | 43.93 |
| 90 | 16130 | 40.20 | 39.42 | 8064 | 40.19 | 39.73 | 2704 | 40.15 | 39.40 |
| 95 | 16130 | 35.32 | 34.69 | 8064 | 35.34 | 35.27 | 2704 | 35.22 | 34.76 |
| 100 | 16113 | 25.55 | 25.57 | 8055 | 25.65 | 26.14 | 2702 | 25.86 | 26.16 |

#### Priority 3

##### Iteration history

| Number of Trees | Average Squared Error | Validation Average Squared Error |
| --- | --- | --- |
| 1 | 9,988.4603 | 10,232.2120 |
| 2 | 8,995.7594 | 9,277.2752 |
| 3 | 8,382.6186 | 8,708.2456 |
| 4 | 8,002.4984 | 8,345.7679 |
| 5 | 7,762.2561 | 8,129.6946 |
| 6 | 7,587.5668 | 7,975.4970 |
| 7 | 7,456.7702 | 7,871.7049 |
| 8 | 7,373.4849 | 7,803.6956 |
| 9 | 7,305.7411 | 7,774.9559 |
| 10 | 7,234.0408 | 7,729.5827 |
| 11 | 7,192.1894 | 7,700.4721 |
| 12 | 7,142.8370 | 7,681.3067 |
| 13 | 7,104.8077 | 7,661.7641 |
| 14 | 7,064.4763 | 7,637.2068 |
| 15 | 7,026.9831 | 7,619.2478 |
| 16 | 6,985.1028 | 7,596.3561 |
| 17 | 6,952.1994 | 7,593.2409 |
| 18 | 6,925.4227 | 7,583.5352 |
| 19 | 6,900.9880 | 7,571.3960 |
| 20 | 6,880.4923 | 7,561.7008 |
| 21 | 6,867.0724 | 7,555.4203 |
| 22 | 6,833.6874 | 7,545.5962 |
| 23 | 6,824.2211 | 7,541.6794 |
| 24 | 6,791.5876 | 7,543.7889 |
| 25 | 6,760.7637 | 7,539.2560 |
| 26 | 6,744.0939 | 7,541.3621 |
| 27 | 6,738.1211 | 7,540.3514 |
| 28 | 6,716.7099 | 7,533.1766 |
| 29 | 6,697.8862 | 7,527.1159 |
| 30 | 6,672.5543 | 7,514.8430 |
| 31 | 6,650.5150 | 7,511.5755 |
| 32 | 6,628.0773 | 7,505.3263 |
| 33 | 6,614.4718 | 7,506.5390 |
| 34 | 6,606.8006 | 7,504.6537 |
| 35 | 6,601.0341 | 7,501.9584 |
| 36 | 6,581.8810 | 7,498.9063 |
| 37 | 6,565.8380 | 7,496.5343 |
| 38 | 6,560.7275 | 7,494.0692 |
| 39 | 6,537.4864 | 7,484.2696 |
| 40 | 6,521.5027 | 7,483.4006 |
| 41 | 6,511.5163 | 7,478.7184 |
| 42 | 6,490.1977 | 7,475.4659 |

##### Assessment

| Percentile | Training Observations | Training Predicted Average | Training Observed Average | Validation Observations | Validation Predicted Average | Validation Observed Average | Test Observations | Test Predicted Average | Test Observed Average |
| --- | --- | --- | --- | --- | --- | --- | --- | --- | --- |
| 5 | 994 | 300.73 | 335.84 | 499 | 298.62 | 294.12 | 169 | 297.73 | 278.54 |
| 10 | 994 | 229.72 | 235.93 | 499 | 230.07 | 231.28 | 169 | 229.23 | 233.70 |
| 15 | 994 | 198.11 | 199.30 | 499 | 196.94 | 205.68 | 169 | 197.43 | 174.84 |
| 20 | 994 | 175.91 | 173.48 | 499 | 175.69 | 176.37 | 169 | 174.96 | 170.57 |
| 25 | 994 | 160.32 | 158.44 | 499 | 159.55 | 155.91 | 169 | 157.58 | 150.65 |
| 30 | 994 | 147.56 | 143.24 | 499 | 146.93 | 143.55 | 169 | 144.82 | 150.01 |
| 35 | 994 | 135.98 | 134.12 | 499 | 135.69 | 145.54 | 169 | 134.03 | 134.71 |
| 40 | 994 | 126.04 | 121.22 | 499 | 125.60 | 123.01 | 169 | 124.46 | 110.79 |
| 45 | 994 | 117.19 | 113.84 | 499 | 116.39 | 116.82 | 169 | 115.96 | 119.80 |
| 50 | 994 | 108.61 | 107.81 | 499 | 108.30 | 106.52 | 169 | 108.09 | 93.34 |
| 55 | 994 | 100.98 | 96.52 | 499 | 101.03 | 104.35 | 169 | 100.19 | 94.63 |
| 60 | 994 | 93.82 | 87.09 | 499 | 93.94 | 91.35 | 169 | 92.51 | 97.52 |
| 65 | 994 | 86.92 | 87.85 | 499 | 86.99 | 89.56 | 169 | 85.48 | 82.11 |
| 70 | 994 | 80.19 | 76.83 | 499 | 80.44 | 79.76 | 169 | 79.22 | 81.98 |
| 75 | 994 | 73.88 | 71.74 | 499 | 73.97 | 76.95 | 169 | 72.67 | 64.57 |
| 80 | 994 | 67.89 | 65.54 | 499 | 67.69 | 66.68 | 169 | 66.92 | 73.56 |
| 85 | 994 | 62.03 | 61.33 | 499 | 61.53 | 60.04 | 169 | 61.26 | 63.26 |
| 90 | 994 | 55.33 | 53.19 | 499 | 54.67 | 53.80 | 169 | 54.82 | 52.46 |
| 95 | 994 | 46.51 | 44.88 | 499 | 45.74 | 47.58 | 169 | 46.02 | 46.27 |
| 100 | 979 | 29.73 | 29.35 | 482 | 29.71 | 30.42 | 168 | 30.86 | 29.97 |

## Missing data

| **incidentid_lopnr** | **Frequency** | **Percent** |
| --- | --- | --- |
| **Non-missing** | 1150474 | 100 |
|  |  |  |
| **taskid_lopnr** | **Frequency** | **Percent** |
| **Non-missing** | 1150474 | 100 |
|  |  |  |
| **rakelid_lopnr** | **Frequency** | **Percent** |
| **Non-missing** | 1150474 | 100 |
|  |  |  |
| **Priority set by EMCC** | **Frequency** | **Percent** |
| **Non-missing** | 1150474 | 100 |
|  |  |  |
| **Station** | | |
| **Station** | **Frequency** | **Percent** |
| **Non-missing** | 1150474 | 100 |
|  |  |  |
| **Ort** | | |
| **KOMMUN** | **Frequency** | **Percent** |
|  | 139 | 0.01 |
| **Non-missing** | 1150335 | 99.99 |
|  |  |  |
| **Receved emergencycall UTC** | **Frequency** | **Percent** |
| **Non-missing** | 1150474 | 100 |
|  |  |  |
| **Year** | **Frequency** | **Percent** |
| **Non-missing** | 1150474 | 100 |
|  |  |  |
| **Month** | **Frequency** | **Percent** |
| **Non-missing** | 1150474 | 100 |
|  |  |  |
| **Weekday** | **Frequency** | **Percent** |
| **Non-missing** | 1150474 | 100 |
|  |  |  |
| **Hour** | **Frequency** | **Percent** |
| **Non-missing** | 1150474 | 100 |
|  |  |  |
| **TravelDistanceToPatient_ km** | **Frequency** | **Percent** |
| **.** | 4427 | 0.38 |
| **Non-missing** | 1146047 | 99.62 |
|  |  |  |
| **Call_handelingtime** | **Frequency** | **Percent** |
| **Non-missing** | 1150474 | 100 |
|  |  |  |
| **Drivetime** | **Frequency** | **Percent** |
| **Non-missing** | 1150474 | 100 |
|  |  |  |
| **Date** | **Frequency** | **Percent** |
| **Non-missing** | 1150474 | 100 |
|  |  |  |
| **MissionCancelled** | **Frequency** | **Percent** |
| **Non-missing** | 1150474 | 100 |
|  |  |  |
| **Onscenetime** | **Frequency** | **Percent** |
| **.** | 220418 | 19.16 |
| **Non-missing** | 930056 | 80.84 |
|  |  |  |
| **Transportationtime** | **Frequency** | **Percent** |
| **.** | 228146 | 19.83 |
| **Non-missing** | 922328 | 80.17 |
|  |  |  |
| **Deliverytime** | **Frequency** | **Percent** |
| **.** | 319598 | 27.78 |
| **Non-missing** | 830876 | 72.22 |
|  |  |  |
| **Airtemperature_min** | **Frequency** | **Percent** |
| **Non-missing** | 1150474 | 100 |
|  |  |  |
| **Airtemperature_max** | **Frequency** | **Percent** |
| **Non-missing** | 1150474 | 100 |
|  |  |  |
| **AmmountPrecipitation** | **Frequency** | **Percent** |
| **Non-missing** | 1150474 | 100 |
|  |  |  |
| **Airtemperature** | **Frequency** | **Percent** |
| **Non-missing** | 1150474 | 100 |
|  |  |  |
| **PrecipitationType** | **Frequency** | **Percent** |
|  | 627881 | 54.58 |
| **Non-missing** | 522593 | 45.42 |
|  |  |  |
| **H_SUM_of_Resources** | **Frequency** | **Percent** |
| **Non-missing** | 1150474 | 100 |
|  |  |  |
| **H_AVG_of_Onscenetime** | **Frequency** | **Percent** |
| **.** | 8 | 0 |
| **Non-missing** | 1150466 | 100 |
|  |  |  |
| **H_AVG_of_Transportationtime** | **Frequency** | **Percent** |
| **.** | 15 | 0 |
| **Non-missing** | 1150459 | 100 |
|  |  |  |
| **H_AVG_of_Deliverytime** | **Frequency** | **Percent** |
| **.** | 26 | 0 |
| **Non-missing** | 1150448 | 100 |
|  |  |  |
| **H_AVG_of_Responsetime** | **Frequency** | **Percent** |
| **Non-missing** | 1150474 | 100 |
|  |  |  |
| **H_AVG_of_Call_handelingtime** | **Frequency** | **Percent** |
| **Non-missing** | 1150474 | 100 |
|  |  |  |
| **H_AVG_of_Drivetime** | **Frequency** | **Percent** |
| **Non-missing** | 1150474 | 100 |
|  |  |  |
| **H_AVG_of_DistArrvalkm** | **Frequency** | **Percent** |
| **Non-missing** | 1150474 | 100 |
|  |  |  |
| **H_SUM_of_Missions** | **Frequency** | **Percent** |
| **Non-missing** | 1150474 | 100 |
|  |  |  |
| **H_SUM_of_MissionCanc** | **Frequency** | **Percent** |
| **Non-missing** | 1150474 | 100 |
|  |  |  |
| **ResponseTime** | **Frequency** | **Percent** |
| **Non-missing** | 1150474 | 100 |

## SAS code

### SAS code from feature engineering in SAS Enterprise Guide 8.2

/* ----------------------------------------

Kod exporterad från SAS Enterprise Guide

DATUM: 07 January 2025 TID: 09:07:11

PROJEKT: Study I Revision 2.2

PROJEKTSÖKVÄG: C:\Users\4khw\OneDrive - Karolinska Institutet\General\Studie I\Revision II\Analyses\Study I Revision 2.2.egp

---------------------------------------- */

/* Bibliotekstilldelning för SASAppUser.BI_KFA */

Libname BI_KFA META LIBURI="SASLibrary?*[@Name='BI_KFA'][DeployedComponents/ServerContext[@Name='SASAppUser']]" METAOUT=DATA;

/* Bibliotekstilldelning för SASAppUser.BI_KFA */

Libname BI_KFA META LIBURI="SASLibrary?*[@Name='BI_KFA'][DeployedComponents/ServerContext[@Name='SASAppUser']]" METAOUT=DATA;

/* Bibliotekstilldelning för SASAppUser.BI_KFA */

Libname BI_KFA META LIBURI="SASLibrary?*[@Name='BI_KFA'][DeployedComponents/ServerContext[@Name='SASAppUser']]" METAOUT=DATA;

/* Bibliotekstilldelning för SASAppUser.VA_VIYA */

Libname VA_VIYA META LIBURI="SASLibrary?*[@Name='VA_VIYA'][DeployedComponents/ServerContext[@Name='SASAppUser']]";

/* Bibliotekstilldelning för SASAppUser.J */

Libname J V9 '/home2/S4KHW' ;

/* Conditionally delete set of tables or views, if they exists */

/* If the member does not exist, then no action is performed */

%macro _eg_conditional_dropds /parmbuff;

%local num;

%local stepneeded;

%local stepstarted;

%local dsname;

%local name;

%let num=1;

/* flags to determine whether a PROC SQL step is needed */

/* or even started yet */

%let stepneeded=0;

%let stepstarted=0;

%let dsname= %qscan(&syspbuff,&num,',()');

%do %while(&dsname ne);

%let name = %sysfunc(left(&dsname));

%if %qsysfunc(exist(&name)) %then %do;

%let stepneeded=1;

%if (&stepstarted eq 0) %then %do;

proc sql;

%let stepstarted=1;

%end;

drop table &name;

%end;

%if %sysfunc(exist(&name,view)) %then %do;

%let stepneeded=1;

%if (&stepstarted eq 0) %then %do;

proc sql;

%let stepstarted=1;

%end;

drop view &name;

%end;

%let num=%eval(&num+1);

%let dsname=%qscan(&syspbuff,&num,',()');

%end;

%if &stepstarted %then %do;

quit;

%end;

%mend _eg_conditional_dropds;

/* Build where clauses from stored process parameters */

%macro _eg_WhereParam( COLUMN, PARM, OPERATOR, TYPE=S, MATCHALL=_ALL_VALUES_, MATCHALL_CLAUSE=1, MAX= , IS_EXPLICIT=0, MATCH_CASE=1);

%local q1 q2 sq1 sq2;

%local isEmpty;

%local isEqual isNotEqual;

%local isIn isNotIn;

%local isString;

%local isBetween;

%let isEqual = ("%QUPCASE(&OPERATOR)" = "EQ" OR "&OPERATOR" = "=");

%let isNotEqual = ("%QUPCASE(&OPERATOR)" = "NE" OR "&OPERATOR" = "<>");

%let isIn = ("%QUPCASE(&OPERATOR)" = "IN");

%let isNotIn = ("%QUPCASE(&OPERATOR)" = "NOT IN");

%let isString = (%QUPCASE(&TYPE) eq S or %QUPCASE(&TYPE) eq STRING );

%if &isString %then

%do;

%if "&MATCH_CASE" eq "0" %then %do;

%let COLUMN = %str(UPPER%(&COLUMN%));

%end;

%let q1=%str(%");

%let q2=%str(%");

%let sq1=%str(%');

%let sq2=%str(%');

%end;

%else %if %QUPCASE(&TYPE) eq D or %QUPCASE(&TYPE) eq DATE %then

%do;

%let q1=%str(%");

%let q2=%str(%"d);

%let sq1=%str(%');

%let sq2=%str(%');

%end;

%else %if %QUPCASE(&TYPE) eq T or %QUPCASE(&TYPE) eq TIME %then

%do;

%let q1=%str(%");

%let q2=%str(%"t);

%let sq1=%str(%');

%let sq2=%str(%');

%end;

%else %if %QUPCASE(&TYPE) eq DT or %QUPCASE(&TYPE) eq DATETIME %then

%do;

%let q1=%str(%");

%let q2=%str(%"dt);

%let sq1=%str(%');

%let sq2=%str(%');

%end;

%else

%do;

%let q1=;

%let q2=;

%let sq1=;

%let sq2=;

%end;

%if "&PARM" = "" %then %let PARM=&COLUMN;

%let isBetween = ("%QUPCASE(&OPERATOR)"="BETWEEN" or "%QUPCASE(&OPERATOR)"="NOT BETWEEN");

%if "&MAX" = "" %then %do;

%let MAX = &parm._MAX;

%if &isBetween %then %let PARM = &parm._MIN;

%end;

%if not %symexist(&PARM) or (&isBetween and not %symexist(&MAX)) %then %do;

%if &IS_EXPLICIT=0 %then %do;

not &MATCHALL_CLAUSE

%end;

%else %do;

not 1=1

%end;

%end;

%else %if "%qupcase(&&&PARM)" = "%qupcase(&MATCHALL)" %then %do;

%if &IS_EXPLICIT=0 %then %do;

&MATCHALL_CLAUSE

%end;

%else %do;

1=1

%end;

%end;

%else %if (not %symexist(&PARM._count)) or &isBetween %then %do;

%let isEmpty = ("&&&PARM" = "");

%if (&isEqual AND &isEmpty AND &isString) %then

&COLUMN is null;

%else %if (&isNotEqual AND &isEmpty AND &isString) %then

&COLUMN is not null;

%else %do;

%if &IS_EXPLICIT=0 %then %do;

&COLUMN &OPERATOR

%if "&MATCH_CASE" eq "0" %then %do;

%unquote(&q1)%QUPCASE(&&&PARM)%unquote(&q2)

%end;

%else %do;

%unquote(&q1)&&&PARM%unquote(&q2)

%end;

%end;

%else %do;

&COLUMN &OPERATOR

%if "&MATCH_CASE" eq "0" %then %do;

%unquote(%nrstr(&sq1))%QUPCASE(&&&PARM)%unquote(%nrstr(&sq2))

%end;

%else %do;

%unquote(%nrstr(&sq1))&&&PARM%unquote(%nrstr(&sq2))

%end;

%end;

%if &isBetween %then

AND %unquote(&q1)&&&MAX%unquote(&q2);

%end;

%end;

%else

%do;

%local emptyList;

%let emptyList = %symexist(&PARM._count);

%if &emptyList %then %let emptyList = &&&PARM._count = 0;

%if (&emptyList) %then

%do;

%if (&isNotin) %then

1;

%else

0;

%end;

%else %if (&&&PARM._count = 1) %then

%do;

%let isEmpty = ("&&&PARM" = "");

%if (&isIn AND &isEmpty AND &isString) %then

&COLUMN is null;

%else %if (&isNotin AND &isEmpty AND &isString) %then

&COLUMN is not null;

%else %do;

%if &IS_EXPLICIT=0 %then %do;

%if "&MATCH_CASE" eq "0" %then %do;

&COLUMN &OPERATOR (%unquote(&q1)%QUPCASE(&&&PARM)%unquote(&q2))

%end;

%else %do;

&COLUMN &OPERATOR (%unquote(&q1)&&&PARM%unquote(&q2))

%end;

%end;

%else %do;

&COLUMN &OPERATOR (

%if "&MATCH_CASE" eq "0" %then %do;

%unquote(%nrstr(&sq1))%QUPCASE(&&&PARM)%unquote(%nrstr(&sq2)))

%end;

%else %do;

%unquote(%nrstr(&sq1))&&&PARM%unquote(%nrstr(&sq2)))

%end;

%end;

%end;

%end;

%else

%do;

%local addIsNull addIsNotNull addComma;

%let addIsNull = %eval(0);

%let addIsNotNull = %eval(0);

%let addComma = %eval(0);

(&COLUMN &OPERATOR (

%do i=1 %to &&&PARM._count;

%let isEmpty = ("&&&PARM&i" = "");

%if (&isString AND &isEmpty AND (&isIn OR &isNotIn)) %then

%do;

%if (&isIn) %then %let addIsNull = 1;

%else %let addIsNotNull = 1;

%end;

%else

%do;

%if &addComma %then %do;,%end;

%if &IS_EXPLICIT=0 %then %do;

%if "&MATCH_CASE" eq "0" %then %do;

%unquote(&q1)%QUPCASE(&&&PARM&i)%unquote(&q2)

%end;

%else %do;

%unquote(&q1)&&&PARM&i%unquote(&q2)

%end;

%end;

%else %do;

%if "&MATCH_CASE" eq "0" %then %do;

%unquote(%nrstr(&sq1))%QUPCASE(&&&PARM&i)%unquote(%nrstr(&sq2))

%end;

%else %do;

%unquote(%nrstr(&sq1))&&&PARM&i%unquote(%nrstr(&sq2))

%end;

%end;

%let addComma = %eval(1);

%end;

%end;)

%if &addIsNull %then OR &COLUMN is null;

%else %if &addIsNotNull %then AND &COLUMN is not null;

%do;)

%end;

%end;

%end;

%mend _eg_WhereParam;

/* ---------------------------------- */

/* MACRO: enterpriseguide */

/* PURPOSE: define a macro variable */

/* that contains the file system */

/* path of the WORK library on the */

/* server. Note that different */

/* logic is needed depending on the */

/* server type. */

/* ---------------------------------- */

%macro enterpriseguide;

%global sasworklocation;

%local tempdsn unique_dsn path;

%if &sysscp=OS %then %do; /* MVS Server */

%if %sysfunc(getoption(filesystem))=MVS %then %do;

/* By default, physical file name will be considered a classic MVS data set. */

/* Construct dsn that will be unique for each concurrent session under a particular account: */

filename egtemp '&egtemp' disp=(new,delete); /* create a temporary data set */

%let tempdsn=%sysfunc(pathname(egtemp)); /* get dsn */

filename egtemp clear; /* get rid of data set - we only wanted its name */

%let unique_dsn=".EGTEMP.%substr(&tempdsn, 1, 16).PDSE";

filename egtmpdir &unique_dsn

disp=(new,delete,delete) space=(cyl,(5,5,50))

dsorg=po dsntype=library recfm=vb

lrecl=8000 blksize=8004 ;

options fileext=ignore ;

%end;

%else %do;

/*

By default, physical file name will be considered an HFS

(hierarchical file system) file.

*/

%if "%sysfunc(getoption(filetempdir))"="" %then %do;

filename egtmpdir '/tmp';

%end;

%else %do;

filename egtmpdir "%sysfunc(getoption(filetempdir))";

%end;

%end;

%let path=%sysfunc(pathname(egtmpdir));

%let sasworklocation=%sysfunc(quote(&path));

%end; /* MVS Server */

%else %do;

%let sasworklocation = "%sysfunc(getoption(work))/";

%end;

%if &sysscp=VMS_AXP %then %do; /* Alpha VMS server */

%let sasworklocation = "%sysfunc(getoption(work))";

%end;

%if &sysscp=CMS %then %do;

%let path = %sysfunc(getoption(work));

%let sasworklocation = "%substr(&path, %index(&path,%str( )))";

%end;

%mend enterpriseguide;

%enterpriseguide

/* save the current settings of XPIXELS and YPIXELS */

/* so that they can be restored later */

%macro _sas_pushchartsize(new_xsize, new_ysize);

%global _savedxpixels _savedypixels;

options nonotes;

proc sql noprint;

select setting into :_savedxpixels

from sashelp.vgopt

where optname eq "XPIXELS";

select setting into :_savedypixels

from sashelp.vgopt

where optname eq "YPIXELS";

quit;

options notes;

GOPTIONS XPIXELS=&new_xsize YPIXELS=&new_ysize;

%mend _sas_pushchartsize;

/* restore the previous values for XPIXELS and YPIXELS */

%macro _sas_popchartsize;

%if %symexist(_savedxpixels) %then %do;

GOPTIONS XPIXELS=&_savedxpixels YPIXELS=&_savedypixels;

%symdel _savedxpixels / nowarn;

%symdel _savedypixels / nowarn;

%end;

%mend _sas_popchartsize;

ODS PROCTITLE;

OPTIONS DEV=SVG;

GOPTIONS XPIXELS=0 YPIXELS=0;

%macro HTML5AccessibleGraphSupported;

%if %_SAS_VERCOMP_FV(9,4,4, 0,0,0) >= 0 %then ACCESSIBLE_GRAPH;

%mend;

FILENAME EGHTMLX TEMP;

ODS HTML5(ID=EGHTMLX) FILE=EGHTMLX

OPTIONS(BITMAP_MODE='INLINE')

%HTML5AccessibleGraphSupported

ENCODING='utf-8'

STYLE=HTMLBlue

NOGTITLE

NOGFOOTNOTE

GPATH=&sasworklocation

;

/* START PÅ NOD: Frågebyggare-Grundläggande filter */

%LET _CLIENTTASKLABEL='Frågebyggare-Grundläggande filter';

%LET _CLIENTPROCESSFLOWNAME='Processflöde';

%LET _CLIENTPROJECTPATH='C:\Users\4khw\OneDrive - Karolinska Institutet\General\Studie I\Revision II\Analyses\Study I Revision 2.2.egp';

%LET _CLIENTPROJECTPATHHOST='RSTPC00020406';

%LET _CLIENTPROJECTNAME='Study I Revision 2.2.egp';

%_eg_conditional_dropds(WORK.QUERY_FOR_DYNEMSALL_TASKLASTVALU);

PROC SQL;

CREATE TABLE WORK.QUERY_FOR_DYNEMSALL_TASKLASTVALU AS

SELECT DISTINCT /* Responsetime */

(((INPUT(t1.MOTTAGEN,anydtdtm.))-(input(t1.LARMTIDAMBULANS,

anydtdtm.)))+(t2.FirstFramme-t2.FirstMottaget)) FORMAT=TIME8. AS Responsetime,

t1.incidentid_lopnr,

t1.taskid_lopnr,

t1.rakelid_lopnr,

/* Receved emergencycall UTC */

(input(t1.LARMTIDAMBULANS, anydtdtm.)) FORMAT=SVEDFDT19. AS 'Receved emergencycall UTC'n,

/* Year */

(YEAR(DATEPART((input(t1.LARMTIDAMBULANS, anydtdtm.))))) FORMAT=NUMX12. AS Year,

/* Hour */

(HOUR((input(t1.LARMTIDAMBULANS, anydtdtm.)))) FORMAT=NUMX12. AS Hour,

t1.PRIORITETUT LABEL="Priority set by dispatch" AS 'Priority set by dispatch'n,

/* DistancetoArrival km */

(t1.DFramme/1000) FORMAT=COMMAX6.3 AS 'DistancetoArrival km'n,

/* Call_handelingtime */

((INPUT(t1.MOTTAGEN,anydtdtm.))-(input(t1.LARMTIDAMBULANS, anydtdtm.))) FORMAT=TIME8. AS Call_handelingtime,

/* Drivetime */

(t2.FirstFramme-t2.FirstMottaget) FORMAT=TIME8. AS Drivetime,

/* Date */

(DATEPART(input(t1.LARMTIDAMBULANS, anydtdtm.))) FORMAT=AFRDFDE7. AS Date,

/* MissionCancelled */

(COUNT(DISTINCT t1.Avbrutet_uppdrag)) AS MissionCancelled,

t1.RakelIndex1,

t1.PRIORITETUT,

/* Onscenetime */

(t1.Lastat-t1.Framme) FORMAT=TIME8. AS Onscenetime,

/* Transportationtime */

(t1.Snart_klar-t1.Lastat) FORMAT=TIME8. AS Transportationtime,

/* Deliverytime */

(t1.Klar_uppdrag-t1.Snart_klar) FORMAT=TIME8. AS Deliverytime,

/* Month */

(MONTH(DATEPART((input(t1.LARMTIDAMBULANS, anydtdtm.))))) FORMAT=NUMX12. AS Month,

/* Weekday */

(WEEKDAY(DATEPART((input(t1.LARMTIDAMBULANS, anydtdtm.))))) AS Weekday,

t1.Station,

/* ResonOfCallToEMCC */

(CASE

WHEN 'Allergi' = t1.RakelIndex1 THEN 'Allergy'

WHEN 'Allergi Barn 1-6 år' = t1.RakelIndex1 THEN 'Allergy'

WHEN 'Allergi Barn 7-15 år' = t1.RakelIndex1 THEN 'Allergy'

WHEN 'Allergi Spädbarn' = t1.RakelIndex1 THEN 'Allergy'

WHEN 'Allergi Vuxen' = t1.RakelIndex1 THEN 'Allergy'

WHEN 'Andningsbesvär' = t1.RakelIndex1 THEN 'Breathing problems'

WHEN 'Andningsbesvär Barn 1-6 år' = t1.RakelIndex1 THEN 'Breathing problems'

WHEN 'Andningsbesvär Barn 7-15 år' = t1.RakelIndex1 THEN 'Breathing problems'

WHEN 'Andningsbesvär Spädbarn' = t1.RakelIndex1 THEN 'Breathing problems'

WHEN 'Andningsbesvär Vuxen' = t1.RakelIndex1 THEN 'Breathing problems'

WHEN 'Andningssvårigheter' = t1.RakelIndex1 THEN 'Breathing problems'

WHEN 'Avsliten kroppsdel Vuxen' = t1.RakelIndex1 THEN 'Torn body part'

WHEN 'Bett/Stick Barn 1-6 år' = t1.RakelIndex1 THEN 'Bite/Sting'

WHEN 'Bett/Stick Vuxen' = t1.RakelIndex1 THEN 'Bite/Sting'

WHEN 'Blödning Ansikte' = t1.RakelIndex1 THEN 'Bleeding'

WHEN 'Blödning Ansikte Vuxen' = t1.RakelIndex1 THEN 'Bleeding'

WHEN 'Blödning Ansikte Vuxen' = t1.RakelIndex1 THEN 'Bleeding'

WHEN 'Blödning Bål Vuxen' = t1.RakelIndex1 THEN 'Bleeding'

WHEN 'Blödning Bål Vuxen' = t1.RakelIndex1 THEN 'Bleeding'

WHEN 'Blödning Barn 1-6 år' = t1.RakelIndex1 THEN 'Bleeding'

WHEN 'Blödning Barn 1-6 år' = t1.RakelIndex1 THEN 'Bleeding'

WHEN 'Blödning Barn 7-15 år' = t1.RakelIndex1 THEN 'Bleeding'

WHEN 'Blödning Barn 7-15 år' = t1.RakelIndex1 THEN 'Bleeding'

WHEN 'Blödning Barn 7-15år' = t1.RakelIndex1 THEN 'Bleeding'

WHEN 'Blödning Barn 7-15år' = t1.RakelIndex1 THEN 'Bleeding'

WHEN 'Blödning Extremitet' = t1.RakelIndex1 THEN 'Bleeding'

WHEN 'Blödning Extremitet' = t1.RakelIndex1 THEN 'Bleeding'

WHEN 'Blödning Extremitet Vuxen' = t1.RakelIndex1 THEN 'Bleeding'

WHEN 'Blödning Extremitet Vuxen' = t1.RakelIndex1 THEN 'Bleeding'

WHEN 'Blödning Mun' = t1.RakelIndex1 THEN 'Bleeding'

WHEN 'Blödning Mun' = t1.RakelIndex1 THEN 'Bleeding'

WHEN 'Blödning Mun Vuxen' = t1.RakelIndex1 THEN 'Bleeding'

WHEN 'Blödning Mun Vuxen' = t1.RakelIndex1 THEN 'Bleeding'

WHEN 'Blödning Näsa' = t1.RakelIndex1 THEN 'Bleeding'

WHEN 'Blödning Näsa' = t1.RakelIndex1 THEN 'Bleeding'

WHEN 'Blödning Näsa Vuxen' = t1.RakelIndex1 THEN 'Bleeding'

WHEN 'Blödning Näsa Vuxen' = t1.RakelIndex1 THEN 'Bleeding'

WHEN 'Blödning Rektal' = t1.RakelIndex1 THEN 'Bleeding'

WHEN 'Blödning Rektal' = t1.RakelIndex1 THEN 'Bleeding'

WHEN 'Blödning Rektal Vuxen' = t1.RakelIndex1 THEN 'Bleeding'

WHEN 'Blödning Rektal Vuxen' = t1.RakelIndex1 THEN 'Bleeding'

WHEN 'Blödning Urinväg' = t1.RakelIndex1 THEN 'Bleeding'

WHEN 'Blödning Urinväg' = t1.RakelIndex1 THEN 'Bleeding'

WHEN 'Blödning Urinväg Vuxen' = t1.RakelIndex1 THEN 'Bleeding'

WHEN 'Blödning Urinväg Vuxen' = t1.RakelIndex1 THEN 'Bleeding'

WHEN 'Blödning Vaginal' = t1.RakelIndex1 THEN 'Bleeding'

WHEN 'Blödning Vaginal' = t1.RakelIndex1 THEN 'Bleeding'

WHEN 'Blödning Vaginal Vuxen' = t1.RakelIndex1 THEN 'Bleeding'

WHEN 'Blödning Vaginal Vuxen' = t1.RakelIndex1 THEN 'Bleeding'

WHEN 'Blödning. ej trauma' = t1.RakelIndex1 THEN 'Bleeding'

WHEN 'Blödning. ej trauma' = t1.RakelIndex1 THEN 'Bleeding'

WHEN 'Brännskada' = t1.RakelIndex1 THEN 'Burn'

WHEN 'Brännskada' = t1.RakelIndex1 THEN 'Burn'

WHEN 'Brännskada Barn 7-15år' = t1.RakelIndex1 THEN 'Burn'

WHEN 'Brännskada Barn 7-15år' = t1.RakelIndex1 THEN 'Burn'

WHEN 'Brännskada Vuxen' = t1.RakelIndex1 THEN 'Burn'

WHEN 'Brännskada Vuxen' = t1.RakelIndex1 THEN 'Burn'

WHEN 'Brännskada/Elolycka' = t1.RakelIndex1 THEN 'Burn'

WHEN 'Brännskada/Elolycka' = t1.RakelIndex1 THEN 'Burn'

WHEN 'Brännskada/Elolycka Barn 1-6 år' = t1.RakelIndex1 THEN 'Burn'

WHEN 'Brännskada/Elolycka Barn 1-6 år' = t1.RakelIndex1 THEN 'Burn'

WHEN 'Brännskada/Elskada' = t1.RakelIndex1 THEN 'Burn'

WHEN 'Brännskada/Elskada' = t1.RakelIndex1 THEN 'Burn'

WHEN 'Bröstsmärta/Hjärtsjukdom' = t1.RakelIndex1 THEN 'Chest pain'

WHEN 'Bröstsmärta/Hjärtsjukdom' = t1.RakelIndex1 THEN 'Chest pain'

WHEN 'Bröstsmärtor/Hjärtsjukdom' = t1.RakelIndex1 THEN 'Chest pain'

WHEN 'Bröstsmärtor/Hjärtsjukdom' = t1.RakelIndex1 THEN 'Chest pain'

WHEN 'Buk/urinvägar' = t1.RakelIndex1 THEN 'Abdominal pain'

WHEN 'Buk/urinvägar' = t1.RakelIndex1 THEN 'Abdominal pain'

WHEN 'Cykelolycka' = t1.RakelIndex1 THEN 'Bicycle accident'

WHEN 'Cykelolycka' = t1.RakelIndex1 THEN 'Bicycle accident'

WHEN 'Cykelolycka Vuxen' = t1.RakelIndex1 THEN 'Bicycle accident'

WHEN 'Cykelolycka Vuxen' = t1.RakelIndex1 THEN 'Bicycle accident'

WHEN 'Diabetes' = t1.RakelIndex1 THEN 'Diabetes'

WHEN 'Diabetes' = t1.RakelIndex1 THEN 'Diabetes'

WHEN 'Diarré/Kräkning' = t1.RakelIndex1 THEN 'Diarrhea/vomiting'

WHEN 'Diarré/Kräkning' = t1.RakelIndex1 THEN 'Diarrhea/vomiting'

WHEN 'Diarré/Kräkning Barn 1-6 år' = t1.RakelIndex1 THEN 'Diarrhea/vomiting'

WHEN 'Diarré/Kräkning Barn 1-6 år' = t1.RakelIndex1 THEN 'Diarrhea/vomiting'

WHEN 'Diarré/Kräkning Barn 7-15 år' = t1.RakelIndex1 THEN 'Diarrhea/vomiting'

WHEN 'Diarré/Kräkning Barn 7-15 år' = t1.RakelIndex1 THEN 'Diarrhea/vomiting'

WHEN 'Diarré/Kräkning Spädbarn' = t1.RakelIndex1 THEN 'Diarrhea/vomiting'

WHEN 'Diarré/Kräkning Spädbarn' = t1.RakelIndex1 THEN 'Diarrhea/vomiting'

WHEN 'Diarré/Kräkning Vuxen' = t1.RakelIndex1 THEN 'Diarrhea/vomiting'

WHEN 'Diarré/Kräkning Vuxen' = t1.RakelIndex1 THEN 'Diarrhea/vomiting'

WHEN 'Djurbett/Insektsstick' = t1.RakelIndex1 THEN 'Bite/Sting'

WHEN 'Djurbett/Insektsstick' = t1.RakelIndex1 THEN 'Bite/Sting'

WHEN 'Drunkningstillbud' = t1.RakelIndex1 THEN 'Drowning accident'

WHEN 'Drunkningstillbud' = t1.RakelIndex1 THEN 'Drowning accident'

WHEN 'Dykeriolycka' = t1.RakelIndex1 THEN 'Drowning accident'

WHEN 'Dykeriolycka' = t1.RakelIndex1 THEN 'Drowning accident'

WHEN 'Elolycka Vuxen' = t1.RakelIndex1 THEN 'Electrical accident'

WHEN 'Elolycka Vuxen' = t1.RakelIndex1 THEN 'Electrical accident'

WHEN 'Extremitet/Sårskador/Mindre trauma' = t1.RakelIndex1 THEN 'Extremity/Wounds/Minor Trauma'

WHEN 'Extremitet/Sårskador/Mindre trauma' = t1.RakelIndex1 THEN 'Extremity/Wounds/Minor Trauma'

WHEN 'Fastklämd person' = t1.RakelIndex1 THEN 'Trapped person'

WHEN 'Fastklämd person' = t1.RakelIndex1 THEN 'Trapped person'

WHEN 'Feber' = t1.RakelIndex1 THEN 'Fever/Infection'

WHEN 'Feber' = t1.RakelIndex1 THEN 'Fever/Infection'

WHEN 'Feber Barn 7-15 år' = t1.RakelIndex1 THEN 'Fever/Infection'

WHEN 'Feber Barn 7-15 år' = t1.RakelIndex1 THEN 'Fever/Infection'

WHEN 'Feber Vuxen' = t1.RakelIndex1 THEN 'Fever/Infection'

WHEN 'Feber Vuxen' = t1.RakelIndex1 THEN 'Fever/Infection'

WHEN 'Feber/Infektion' = t1.RakelIndex1 THEN 'Fever/Infection'

WHEN 'Feber/Infektion' = t1.RakelIndex1 THEN 'Fever/Infection'

WHEN 'Förändrat beteende' = t1.RakelIndex1 THEN 'Changed behavior'

WHEN 'Förändrat beteende' = t1.RakelIndex1 THEN 'Changed behavior'

WHEN 'Förändrat beteende Barn 1-6 år' = t1.RakelIndex1 THEN 'Changed behavior'

WHEN 'Förändrat beteende Barn 1-6 år' = t1.RakelIndex1 THEN 'Changed behavior'

WHEN 'Förändrat beteende Barn 7-15 år' = t1.RakelIndex1 THEN 'Changed behavior'

WHEN 'Förändrat beteende Barn 7-15 år' = t1.RakelIndex1 THEN 'Changed behavior'

WHEN 'Förändrat beteende Spädbarn' = t1.RakelIndex1 THEN 'Changed behavior'

WHEN 'Förändrat beteende Spädbarn' = t1.RakelIndex1 THEN 'Changed behavior'

WHEN 'Förändrat beteende Vuxen' = t1.RakelIndex1 THEN 'Changed behavior'

WHEN 'Förändrat beteende Vuxen' = t1.RakelIndex1 THEN 'Changed behavior'

WHEN 'Förgiftning' = t1.RakelIndex1 THEN 'Poisoning'

WHEN 'Förgiftning' = t1.RakelIndex1 THEN 'Poisoning'

WHEN 'Förgiftning Barn 1-6 år' = t1.RakelIndex1 THEN 'Poisoning'

WHEN 'Förgiftning Barn 1-6 år' = t1.RakelIndex1 THEN 'Poisoning'

WHEN 'Förgiftning Barn 7-15 år' = t1.RakelIndex1 THEN 'Poisoning'

WHEN 'Förgiftning Barn 7-15 år' = t1.RakelIndex1 THEN 'Poisoning'

WHEN 'Förgiftning Barn 7-15år' = t1.RakelIndex1 THEN 'Poisoning'

WHEN 'Förgiftning Barn 7-15år' = t1.RakelIndex1 THEN 'Poisoning'

WHEN 'Förgiftning Vuxen' = t1.RakelIndex1 THEN 'Poisoning'

WHEN 'Förgiftning Vuxen' = t1.RakelIndex1 THEN 'Poisoning'

WHEN 'Förgiftning, överdos' = t1.RakelIndex1 THEN 'Poisoning'

WHEN 'Förgiftning, överdos' = t1.RakelIndex1 THEN 'Poisoning'

WHEN 'Förlossning' = t1.RakelIndex1 THEN 'Childbirth'

WHEN 'Förlossning' = t1.RakelIndex1 THEN 'Childbirth'

WHEN 'Förlossning Vuxen' = t1.RakelIndex1 THEN 'Childbirth'

WHEN 'Förlossning Vuxen' = t1.RakelIndex1 THEN 'Childbirth'

WHEN 'Graviditet' = t1.RakelIndex1 THEN 'Pregnancy'

WHEN 'Graviditet' = t1.RakelIndex1 THEN 'Pregnancy'

WHEN 'Graviditet Vuxen' = t1.RakelIndex1 THEN 'Pregnancy'

WHEN 'Graviditet Vuxen' = t1.RakelIndex1 THEN 'Pregnancy'

WHEN 'Graviditet/förlossning (från v.20)' = t1.RakelIndex1 THEN 'Pregnancy'

WHEN 'Graviditet/förlossning (från v.20)' = t1.RakelIndex1 THEN 'Pregnancy'

WHEN 'Gyn-graviditet (före v.20)' = t1.RakelIndex1 THEN 'Pregnancy'

WHEN 'Gyn-graviditet (före v.20)' = t1.RakelIndex1 THEN 'Pregnancy'

WHEN 'Hästolycka' = t1.RakelIndex1 THEN 'Horse accident'

WHEN 'Hästolycka' = t1.RakelIndex1 THEN 'Horse accident'

WHEN 'Hästolycka Barn 7-15 år' = t1.RakelIndex1 THEN 'Horse accident'

WHEN 'Hästolycka Barn 7-15 år' = t1.RakelIndex1 THEN 'Horse accident'

WHEN 'Hästolycka Vuxen' = t1.RakelIndex1 THEN 'Horse accident'

WHEN 'Hästolycka Vuxen' = t1.RakelIndex1 THEN 'Horse accident'

WHEN 'Hjärtklappning' = t1.RakelIndex1 THEN 'Chest pain'

WHEN 'Hjärtklappning' = t1.RakelIndex1 THEN 'Chest pain'

WHEN 'Hjärtklappning Barn 7-15 år' = t1.RakelIndex1 THEN 'Chest pain'

WHEN 'Hjärtklappning Barn 7-15 år' = t1.RakelIndex1 THEN 'Chest pain'

WHEN 'Hjärtklappning Vuxen' = t1.RakelIndex1 THEN 'Chest pain'

WHEN 'Hjärtklappning Vuxen' = t1.RakelIndex1 THEN 'Chest pain'

WHEN 'Hjärtstopp' = t1.RakelIndex1 THEN 'Cardiac arrest'

WHEN 'Hjärtstopp' = t1.RakelIndex1 THEN 'Cardiac arrest'

WHEN 'Hjärtstopp Barn 1-6 år' = t1.RakelIndex1 THEN 'Cardiac arrest'

WHEN 'Hjärtstopp Barn 1-6 år' = t1.RakelIndex1 THEN 'Cardiac arrest'

WHEN 'Hjärtstopp Spädbarn' = t1.RakelIndex1 THEN 'Cardiac arrest'

WHEN 'Hjärtstopp Spädbarn' = t1.RakelIndex1 THEN 'Cardiac arrest'

WHEN 'Hjärtstopp Vuxen' = t1.RakelIndex1 THEN 'Cardiac arrest'

WHEN 'Hjärtstopp Vuxen' = t1.RakelIndex1 THEN 'Cardiac arrest'

WHEN 'Högenergitrauma' = t1.RakelIndex1 THEN 'High energy trauma'

WHEN 'Högenergitrauma' = t1.RakelIndex1 THEN 'High energy trauma'

WHEN 'Högenergitrauma Barn 1-6 år' = t1.RakelIndex1 THEN 'High energy trauma'

WHEN 'Högenergitrauma Barn 1-6 år' = t1.RakelIndex1 THEN 'High energy trauma'

WHEN 'Högenergitrauma Barn 7-15 år' = t1.RakelIndex1 THEN 'High energy trauma'

WHEN 'Högenergitrauma Barn 7-15 år' = t1.RakelIndex1 THEN 'High energy trauma'

WHEN 'Högenergitrauma Spädbarn' = t1.RakelIndex1 THEN 'High energy trauma'

WHEN 'Högenergitrauma Spädbarn' = t1.RakelIndex1 THEN 'High energy trauma'

WHEN 'Högenergitrauma Vuxen' = t1.RakelIndex1 THEN 'High energy trauma'

WHEN 'Högenergitrauma Vuxen' = t1.RakelIndex1 THEN 'High energy trauma'

WHEN 'Högt/Lågt sockervärde' = t1.RakelIndex1 THEN 'Diabetes'

WHEN 'Högt/Lågt sockervärde' = t1.RakelIndex1 THEN 'Diabetes'

WHEN 'Högt/Lågt sockervärde Barn 7-15 år' = t1.RakelIndex1 THEN 'Diabetes'

WHEN 'Högt/Lågt sockervärde Barn 7-15 år' = t1.RakelIndex1 THEN 'Diabetes'

WHEN 'Högt/Lågt sockervärde Vuxen' = t1.RakelIndex1 THEN 'Diabetes'

WHEN 'Högt/Lågt sockervärde Vuxen' = t1.RakelIndex1 THEN 'Diabetes'

WHEN 'Hot om suicid' = t1.RakelIndex1 THEN 'Threats of suicide'

WHEN 'Hot om suicid' = t1.RakelIndex1 THEN 'Threats of suicide'

WHEN 'Hot om suicid Barn 7-15 år' = t1.RakelIndex1 THEN 'Threats of suicide'

WHEN 'Hot om suicid Barn 7-15 år' = t1.RakelIndex1 THEN 'Threats of suicide'

WHEN 'Hot om suicid kroppskada' = t1.RakelIndex1 THEN 'Threats of suicide'

WHEN 'Hot om suicid kroppskada' = t1.RakelIndex1 THEN 'Threats of suicide'

WHEN 'Hot om suicid kroppskada Vuxen' = t1.RakelIndex1 THEN 'Threats of suicide'

WHEN 'Hot om suicid kroppskada Vuxen' = t1.RakelIndex1 THEN 'Threats of suicide'

WHEN 'Hot om suicid saknad person' = t1.RakelIndex1 THEN 'Threats of suicide'

WHEN 'Hot om suicid saknad person' = t1.RakelIndex1 THEN 'Threats of suicide'

WHEN 'Hot om suicid svår belägenhet' = t1.RakelIndex1 THEN 'Threats of suicide'

WHEN 'Hot om suicid svår belägenhet' = t1.RakelIndex1 THEN 'Threats of suicide'

WHEN 'Hot om suicid Vuxen' = t1.RakelIndex1 THEN 'Threats of suicide'

WHEN 'Hot om suicid Vuxen' = t1.RakelIndex1 THEN 'Threats of suicide'

WHEN 'Hotad luftväg' = t1.RakelIndex1 THEN 'Compromised airway'

WHEN 'Hotad luftväg' = t1.RakelIndex1 THEN 'Compromised airway'

WHEN 'Hotad luftväg Barn 1-6 år' = t1.RakelIndex1 THEN 'Compromised airway'

WHEN 'Hotad luftväg Barn 1-6 år' = t1.RakelIndex1 THEN 'Compromised airway'

WHEN 'Hotad luftväg Barn 7-15 år' = t1.RakelIndex1 THEN 'Compromised airway'

WHEN 'Hotad luftväg Barn 7-15 år' = t1.RakelIndex1 THEN 'Compromised airway'

WHEN 'Hotad luftväg Spädbarn' = t1.RakelIndex1 THEN 'Compromised airway'

WHEN 'Hotad luftväg Spädbarn' = t1.RakelIndex1 THEN 'Compromised airway'

WHEN 'Hotad luftväg Vuxen' = t1.RakelIndex1 THEN 'Compromised airway'

WHEN 'Hotad luftväg Vuxen' = t1.RakelIndex1 THEN 'Compromised airway'

WHEN 'Hudutslag' = t1.RakelIndex1 THEN 'Rash'

WHEN 'Hudutslag' = t1.RakelIndex1 THEN 'Rash'

WHEN 'Hudutslag Barn 1-6 år' = t1.RakelIndex1 THEN 'Rash'

WHEN 'Hudutslag Barn 1-6 år' = t1.RakelIndex1 THEN 'Rash'

WHEN 'Hudutslag Barn 7-15 år' = t1.RakelIndex1 THEN 'Rash'

WHEN 'Hudutslag Barn 7-15 år' = t1.RakelIndex1 THEN 'Rash'

WHEN 'Hudutslag Spädbarn' = t1.RakelIndex1 THEN 'Rash'

WHEN 'Hudutslag Spädbarn' = t1.RakelIndex1 THEN 'Rash'

WHEN 'Hudutslag Vuxen' = t1.RakelIndex1 THEN 'Rash'

WHEN 'Hudutslag Vuxen' = t1.RakelIndex1 THEN 'Rash'

WHEN 'Huvudvärk. Yrsel' = t1.RakelIndex1 THEN 'Headache'

WHEN 'Huvudvärk. Yrsel' = t1.RakelIndex1 THEN 'Headache'

WHEN 'Hypertermi Vuxen' = t1.RakelIndex1 THEN 'Hypo/Hyperthermia'

WHEN 'Hypertermi Vuxen' = t1.RakelIndex1 THEN 'Hypo/Hyperthermia'

WHEN 'Hypo/Hypertermi' = t1.RakelIndex1 THEN 'Hypo/Hyperthermia'

WHEN 'Hypo/Hypertermi' = t1.RakelIndex1 THEN 'Hypo/Hyperthermia'

WHEN 'Hypotermi/Köldskada' = t1.RakelIndex1 THEN 'Hypo/Hyperthermia'

WHEN 'Hypotermi/Köldskada' = t1.RakelIndex1 THEN 'Hypo/Hyperthermia'

WHEN 'Hypotermi/Köldskada Vuxen' = t1.RakelIndex1 THEN 'Hypo/Hyperthermia'

WHEN 'Hypotermi/Köldskada Vuxen' = t1.RakelIndex1 THEN 'Hypo/Hyperthermia'

WHEN 'Infektion' = t1.RakelIndex1 THEN 'Fever/Infection'

WHEN 'Infektion' = t1.RakelIndex1 THEN 'Fever/Infection'

WHEN 'Infektion Barn 1-6 år' = t1.RakelIndex1 THEN 'Fever/Infection'

WHEN 'Infektion Barn 1-6 år' = t1.RakelIndex1 THEN 'Fever/Infection'

WHEN 'Infektion Spädbarn' = t1.RakelIndex1 THEN 'Fever/Infection'

WHEN 'Infektion Spädbarn' = t1.RakelIndex1 THEN 'Fever/Infection'

WHEN 'Infektion/Misstanke om Sepsis Barn 7-15 år' = t1.RakelIndex1 THEN 'Fever/Infection'

WHEN 'Infektion/Misstanke om Sepsis Barn 7-15 år' = t1.RakelIndex1 THEN 'Fever/Infection'

WHEN 'Infektion/Misstanke om Sepsis Vuxen' = t1.RakelIndex1 THEN 'Fever/Infection'

WHEN 'Infektion/Misstanke om Sepsis Vuxen' = t1.RakelIndex1 THEN 'Fever/Infection'

WHEN 'Känsel/Motoriskt bortfall' = t1.RakelIndex1 THEN 'Sensation/motor loss'

WHEN 'Känsel/Motoriskt bortfall' = t1.RakelIndex1 THEN 'Sensation/motor loss'

WHEN 'Känsel/Motoriskt bortfall Barn 7-15år' = t1.RakelIndex1 THEN 'Sensation/motor loss'

WHEN 'Känsel/Motoriskt bortfall Barn 7-15år' = t1.RakelIndex1 THEN 'Sensation/motor loss'

WHEN 'Känsel/Motoriskt bortfall Vuxen' = t1.RakelIndex1 THEN 'Sensation/motor loss'

WHEN 'Känsel/Motoriskt bortfall Vuxen' = t1.RakelIndex1 THEN 'Sensation/motor loss'

WHEN 'Kateterbesvär' = t1.RakelIndex1 THEN 'Catheter discomfort'

WHEN 'Kateterbesvär' = t1.RakelIndex1 THEN 'Catheter discomfort'

WHEN 'Kateterbesvär Vuxen' = t1.RakelIndex1 THEN 'Catheter discomfort'

WHEN 'Kateterbesvär Vuxen' = t1.RakelIndex1 THEN 'Catheter discomfort'

WHEN 'Kemikalier-gaser' = t1.RakelIndex1 THEN 'Chemical exposure'

WHEN 'Kemikalier-gaser' = t1.RakelIndex1 THEN 'Chemical exposure'

WHEN 'Komplikation operation' = t1.RakelIndex1 THEN 'Complication after surgery'

WHEN 'Komplikation operation' = t1.RakelIndex1 THEN 'Complication after surgery'

WHEN 'Komplikation operation Barn 7-15 år' = t1.RakelIndex1 THEN 'Complication after surgery'

WHEN 'Komplikation operation Barn 7-15 år' = t1.RakelIndex1 THEN 'Complication after surgery'

WHEN 'Komplikation operation Barn 7-15år' = t1.RakelIndex1 THEN 'Complication after surgery'

WHEN 'Komplikation operation Barn 7-15år' = t1.RakelIndex1 THEN 'Complication after surgery'

WHEN 'Komplikation operation Vuxen' = t1.RakelIndex1 THEN 'Complication after surgery'

WHEN 'Komplikation operation Vuxen' = t1.RakelIndex1 THEN 'Complication after surgery'

WHEN 'Krampanfall' = t1.RakelIndex1 THEN 'Convulsion'

WHEN 'Krampanfall' = t1.RakelIndex1 THEN 'Convulsion'

WHEN 'Kramper/Epilepsi' = t1.RakelIndex1 THEN 'Convulsion'

WHEN 'Kramper/Epilepsi' = t1.RakelIndex1 THEN 'Convulsion'

WHEN 'Kramper/Epilepsi Barn 1-6 år' = t1.RakelIndex1 THEN 'Convulsion'

WHEN 'Kramper/Epilepsi Barn 1-6 år' = t1.RakelIndex1 THEN 'Convulsion'

WHEN 'Kramper/Epilepsi Barn 7-15 år' = t1.RakelIndex1 THEN 'Convulsion'

WHEN 'Kramper/Epilepsi Barn 7-15 år' = t1.RakelIndex1 THEN 'Convulsion'

WHEN 'Kramper/Epilepsi Barn 7-15år' = t1.RakelIndex1 THEN 'Convulsion'

WHEN 'Kramper/Epilepsi Barn 7-15år' = t1.RakelIndex1 THEN 'Convulsion'

WHEN 'Kramper/Epilepsi Spädbarn' = t1.RakelIndex1 THEN 'Convulsion'

WHEN 'Kramper/Epilepsi Spädbarn' = t1.RakelIndex1 THEN 'Convulsion'

WHEN 'Kramper/Epilepsi Vuxen' = t1.RakelIndex1 THEN 'Convulsion'

WHEN 'Kramper/Epilepsi Vuxen' = t1.RakelIndex1 THEN 'Convulsion'

WHEN 'Medvetandesänkt' = t1.RakelIndex1 THEN 'Unconsciousness/Decreased consciousness'

WHEN 'Medvetandesänkt' = t1.RakelIndex1 THEN 'Unconsciousness/Decreased consciousness'

WHEN 'Medvetandesänkt Barn 1-6 år' = t1.RakelIndex1 THEN 'Unconsciousness/Decreased consciousness'

WHEN 'Medvetandesänkt Barn 1-6 år' = t1.RakelIndex1 THEN 'Unconsciousness/Decreased consciousness'

WHEN 'Medvetandesänkt Barn 7-15 år' = t1.RakelIndex1 THEN 'Unconsciousness/Decreased consciousness'

WHEN 'Medvetandesänkt Barn 7-15 år' = t1.RakelIndex1 THEN 'Unconsciousness/Decreased consciousness'

WHEN 'Medvetandesänkt Spädbarn' = t1.RakelIndex1 THEN 'Unconsciousness/Decreased consciousness'

WHEN 'Medvetandesänkt Spädbarn' = t1.RakelIndex1 THEN 'Unconsciousness/Decreased consciousness'

WHEN 'Medvetandesänkt Vuxen' = t1.RakelIndex1 THEN 'Unconsciousness/Decreased consciousness'

WHEN 'Medvetandesänkt Vuxen' = t1.RakelIndex1 THEN 'Unconsciousness/Decreased consciousness'

WHEN 'Medvetslös' = t1.RakelIndex1 THEN 'Unconsciousness/Decreased consciousness'

WHEN 'Medvetslös' = t1.RakelIndex1 THEN 'Unconsciousness/Decreased consciousness'

WHEN 'Medvetslös Barn 1-6 år' = t1.RakelIndex1 THEN 'Unconsciousness/Decreased consciousness'

WHEN 'Medvetslös Barn 1-6 år' = t1.RakelIndex1 THEN 'Unconsciousness/Decreased consciousness'

WHEN 'Medvetslös Barn 7-15 år' = t1.RakelIndex1 THEN 'Unconsciousness/Decreased consciousness'

WHEN 'Medvetslös Barn 7-15 år' = t1.RakelIndex1 THEN 'Unconsciousness/Decreased consciousness'

WHEN 'Medvetslös Spädbarn' = t1.RakelIndex1 THEN 'Unconsciousness/Decreased consciousness'

WHEN 'Medvetslös Spädbarn' = t1.RakelIndex1 THEN 'Unconsciousness/Decreased consciousness'

WHEN 'Medvetslös vuxen' = t1.RakelIndex1 THEN 'Unconsciousness/Decreased consciousness'

WHEN 'Medvetslös vuxen' = t1.RakelIndex1 THEN 'Unconsciousness/Decreased consciousness'

WHEN 'Medvetslös Vuxen' = t1.RakelIndex1 THEN 'Unconsciousness/Decreased consciousness'

WHEN 'Medvetslös Vuxen' = t1.RakelIndex1 THEN 'Unconsciousness/Decreased consciousness'

WHEN 'Medvetslös-barn' = t1.RakelIndex1 THEN 'Unconsciousness/Decreased consciousness'

WHEN 'Medvetslös-barn' = t1.RakelIndex1 THEN 'Unconsciousness/Decreased consciousness'

WHEN 'Medvetslös-vuxen' = t1.RakelIndex1 THEN 'Unconsciousness/Decreased consciousness'

WHEN 'Medvetslös-vuxen' = t1.RakelIndex1 THEN 'Unconsciousness/Decreased consciousness'

WHEN 'Mindre trauma (lågenergi)' = t1.RakelIndex1 THEN 'Low energy trauma'

WHEN 'Mindre trauma (lågenergi) Barn 1-6 år' = t1.RakelIndex1 THEN 'Low energy trauma'

WHEN 'Mindre trauma (lågenergi) Barn 7-15 år' = t1.RakelIndex1 THEN 'Low energy trauma'

WHEN 'Mindre trauma (lågenergi) Barn 7-15år' = t1.RakelIndex1 THEN 'Low energy trauma'

WHEN 'Mindre trauma (lågenergi) Spädbarn' = t1.RakelIndex1 THEN 'Low energy trauma'

WHEN 'Mindre trauma (lågenergi) Vuxen' = t1.RakelIndex1 THEN 'Low energy trauma'

WHEN 'Misshandel' = t1.RakelIndex1 THEN 'Assault'

WHEN 'Misshandel' = t1.RakelIndex1 THEN 'Assault'

WHEN 'Misshandel Barn 7-15 år' = t1.RakelIndex1 THEN 'Assault'

WHEN 'Misshandel Barn 7-15 år' = t1.RakelIndex1 THEN 'Assault'

WHEN 'Misshandel Vuxen' = t1.RakelIndex1 THEN 'Assault'

WHEN 'Misshandel Vuxen' = t1.RakelIndex1 THEN 'Assault'

WHEN 'Misstanke om Sepsis' = t1.RakelIndex1 THEN 'Fever/Infection'

WHEN 'Misstanke om Sepsis' = t1.RakelIndex1 THEN 'Fever/Infection'

WHEN 'Misstanke om sjukvårdsbehov' = t1.RakelIndex1 THEN 'Unclear need for care'

WHEN 'Misstanke om sjukvårdsbehov' = t1.RakelIndex1 THEN 'Unclear need for care'

WHEN 'Misstanke om stroke/TIA' = t1.RakelIndex1 THEN 'Suspected Stroke/TIA'

WHEN 'Misstanke om stroke/TIA' = t1.RakelIndex1 THEN 'Suspected Stroke/TIA'

WHEN 'Misstanke om Stroke/TIA' = t1.RakelIndex1 THEN 'Suspected Stroke/TIA'

WHEN 'Misstanke om Stroke/TIA' = t1.RakelIndex1 THEN 'Suspected Stroke/TIA'

WHEN 'Misstanke om Stroke/TIA Vuxen' = t1.RakelIndex1 THEN 'Suspected Stroke/TIA'

WHEN 'Misstanke om Stroke/TIA Vuxen' = t1.RakelIndex1 THEN 'Suspected Stroke/TIA'

WHEN 'Oklart vårdbehov' = t1.RakelIndex1 THEN 'Unclear need for care'

WHEN 'Oklart vårdbehov' = t1.RakelIndex1 THEN 'Unclear need for care'

WHEN 'Oklart vårdbehov Barn 1-6 år' = t1.RakelIndex1 THEN 'Unclear need for care'

WHEN 'Oklart vårdbehov Barn 1-6 år' = t1.RakelIndex1 THEN 'Unclear need for care'

WHEN 'Oklart vårdbehov Barn 7-15 år' = t1.RakelIndex1 THEN 'Unclear need for care'

WHEN 'Oklart vårdbehov Barn 7-15 år' = t1.RakelIndex1 THEN 'Unclear need for care'

WHEN 'Oklart vårdbehov Spädbarn' = t1.RakelIndex1 THEN 'Unclear need for care'

WHEN 'Oklart vårdbehov Spädbarn' = t1.RakelIndex1 THEN 'Unclear need for care'

WHEN 'Oklart vårdbehov Vuxen' = t1.RakelIndex1 THEN 'Unclear need for care'

WHEN 'Oklart vårdbehov Vuxen' = t1.RakelIndex1 THEN 'Unclear need for care'

WHEN 'Omedelbar Livsfara Vuxen (Temporär indexering)' = t1.RakelIndex1 THEN 'Immediate danger to life'

WHEN 'Omedelbar Livsfara Vuxen (Temporär indexering)' = t1.RakelIndex1 THEN 'Immediate danger to life'

WHEN 'Påkörd person - Trafikolycka' = t1.RakelIndex1 THEN 'Person hit by a vehicle'

WHEN 'Påkörd person - Trafikolycka' = t1.RakelIndex1 THEN 'Person hit by a vehicle'

WHEN 'Påkörd person - Tunnelbana' = t1.RakelIndex1 THEN 'Person hit by a vehicle'

WHEN 'Påkörd person - Tunnelbana' = t1.RakelIndex1 THEN 'Person hit by a vehicle'

WHEN 'Påverkat allmäntillstånd' = t1.RakelIndex1 THEN 'Affected general condition'

WHEN 'Påverkat allmäntillstånd' = t1.RakelIndex1 THEN 'Affected general condition'

WHEN 'Påverkat allmäntillstånd Vuxen' = t1.RakelIndex1 THEN 'Affected general condition'

WHEN 'Påverkat allmäntillstånd Vuxen' = t1.RakelIndex1 THEN 'Affected general condition'

WHEN 'Påverkat tal' = t1.RakelIndex1 THEN 'Suspected Stroke/TIA'

WHEN 'Påverkat tal' = t1.RakelIndex1 THEN 'Suspected Stroke/TIA'

WHEN 'Påverkat tal Vuxen' = t1.RakelIndex1 THEN 'Suspected Stroke/TIA'

WHEN 'Påverkat tal Vuxen' = t1.RakelIndex1 THEN 'Suspected Stroke/TIA'

WHEN 'PDV-Övriga vapen' = t1.RakelIndex1 THEN 'Ongoing deadly violence'

WHEN 'PDV-Övriga vapen' = t1.RakelIndex1 THEN 'Ongoing deadly violence'

WHEN 'Penetrerande trauma' = t1.RakelIndex1 THEN 'Penetrating trauma'

WHEN 'Penetrerande trauma' = t1.RakelIndex1 THEN 'Penetrating trauma'

WHEN 'Penetrerande trauma Vuxen' = t1.RakelIndex1 THEN 'Penetrating trauma'

WHEN 'Penetrerande trauma Vuxen' = t1.RakelIndex1 THEN 'Penetrating trauma'

WHEN 'Psykiatri' = t1.RakelIndex1 THEN 'Psychiatric illness'

WHEN 'Psykiatri' = t1.RakelIndex1 THEN 'Psychiatric illness'

WHEN 'Psykiatri Barn 7-15 år' = t1.RakelIndex1 THEN 'Psychiatric illness'

WHEN 'Psykiatri Barn 7-15 år' = t1.RakelIndex1 THEN 'Psychiatric illness'

WHEN 'Psykiatri Vuxen' = t1.RakelIndex1 THEN 'Psychiatric illness'

WHEN 'Psykiatri Vuxen' = t1.RakelIndex1 THEN 'Psychiatric illness'

WHEN 'Slaganfall (Stroke)-förlamningar' = t1.RakelIndex1 THEN 'Suspected Stroke/TIA'

WHEN 'Slaganfall (Stroke)-förlamningar' = t1.RakelIndex1 THEN 'Suspected Stroke/TIA'

WHEN 'Smärta' = t1.RakelIndex1 THEN 'Pain complaints'

WHEN 'Smärta' = t1.RakelIndex1 THEN 'Pain complaints'

WHEN 'Smärta Arm' = t1.RakelIndex1 THEN 'Pain complaints'

WHEN 'Smärta Arm' = t1.RakelIndex1 THEN 'Pain complaints'

WHEN 'Smärta Arm Vuxen' = t1.RakelIndex1 THEN 'Pain complaints'

WHEN 'Smärta Arm Vuxen' = t1.RakelIndex1 THEN 'Pain complaints'

WHEN 'Smärta Barn 1-6 år' = t1.RakelIndex1 THEN 'Pain complaints'

WHEN 'Smärta Barn 1-6 år' = t1.RakelIndex1 THEN 'Pain complaints'

WHEN 'Smärta Ben' = t1.RakelIndex1 THEN 'Pain complaints'

WHEN 'Smärta Ben' = t1.RakelIndex1 THEN 'Pain complaints'

WHEN 'Smärta Ben Vuxen' = t1.RakelIndex1 THEN 'Pain complaints'

WHEN 'Smärta Ben Vuxen' = t1.RakelIndex1 THEN 'Pain complaints'

WHEN 'Smärta Bröst' = t1.RakelIndex1 THEN 'Pain complaints'

WHEN 'Smärta Bröst' = t1.RakelIndex1 THEN 'Pain complaints'

WHEN 'Smärta Bröst Barn 7-15 år' = t1.RakelIndex1 THEN 'Pain complaints'

WHEN 'Smärta Bröst Barn 7-15 år' = t1.RakelIndex1 THEN 'Pain complaints'

WHEN 'Smärta Bröst Vuxen' = t1.RakelIndex1 THEN 'Pain complaints'

WHEN 'Smärta Bröst Vuxen' = t1.RakelIndex1 THEN 'Pain complaints'

WHEN 'Smärta Buk' = t1.RakelIndex1 THEN 'Pain complaints'

WHEN 'Smärta Buk' = t1.RakelIndex1 THEN 'Pain complaints'

WHEN 'Smärta Buk Barn 7-15 år' = t1.RakelIndex1 THEN 'Pain complaints'

WHEN 'Smärta Buk Barn 7-15 år' = t1.RakelIndex1 THEN 'Pain complaints'

WHEN 'Smärta Buk Vuxen' = t1.RakelIndex1 THEN 'Pain complaints'

WHEN 'Smärta Buk Vuxen' = t1.RakelIndex1 THEN 'Pain complaints'

WHEN 'Smärta Extremitet' = t1.RakelIndex1 THEN 'Pain complaints'

WHEN 'Smärta Extremitet' = t1.RakelIndex1 THEN 'Pain complaints'

WHEN 'Smärta Extremitet Barn 7-15 år' = t1.RakelIndex1 THEN 'Pain complaints'

WHEN 'Smärta Extremitet Barn 7-15 år' = t1.RakelIndex1 THEN 'Pain complaints'

WHEN 'Smärta Hals' = t1.RakelIndex1 THEN 'Pain complaints'

WHEN 'Smärta Hals' = t1.RakelIndex1 THEN 'Pain complaints'

WHEN 'Smärta Hals Vuxen' = t1.RakelIndex1 THEN 'Pain complaints'

WHEN 'Smärta Hals Vuxen' = t1.RakelIndex1 THEN 'Pain complaints'

WHEN 'Smärta Höft' = t1.RakelIndex1 THEN 'Pain complaints'

WHEN 'Smärta Höft' = t1.RakelIndex1 THEN 'Pain complaints'

WHEN 'Smärta Höft Vuxen' = t1.RakelIndex1 THEN 'Pain complaints'

WHEN 'Smärta Höft Vuxen' = t1.RakelIndex1 THEN 'Pain complaints'

WHEN 'Smärta Huvud' = t1.RakelIndex1 THEN 'Pain complaints'

WHEN 'Smärta Huvud' = t1.RakelIndex1 THEN 'Pain complaints'

WHEN 'Smärta Huvud Barn 7-15 år' = t1.RakelIndex1 THEN 'Pain complaints'

WHEN 'Smärta Huvud Barn 7-15 år' = t1.RakelIndex1 THEN 'Pain complaints'

WHEN 'Smärta Huvud Vuxen' = t1.RakelIndex1 THEN 'Pain complaints'

WHEN 'Smärta Huvud Vuxen' = t1.RakelIndex1 THEN 'Pain complaints'

WHEN 'Smärta Öga' = t1.RakelIndex1 THEN 'Pain complaints'

WHEN 'Smärta Öga' = t1.RakelIndex1 THEN 'Pain complaints'

WHEN 'Smärta Öga Vuxen' = t1.RakelIndex1 THEN 'Pain complaints'

WHEN 'Smärta Öga Vuxen' = t1.RakelIndex1 THEN 'Pain complaints'

WHEN 'Smärta Öra Vuxen' = t1.RakelIndex1 THEN 'Pain complaints'

WHEN 'Smärta Öra Vuxen' = t1.RakelIndex1 THEN 'Pain complaints'

WHEN 'Smärta Rygg (högt)' = t1.RakelIndex1 THEN 'Pain complaints'

WHEN 'Smärta Rygg (högt)' = t1.RakelIndex1 THEN 'Pain complaints'

WHEN 'Smärta Rygg (högt) Vuxen' = t1.RakelIndex1 THEN 'Pain complaints'

WHEN 'Smärta Rygg (högt) Vuxen' = t1.RakelIndex1 THEN 'Pain complaints'

WHEN 'Smärta Rygg (lågt)' = t1.RakelIndex1 THEN 'Pain complaints'

WHEN 'Smärta Rygg (lågt)' = t1.RakelIndex1 THEN 'Pain complaints'

WHEN 'Smärta Rygg (lågt) Vuxen' = t1.RakelIndex1 THEN 'Pain complaints'

WHEN 'Smärta Rygg (lågt) Vuxen' = t1.RakelIndex1 THEN 'Pain complaints'

WHEN 'Smärta Rygg (mellan)' = t1.RakelIndex1 THEN 'Pain complaints'

WHEN 'Smärta Rygg (mellan)' = t1.RakelIndex1 THEN 'Pain complaints'

WHEN 'Smärta Rygg (mellan) Vuxen' = t1.RakelIndex1 THEN 'Pain complaints'

WHEN 'Smärta Rygg (mellan) Vuxen' = t1.RakelIndex1 THEN 'Pain complaints'

WHEN 'Smärta Rygg Barn 7-15 år' = t1.RakelIndex1 THEN 'Pain complaints'

WHEN 'Smärta Rygg Barn 7-15 år' = t1.RakelIndex1 THEN 'Pain complaints'

WHEN 'Stroke -Förlamningar' = t1.RakelIndex1 THEN 'Suspected Stroke/TIA'

WHEN 'Stroke -Förlamningar' = t1.RakelIndex1 THEN 'Suspected Stroke/TIA'

WHEN 'Suicidmisstanke-psykiatri' = t1.RakelIndex1 THEN 'Psychiatric illness'

WHEN 'Suicidmisstanke-psykiatri' = t1.RakelIndex1 THEN 'Psychiatric illness'

WHEN 'Svimning' = t1.RakelIndex1 THEN 'Fainting'

WHEN 'Svimning' = t1.RakelIndex1 THEN 'Fainting'

WHEN 'Svimning Barn 7-15 år' = t1.RakelIndex1 THEN 'Fainting'

WHEN 'Svimning Barn 7-15 år' = t1.RakelIndex1 THEN 'Fainting'

WHEN 'Svimning Vuxen' = t1.RakelIndex1 THEN 'Fainting'

WHEN 'Svimning Vuxen' = t1.RakelIndex1 THEN 'Fainting'

WHEN 'Svullnad Ansikte' = t1.RakelIndex1 THEN 'Swelling'

WHEN 'Svullnad Ansikte' = t1.RakelIndex1 THEN 'Swelling'

WHEN 'Svullnad Ansikte Vuxen' = t1.RakelIndex1 THEN 'Swelling'

WHEN 'Svullnad Ansikte Vuxen' = t1.RakelIndex1 THEN 'Swelling'

WHEN 'Svullnad Barn 1-6 år' = t1.RakelIndex1 THEN 'Swelling'

WHEN 'Svullnad Barn 1-6 år' = t1.RakelIndex1 THEN 'Swelling'

WHEN 'Svullnad Barn 7-15 år' = t1.RakelIndex1 THEN 'Swelling'

WHEN 'Svullnad Buk' = t1.RakelIndex1 THEN 'Swelling'

WHEN 'Svullnad Buk Vuxen' = t1.RakelIndex1 THEN 'Swelling'

WHEN 'Svullnad Extremitet' = t1.RakelIndex1 THEN 'Swelling'

WHEN 'Svullnad Extremitet Vuxen' = t1.RakelIndex1 THEN 'Swelling'

WHEN 'Svullnad Spädbarn' = t1.RakelIndex1 THEN 'Swelling'

WHEN 'Synbortfall' = t1.RakelIndex1 THEN 'Vision loss'

WHEN 'Synbortfall Barn 7-15 år' = t1.RakelIndex1 THEN 'Vision loss'

WHEN 'Synbortfall Vuxen' = t1.RakelIndex1 THEN 'Vision loss'

WHEN 'Trafikolycka' = t1.RakelIndex1 THEN 'Person hit by a vehicle'

WHEN 'Trafikolycka' = t1.RakelIndex1 THEN 'Traffic accident'

WHEN 'Trubbigt trauma Vuxen' = t1.RakelIndex1 THEN 'Blunt Trauma'

WHEN 'Utsläpp' = t1.RakelIndex1 THEN 'Chemical exposure'

WHEN 'Våld-misshandel' = t1.RakelIndex1 THEN 'Assault'

WHEN 'Yrsel' = t1.RakelIndex1 THEN 'Dizziness'

WHEN 'Yrsel Barn 7-15 år' = t1.RakelIndex1 THEN 'Dizziness'

WHEN 'Yrsel Vuxen' = t1.RakelIndex1 THEN 'Dizziness'

ELSE 'Other'

END) AS ResonOfCallToEMCC,

/* KOMMUN */

(CASE

WHEN 'botkyrka' = t2.KOMMUN THEN 'Botkyrka'

WHEN 'Botkyrka' = t2.KOMMUN THEN 'Botkyrka'

WHEN 'danderyd' = t2.KOMMUN THEN 'Danderyd'

WHEN 'Danderyd' = t2.KOMMUN THEN 'Danderyd'

WHEN 'ekerö' = t2.KOMMUN THEN 'Ekerö'

WHEN 'Ekerö' = t2.KOMMUN THEN 'Ekerö'

WHEN 'haninge' = t2.KOMMUN THEN 'Haninge'

WHEN 'Haninge' = t2.KOMMUN THEN 'Haninge'

WHEN 'HAninge' = t2.KOMMUN THEN 'Haninge'

WHEN 'huddinge' = t2.KOMMUN THEN 'Huddinge'

WHEN 'Huddinge' = t2.KOMMUN THEN 'Huddinge'

WHEN 'HUDDINGE' = t2.KOMMUN THEN 'Huddinge'

WHEN 'järfälla' = t2.KOMMUN THEN 'Järfälla'

WHEN 'Järfälla' = t2.KOMMUN THEN 'Järfälla'

WHEN 'lidingö' = t2.KOMMUN THEN 'Lidingö'

WHEN 'Lidingö' = t2.KOMMUN THEN 'Lidingö'

WHEN 'nacka' = t2.KOMMUN THEN 'Nacka'

WHEN 'nackA' = t2.KOMMUN THEN 'Nacka'

WHEN 'Nacka' = t2.KOMMUN THEN 'Nacka'

WHEN 'NAcka' = t2.KOMMUN THEN 'Nacka'

WHEN 'norrtälje' = t2.KOMMUN THEN 'Norrtälje'

WHEN 'Norrtälje' = t2.KOMMUN THEN 'Norrtälje'

WHEN 'nORRTÄLJE' = t2.KOMMUN THEN 'Norrtälje'

WHEN 'nyköping' = t2.KOMMUN THEN 'Nyköping'

WHEN 'Nyköping' = t2.KOMMUN THEN 'Nyköping'

WHEN 'nYNäs' = t2.KOMMUN THEN 'Nynäshamn'

WHEN 'nynäshamn' = t2.KOMMUN THEN 'Nynäshamn'

WHEN 'Nynäshamn' = t2.KOMMUN THEN 'Nynäshamn'

WHEN 'NYNÄSHAMN' = t2.KOMMUN THEN 'Nynäshamn'

WHEN 'österåker' = t2.KOMMUN THEN 'Österåker'

WHEN 'Österåker' = t2.KOMMUN THEN 'Österåker'

WHEN 'salem' = t2.KOMMUN THEN 'Salem'

WHEN 'Salem' = t2.KOMMUN THEN 'Salem'

WHEN 'sigtuna' = t2.KOMMUN THEN 'Sigtuna'

WHEN 'Sigtuna' = t2.KOMMUN THEN 'Sigtuna'

WHEN 'SIGTUNA' = t2.KOMMUN THEN 'Sigtuna'

WHEN 'södertälje' = t2.KOMMUN THEN 'Södertälje'

WHEN 'Södertälje' = t2.KOMMUN THEN 'Södertälje'

WHEN 'sollentuna' = t2.KOMMUN THEN 'Sollentuna'

WHEN 'Sollentuna' = t2.KOMMUN THEN 'Sollentuna'

WHEN 'solna' = t2.KOMMUN THEN 'Solna'

WHEN 'Solna' = t2.KOMMUN THEN 'Solna'

WHEN 'sthlm' = t2.KOMMUN THEN 'Stockholm'

WHEN 'stockholm' = t2.KOMMUN THEN 'Stockholm'

WHEN 'sTOCKHOLM' = t2.KOMMUN THEN 'Stockholm'

WHEN 'Stockholm' = t2.KOMMUN THEN 'Stockholm'

WHEN 'STockholm' = t2.KOMMUN THEN 'Stockholm'

WHEN 'STOCKHOLM' = t2.KOMMUN THEN 'Stockholm'

WHEN 'stockhom' = t2.KOMMUN THEN 'Stockholm'

WHEN 'sundbyberg' = t2.KOMMUN THEN 'Sundbyberg'

WHEN 'Sundbyberg' = t2.KOMMUN THEN 'Sundbyberg'

WHEN 'täby' = t2.KOMMUN THEN 'Täby'

WHEN 'Täby' = t2.KOMMUN THEN 'Täby'

WHEN 'tyresö' = t2.KOMMUN THEN 'Tyresö'

WHEN 'Tyresö' = t2.KOMMUN THEN 'Tyresö'

WHEN 'upplands väsby' = t2.KOMMUN THEN 'Upplands Väsby'

WHEN 'Upplands väsby' = t2.KOMMUN THEN 'Upplands Väsby'

WHEN 'Upplands Väsby' = t2.KOMMUN THEN 'Upplands Väsby'

WHEN 'upplands-bro' = t2.KOMMUN THEN 'Upplands-Bro'

WHEN 'Upplands-bro' = t2.KOMMUN THEN 'Upplands-Bro'

WHEN 'Upplands-Bro' = t2.KOMMUN THEN 'Upplands-Bro'

WHEN 'vallentuna' = t2.KOMMUN THEN 'Vallentuna'

WHEN 'Vallentuna' = t2.KOMMUN THEN 'Vallentuna'

WHEN 'värmdö' = t2.KOMMUN THEN 'Värmdö'

WHEN 'Värmdö' = t2.KOMMUN THEN 'Värmdö'

WHEN 'vaxholm' = t2.KOMMUN THEN 'Vaxholm'

WHEN 'Vaxholm' = t2.KOMMUN THEN 'Vaxholm'

ELSE t2.KOMMUN

END) AS KOMMUN

FROM BI_KFA.DYNEMSALL_TASKLASTVALUE t1

LEFT JOIN BI_KFA.DYNEMSALL_INCIDENTLASTVALUE t2 ON (t1.incidentid_lopnr = t2.incidentid_lopnr)

WHERE t1.RakelIndex1 NOT IN

(

'Sekundäruppdrag',

'Sekundäruppdrag Barn 1-6 år',

'Sekundäruppdrag Barn 7-15 år',

'Sekundäruppdrag Spädbarn',

'Sekundäruppdrag Vuxen',

'Sjuktransporter',

'Beställt uppdrag mellan vårdenheter'

) AND t1.RakelIndex2 NOT IN

(

'Transport',

'Transport mellan vårdenheter',

'Transport till/från vårdenhet',

'Transport till/från vårdenhet (DOLD)',

'Transportuppdrag'

) AND t1.PRIORITETUT IN

(

'1',

'2',

'3'

) AND t1.EmsUnitType = 'Akutambulans' AND (CALCULATED Year) IN

(

2017,

2018,

2019,

2020,

2021,

2022

) AND (CALCULATED Call_handelingtime) BETWEEN 0 AND 36000 AND (CALCULATED Drivetime) BETWEEN 0 AND 36000 AND

(CALCULATED Onscenetime) BETWEEN 0 AND 36000 AND (CALCULATED Deliverytime) BETWEEN 0 AND 36000 AND

(CALCULATED Responsetime) BETWEEN 0 AND 36000 AND (CALCULATED Transportationtime) BETWEEN 0 AND 36000

GROUP BY (CALCULATED Responsetime),

t1.incidentid_lopnr,

t1.taskid_lopnr,

t1.rakelid_lopnr,

(CALCULATED 'Receved emergencycall UTC'n),

(CALCULATED Year),

(CALCULATED Hour),

t1.PRIORITETUT,

(CALCULATED 'DistancetoArrival km'n),

(CALCULATED Call_handelingtime),

(CALCULATED Drivetime),

(CALCULATED Date),

t1.RakelIndex1,

(CALCULATED Onscenetime),

(CALCULATED Transportationtime),

(CALCULATED Deliverytime),

(CALCULATED Month),

(CALCULATED Weekday),

t1.Station,

(CALCULATED ResonOfCallToEMCC),

(CALCULATED KOMMUN)

ORDER BY 'Receved emergencycall UTC'n;

QUIT;

%LET _CLIENTTASKLABEL=;

%LET _CLIENTPROCESSFLOWNAME=;

%LET _CLIENTPROJECTPATH=;

%LET _CLIENTPROJECTPATHHOST=;

%LET _CLIENTPROJECTNAME=;

/* START PÅ NOD: Frågebyggare */

%LET _CLIENTTASKLABEL='Frågebyggare';

%LET _CLIENTPROCESSFLOWNAME='Processflöde';

%LET _CLIENTPROJECTPATH='C:\Users\4khw\OneDrive - Karolinska Institutet\General\Studie I\Revision II\Analyses\Study I Revision 2.2.egp';

%LET _CLIENTPROJECTPATHHOST='RSTPC00020406';

%LET _CLIENTPROJECTNAME='Study I Revision 2.2.egp';

%_eg_conditional_dropds(WORK.QUERY_FOR_DYNEMSALL_TASKLAS_0000);

PROC SQL;

CREATE TABLE WORK.QUERY_FOR_DYNEMSALL_TASKLAS_0000 AS

SELECT t1.Date,

t1.Hour,

/* SUM_of_Resources */

(COUNT(DISTINCT(t1.rakelid_lopnr))) AS SUM_of_Resources,

/* SUM_of_MissionCanc */

(SUM(t1.MissionCancelled)) FORMAT=NUMX12. AS SUM_of_MissionCanc,

/* H_AVG_of_Onscenetime */

(AVG(t1.Onscenetime)) FORMAT=TIME8. AS H_AVG_of_Onscenetime,

/* H_AVG_of_Transportationtime */

(AVG(t1.Transportationtime)) FORMAT=TIME8. AS H_AVG_of_Transportationtime,

/* H_AVG_of_Deliverytime */

(AVG(t1.Deliverytime)) FORMAT=TIME8. AS H_AVG_of_Deliverytime,

/* C_DISTT_of_missions */

(COUNT(DISTINCT(t1.taskid_lopnr))) AS C_DISTT_of_missions,

/* H_AVG_of_Responsetime */

(AVG(t1.Responsetime)) FORMAT=TIME8. AS H_AVG_of_Responsetime,

/* H_AVG_of_Call_handelingtime */

(AVG(t1.Call_handelingtime)) FORMAT=TIME8. AS H_AVG_of_Call_handelingtime,

/* H_AVG_of_Drivetime */

(AVG(t1.Drivetime)) FORMAT=TIME8. AS H_AVG_of_Drivetime,

/* H_AVG_of_DistArrvalkm */

(AVG(t1.'DistancetoArrival km'n)) FORMAT=COMMAX6.3 AS H_AVG_of_DistArrvalkm

FROM WORK.QUERY_FOR_DYNEMSALL_TASKLASTVALU t1

WHERE t1.Responsetime BETWEEN '0:0:0't AND '10:0:0't

GROUP BY t1.Date,

t1.Hour;

QUIT;

%LET _CLIENTTASKLABEL=;

%LET _CLIENTPROCESSFLOWNAME=;

%LET _CLIENTPROJECTPATH=;

%LET _CLIENTPROJECTPATHHOST=;

%LET _CLIENTPROJECTNAME=;

/* START PÅ NOD: Partition Data */

%LET _CLIENTTASKLABEL='Partition Data';

%LET _CLIENTPROCESSFLOWNAME='Processflöde';

%LET _CLIENTPROJECTPATH='C:\Users\4khw\OneDrive - Karolinska Institutet\General\Studie I\Revision II\Analyses\Study I Revision 2.2.egp';

%LET _CLIENTPROJECTPATHHOST='RSTPC00020406';

%LET _CLIENTPROJECTNAME='Study I Revision 2.2.egp';

%macro web_drop_table / parmbuff;%mend;

%macro web_open_table / parmbuff;%mend;

ODS GRAPHICS ON;

TITLE;FOOTNOTE;

FOOTNOTE1 "Genererat av SAS (&_SASSERVERNAME, &SYSSCPL) %TRIM(%QSYSFUNC(DATE(), NLDATE20.)) %TRIM(%QSYSFUNC(TIME(), NLTIME.))";

data _null_;

idMaxLength=length("Training");

idMaxLength=max(idMaxLength, length("Validation"));

idMaxLength=max(idMaxLength, length("Test"));

/* Put it in a macro variable for use in the real code */

call symput('idLength', idMaxLength);

run;

proc sql noprint;

select count(*) into :count from WORK.STDIZE;

quit;

data J.DEMSI_P;

set WORK.STDIZE;

length _Partition_ $ &idLength;

retain __tmp1-__tmp%trim(&count) __nobs__ __nobs1__ __nobs2__ __nobs3__;

drop _i_ __seed__ __tmp1-__tmp%trim(&count);

drop _n1_ __nobs__ __nobs1__ __nobs2__ __nobs3__;

array __tmp(*) __tmp1-__tmp%trim(&count);

if (_n_=1) then

do;

__seed__=-1;

__nobs__=&count;

do _i_=1 to dim(__tmp);

__tmp(_i_)=_i_;

end;

call ranperm(__seed__, of __tmp(*));

__nobs1__=round(0.6*__nobs__);

__nobs2__=round(0.3*__nobs__)+__nobs1__;

__nobs3__=round(0.1*__nobs__)+__nobs2__;

end;

_n1_=_n_;

if (_n1_ <=dim(__tmp)) then

do;

if (__tmp(_n1_) > 0) then

do;

if (__tmp(_n1_) <=__nobs1__) then

do;

_Partition_="Training";

output;

end;

else if (__tmp(_n1_) <=__nobs2__) then

do;

_Partition_="Validation";

output;

end;

else if (__tmp(_n1_) <=__nobs3__) then

do;

_Partition_="Test";

output;

end;

end;

end;

run;

TITLE;FOOTNOTE;

%LET _CLIENTTASKLABEL=;

%LET _CLIENTPROCESSFLOWNAME=;

%LET _CLIENTPROJECTPATH=;

%LET _CLIENTPROJECTPATHHOST=;

%LET _CLIENTPROJECTNAME=;

/* START PÅ NOD: Describe Missing Data 1 */

%LET _CLIENTTASKLABEL='Describe Missing Data 1';

%LET _CLIENTPROCESSFLOWNAME='Processflöde';

%LET _CLIENTPROJECTPATH='C:\Users\4khw\OneDrive - Karolinska Institutet\General\Studie I\Revision II\Analyses\Study I Revision 2.2.egp';

%LET _CLIENTPROJECTPATHHOST='RSTPC00020406';

%LET _CLIENTPROJECTNAME='Study I Revision 2.2.egp';

%macro web_drop_table / parmbuff;%mend;

%macro web_open_table / parmbuff;%mend;

ODS GRAPHICS ON;

TITLE;FOOTNOTE;

FOOTNOTE1 "Genererat av SAS (&_SASSERVERNAME, &SYSSCPL) %TRIM(%QSYSFUNC(DATE(), NLDATE20.)) %TRIM(%QSYSFUNC(TIME(), NLTIME.))";

ods noproctitle;

proc format;

value _nmissprint low-high="Non-missing";

value $_cmissprint " "=" " other="Non-missing";

run;

proc freq data=BI_KFA.DYNEMSALL_TASKLASTVALUE;

title3 "Missing Data Frequencies";

title4 h=2 "Legend: ., A, B, etc = Missing";

format IDNR_Lopnr CreationTime Distance Kvitterat Passning Framme Lastat

Snart_klar Klar_uppdrag Avbrutet_uppdrag DKvitterat DPassning DFramme DLastat

DSnart_klar DKlar_uppdrag DAvbrutet_uppdrag GeoKvitterat200m GeoFramme200m

GeoLastat200m GeoSnartKlar200m taskid_lopnr incidentid_lopnr rakelid_lopnr

_nmissprint.;

format Operator EmsUnitType Station RakelIndex1 RakelIndex2 PRIORITETUT

PRIORITETIN LARM_POD LARMTIDAMBULANS MOTTAGEN INDEXNIVA1

INDEXNIVA2 $_cmissprint.;

tables IDNR_Lopnr CreationTime Operator EmsUnitType Station RakelIndex1

RakelIndex2 Distance Kvitterat Passning Framme Lastat Snart_klar Klar_uppdrag

Avbrutet_uppdrag DKvitterat DPassning DFramme DLastat DSnart_klar

DKlar_uppdrag DAvbrutet_uppdrag GeoKvitterat200m GeoFramme200m GeoLastat200m

GeoSnartKlar200m PRIORITETUT PRIORITETIN LARM_POD LARMTIDAMBULANS MOTTAGEN

INDEXNIVA1 INDEXNIVA2 taskid_lopnr incidentid_lopnr rakelid_lopnr / missing

nocum;

run;

proc freq data=BI_KFA.DYNEMSALL_TASKLASTVALUE noprint;

table IDNR_Lopnr * CreationTime * Operator * EmsUnitType * Station *

RakelIndex1 * RakelIndex2 * Distance * Kvitterat * Passning * Framme * Lastat

* Snart_klar * Klar_uppdrag * Avbrutet_uppdrag * DKvitterat * DPassning *

DFramme * DLastat * DSnart_klar * DKlar_uppdrag * DAvbrutet_uppdrag *

GeoKvitterat200m * GeoFramme200m * GeoLastat200m * GeoSnartKlar200m *

PRIORITETUT * PRIORITETIN * LARM_POD * LARMTIDAMBULANS * MOTTAGEN *

INDEXNIVA1 * INDEXNIVA2 * taskid_lopnr * incidentid_lopnr * rakelid_lopnr /

missing out=Work._MissingData_;

format IDNR_Lopnr CreationTime Distance Kvitterat Passning Framme Lastat

Snart_klar Klar_uppdrag Avbrutet_uppdrag DKvitterat DPassning DFramme DLastat

DSnart_klar DKlar_uppdrag DAvbrutet_uppdrag GeoKvitterat200m GeoFramme200m

GeoLastat200m GeoSnartKlar200m taskid_lopnr incidentid_lopnr rakelid_lopnr

_nmissprint.;

format Operator EmsUnitType Station RakelIndex1 RakelIndex2 PRIORITETUT

PRIORITETIN LARM_POD LARMTIDAMBULANS MOTTAGEN INDEXNIVA1

INDEXNIVA2 $_cmissprint.;

run;

proc print data=Work._MissingData_ noobs label;

title3 "Missing Data Patterns across Variables";

title4 h=2 "Legend: ., A, B, etc = Missing";

format IDNR_Lopnr CreationTime Distance Kvitterat Passning Framme Lastat

Snart_klar Klar_uppdrag Avbrutet_uppdrag DKvitterat DPassning DFramme DLastat

DSnart_klar DKlar_uppdrag DAvbrutet_uppdrag GeoKvitterat200m GeoFramme200m

GeoLastat200m GeoSnartKlar200m taskid_lopnr incidentid_lopnr rakelid_lopnr

_nmissprint.;

format Operator EmsUnitType Station RakelIndex1 RakelIndex2 PRIORITETUT

PRIORITETIN LARM_POD LARMTIDAMBULANS MOTTAGEN INDEXNIVA1

INDEXNIVA2 $_cmissprint.;

label count="Frequency" percent="Percent";

run;

title3;

/* Clean up */

proc delete data=Work._MissingData_;

run;

TITLE;FOOTNOTE;

%LET _CLIENTTASKLABEL=;

%LET _CLIENTPROCESSFLOWNAME=;

%LET _CLIENTPROJECTPATH=;

%LET _CLIENTPROJECTPATHHOST=;

%LET _CLIENTPROJECTNAME=;

/* START PÅ NOD: Program */

%LET _CLIENTTASKLABEL='Program';

%LET _CLIENTPROCESSFLOWNAME='Processflöde';

%LET _CLIENTPROJECTPATH='C:\Users\4khw\OneDrive - Karolinska Institutet\General\Studie I\Revision II\Analyses\Study I Revision 2.2.egp';

%LET _CLIENTPROJECTPATHHOST='RSTPC00020406';

%LET _CLIENTPROJECTNAME='Study I Revision 2.2.egp';

%LET _SASPROGRAMFILE='';

%LET _SASPROGRAMFILEHOST='';

libname va_viya meta library='VA_VIYA' metaout=data;

data va_viya.phill_test;

a='test';

b=1;

run;

%LET _CLIENTTASKLABEL=;

%LET _CLIENTPROCESSFLOWNAME=;

%LET _CLIENTPROJECTPATH=;

%LET _CLIENTPROJECTPATHHOST=;

%LET _CLIENTPROJECTNAME=;

%LET _SASPROGRAMFILE=;

%LET _SASPROGRAMFILEHOST=;

/* START PÅ NOD: Importera data (minmaxtemp.csv) */

%LET _CLIENTTASKLABEL='Importera data (minmaxtemp.csv)';

%LET _CLIENTPROCESSFLOWNAME='Processflöde';

%LET _CLIENTPROJECTPATH='C:\Users\4khw\OneDrive - Karolinska Institutet\General\Studie I\Revision II\Analyses\Study I Revision 2.2.egp';

%LET _CLIENTPROJECTPATHHOST='RSTPC00020406';

%LET _CLIENTPROJECTNAME='Study I Revision 2.2.egp';

/* --------------------------------------------------------------------

Kod genererad av en SAS-uppgift

Genererad 07 January 2025 08:38:07

Av uppgift: guiden Importera data

Källfil:

\\gainashsf01.gaia.sll.se\fs_hsf_usr$\4khw\Dokument\Projekt\Dynamic

EMS allocation\Dataanalys\weather\minmaxtemp.csv

Server: Lokalt filsystem

Output-data: WORK.minmaxtemp

Server: SASAppUser

Obs! Som föreberedelse inför körning av nedanstående kod har guiden

Importera data använt interna rutiner för att överföra

källdatafilen från det lokala filsystemet till SASAppUser. Det

finns ingen SAS-kod tillgänglig för att representera den här

åtgärden.

-------------------------------------------------------------------- */

DATA WORK.minmaxtemp;

LENGTH

'Representativt dygn'n 8

Lufttemperatur_min 8

Lufttemperatur_max 8 ;

FORMAT

'Representativt dygn'n YYMMDD10.

Lufttemperatur_min BEST5.

Lufttemperatur_max BEST4. ;

INFORMAT

'Representativt dygn'n YYMMDD10.

Lufttemperatur_min BEST5.

Lufttemperatur_max BEST4. ;

INFILE '/opt/sas/saswork/SAS_work983400005AA0_sllscip6a/#LN00027'

LRECL=21

ENCODING="LATIN1"

TERMSTR=CRLF

DLM='7F'x

MISSOVER

DSD ;

INPUT

'Representativt dygn'n : ?? YYMMDD10.

Lufttemperatur_min : ?? COMMA5.

Lufttemperatur_max : ?? COMMA4. ;

RUN;

%LET _CLIENTTASKLABEL=;

%LET _CLIENTPROCESSFLOWNAME=;

%LET _CLIENTPROJECTPATH=;

%LET _CLIENTPROJECTPATHHOST=;

%LET _CLIENTPROJECTNAME=;

/* START PÅ NOD: Importera data (nederbörd.csv) */

%LET _CLIENTTASKLABEL='Importera data (nederbörd.csv)';

%LET _CLIENTPROCESSFLOWNAME='Processflöde';

%LET _CLIENTPROJECTPATH='C:\Users\4khw\OneDrive - Karolinska Institutet\General\Studie I\Revision II\Analyses\Study I Revision 2.2.egp';

%LET _CLIENTPROJECTPATHHOST='RSTPC00020406';

%LET _CLIENTPROJECTNAME='Study I Revision 2.2.egp';

/* --------------------------------------------------------------------

Kod genererad av en SAS-uppgift

Genererad 07 January 2025 08:38:16

Av uppgift: guiden Importera data

Källfil:

\\gainashsf01.gaia.sll.se\fs_hsf_usr$\4khw\Dokument\Projekt\Dynamic

EMS allocation\Dataanalys\weather

ederbörd.csv

Server: Lokalt filsystem

Output-data: WORK.nederbörd

Server: SASAppUser

Obs! Som föreberedelse inför körning av nedanstående kod har guiden

Importera data använt interna rutiner för att överföra

källdatafilen från det lokala filsystemet till SASAppUser. Det

finns ingen SAS-kod tillgänglig för att representera den här

åtgärden.

-------------------------------------------------------------------- */

DATA WORK.'nederbörd'n;

LENGTH

'Representativt dygn'n 8

'Nederbördsmängd'n 8 ;

FORMAT

'Representativt dygn'n YYMMDD10.

'Nederbördsmängd'n BEST4. ;

INFORMAT

'Representativt dygn'n YYMMDD10.

'Nederbördsmängd'n BEST4. ;

INFILE '/opt/sas/saswork/SAS_work983400005AA0_sllscip6a/#LN00031'

LRECL=15

ENCODING="LATIN1"

TERMSTR=CRLF

DLM='7F'x

MISSOVER

DSD ;

INPUT

'Representativt dygn'n : ?? YYMMDD10.

'Nederbördsmängd'n : ?? COMMA4. ;

RUN;

%LET _CLIENTTASKLABEL=;

%LET _CLIENTPROCESSFLOWNAME=;

%LET _CLIENTPROJECTPATH=;

%LET _CLIENTPROJECTPATHHOST=;

%LET _CLIENTPROJECTNAME=;

/* START PÅ NOD: Importera data (sunperhour.csv) */

%LET _CLIENTTASKLABEL='Importera data (sunperhour.csv)';

%LET _CLIENTPROCESSFLOWNAME='Processflöde';

%LET _CLIENTPROJECTPATH='C:\Users\4khw\OneDrive - Karolinska Institutet\General\Studie I\Revision II\Analyses\Study I Revision 2.2.egp';

%LET _CLIENTPROJECTPATHHOST='RSTPC00020406';

%LET _CLIENTPROJECTNAME='Study I Revision 2.2.egp';

/* --------------------------------------------------------------------

Kod genererad av en SAS-uppgift

Genererad 07 January 2025 08:38:26

Av uppgift: guiden Importera data

Källfil:

\\gainashsf01.gaia.sll.se\fs_hsf_usr$\4khw\Dokument\Projekt\Dynamic

EMS allocation\Dataanalys\weather\sunperhour.csv

Server: Lokalt filsystem

Output-data: WORK.sunperhour

Server: SASAppUser

Obs! Som föreberedelse inför körning av nedanstående kod har guiden

Importera data använt interna rutiner för att överföra

källdatafilen från det lokala filsystemet till SASAppUser. Det

finns ingen SAS-kod tillgänglig för att representera den här

åtgärden.

-------------------------------------------------------------------- */

DATA WORK.sunperhour;

LENGTH

Datum 8

'Tid (UTC)'n 8

Solskenstid 8 ;

FORMAT

Datum YYMMDD10.

'Tid (UTC)'n TIME8.

Solskenstid BEST4. ;

INFORMAT

Datum YYMMDD10.

'Tid (UTC)'n TIME11.

Solskenstid BEST4. ;

INFILE '/opt/sas/saswork/SAS_work983400005AA0_sllscip6a/#LN00034'

LRECL=24

ENCODING="LATIN1"

TERMSTR=CRLF

DLM='7F'x

MISSOVER

DSD ;

INPUT

Datum : ?? YYMMDD10.

'Tid (UTC)'n : ?? TIME8.

Solskenstid : ?? BEST4. ;

RUN;

%LET _CLIENTTASKLABEL=;

%LET _CLIENTPROCESSFLOWNAME=;

%LET _CLIENTPROJECTPATH=;

%LET _CLIENTPROJECTPATHHOST=;

%LET _CLIENTPROJECTNAME=;

/* START PÅ NOD: Importera data (temperature.csv) */

%LET _CLIENTTASKLABEL='Importera data (temperature.csv)';

%LET _CLIENTPROCESSFLOWNAME='Processflöde';

%LET _CLIENTPROJECTPATH='C:\Users\4khw\OneDrive - Karolinska Institutet\General\Studie I\Revision II\Analyses\Study I Revision 2.2.egp';

%LET _CLIENTPROJECTPATHHOST='RSTPC00020406';

%LET _CLIENTPROJECTNAME='Study I Revision 2.2.egp';

/* --------------------------------------------------------------------

Kod genererad av en SAS-uppgift

Genererad 07 January 2025 08:38:33

Av uppgift: guiden Importera data

Källfil:

\\gainashsf01.gaia.sll.se\fs_hsf_usr$\4khw\Dokument\Projekt\Dynamic

EMS allocation\Dataanalys\weather\temperature.csv

Server: Lokalt filsystem

Output-data: WORK.temperature

Server: SASAppUser

Obs! Som föreberedelse inför körning av nedanstående kod har guiden

Importera data använt interna rutiner för att överföra

källdatafilen från det lokala filsystemet till SASAppUser. Det

finns ingen SAS-kod tillgänglig för att representera den här

åtgärden.

-------------------------------------------------------------------- */

DATA WORK.temperature;

LENGTH

'Representativt dygn'n 8

Lufttemperatur 8 ;

FORMAT

'Representativt dygn'n YYMMDD10.

Lufttemperatur BEST5. ;

INFORMAT

'Representativt dygn'n YYMMDD10.

Lufttemperatur BEST5. ;

INFILE '/opt/sas/saswork/SAS_work983400005AA0_sllscip6a/#LN00037'

LRECL=16

ENCODING="LATIN1"

TERMSTR=CRLF

DLM='7F'x

MISSOVER

DSD ;

INPUT

'Representativt dygn'n : ?? YYMMDD10.

Lufttemperatur : ?? COMMA5. ;

RUN;

%LET _CLIENTTASKLABEL=;

%LET _CLIENTPROCESSFLOWNAME=;

%LET _CLIENTPROJECTPATH=;

%LET _CLIENTPROJECTPATHHOST=;

%LET _CLIENTPROJECTNAME=;

/* START PÅ NOD: Importera data (type of weather.csv) */

%LET _CLIENTTASKLABEL='Importera data (type of weather.csv)';

%LET _CLIENTPROCESSFLOWNAME='Processflöde';

%LET _CLIENTPROJECTPATH='C:\Users\4khw\OneDrive - Karolinska Institutet\General\Studie I\Revision II\Analyses\Study I Revision 2.2.egp';

%LET _CLIENTPROJECTPATHHOST='RSTPC00020406';

%LET _CLIENTPROJECTNAME='Study I Revision 2.2.egp';

/* --------------------------------------------------------------------

Kod genererad av en SAS-uppgift

Genererad 07 January 2025 08:38:41

Av uppgift: guiden Importera data

Källfil:

\\gainashsf01.gaia.sll.se\fs_hsf_usr$\4khw\Dokument\Projekt\Dynamic

EMS allocation\Dataanalys\weather\type of weather.csv

Server: Lokalt filsystem

Output-data: WORK.type of weather

Server: SASAppUser

Obs! Som föreberedelse inför körning av nedanstående kod har guiden

Importera data använt interna rutiner för att överföra

källdatafilen från det lokala filsystemet till SASAppUser. Det

finns ingen SAS-kod tillgänglig för att representera den här

åtgärden.

-------------------------------------------------------------------- */

DATA WORK.'type of weather'n;

LENGTH

'Representativt dygn'n 8

'Nederbörd'n $ 23 ;

FORMAT

'Representativt dygn'n YYMMDD10.

'Nederbörd'n $CHAR23. ;

INFORMAT

'Representativt dygn'n YYMMDD10.

'Nederbörd'n $CHAR23. ;

INFILE '/opt/sas/saswork/SAS_work983400005AA0_sllscip6a/#LN00040'

LRECL=34

ENCODING="LATIN1"

TERMSTR=CRLF

DLM='7F'x

MISSOVER

DSD ;

INPUT

'Representativt dygn'n : ?? YYMMDD10.

'Nederbörd'n : $CHAR23. ;

RUN;

%LET _CLIENTTASKLABEL=;

%LET _CLIENTPROCESSFLOWNAME=;

%LET _CLIENTPROJECTPATH=;

%LET _CLIENTPROJECTPATHHOST=;

%LET _CLIENTPROJECTNAME=;

/* START PÅ NOD: Frågebyggare 1 */

%LET _CLIENTTASKLABEL='Frågebyggare 1';

%LET _CLIENTPROCESSFLOWNAME='Processflöde';

%LET _CLIENTPROJECTPATH='C:\Users\4khw\OneDrive - Karolinska Institutet\General\Studie I\Revision II\Analyses\Study I Revision 2.2.egp';

%LET _CLIENTPROJECTPATHHOST='RSTPC00020406';

%LET _CLIENTPROJECTNAME='Study I Revision 2.2.egp';

%_eg_conditional_dropds(J.DEMSI);

PROC SQL;

CREATE TABLE J.DEMSI AS

SELECT t1.incidentid_lopnr,

t1.taskid_lopnr,

t1.rakelid_lopnr,

t1.'Priority set by dispatch'n LABEL='' AS 'Priority set by EMCC'n,

t1.Station,

t1.KOMMUN,

t1.'Receved emergencycall UTC'n,

t1.ResonOfCallToEMCC,

t1.Year FORMAT=COMMA6. AS Year,

t1.Month FORMAT=COMMA6. AS Month,

t1.Weekday FORMAT=COMMA6. AS Weekday,

t1.Hour FORMAT=COMMA6. AS Hour,

t1.'DistancetoArrival km'n FORMAT=COMMA6. AS 'TravelDistanceToPatient_ km'n,

t1.Date,

t1.MissionCancelled,

t3.Lufttemperatur_min FORMAT=COMMA6. AS Airtemperature_min,

t3.Lufttemperatur_max FORMAT=COMMA6. AS Airtemperature_max,

t4.'Nederbördsmängd'n FORMAT=COMMA6. AS AmmountPrecipitation,

t6.Lufttemperatur FORMAT=COMMA6. AS Airtemperature,

t7.'Nederbörd'n AS PrecipitationType,

t2.SUM_of_Resources FORMAT=COMMA6. AS H_SUM_of_Resources,

t2.H_AVG_of_Onscenetime FORMAT=COMMA6. AS H_AVG_of_Onscenetime,

t2.H_AVG_of_Transportationtime FORMAT=COMMA6. AS H_AVG_of_Transportationtime,

t2.H_AVG_of_Deliverytime FORMAT=COMMA6. AS H_AVG_of_Deliverytime,

t2.H_AVG_of_Responsetime FORMAT=COMMA6. AS H_AVG_of_Responsetime,

t2.H_AVG_of_Call_handelingtime FORMAT=COMMA6. AS H_AVG_of_Call_handelingtime,

t2.H_AVG_of_Drivetime FORMAT=COMMA6. AS H_AVG_of_Drivetime,

t2.H_AVG_of_DistArrvalkm FORMAT=COMMA6. AS H_AVG_of_DistArrvalkm,

t2.C_DISTT_of_missions FORMAT=COMMA6. AS H_SUM_of_Missions,

t2.SUM_of_MissionCanc FORMAT=COMMA6. AS H_SUM_of_MissionCanc,

/* ResponseTime */

(CASE

WHEN t1.Responsetime < 0 THEN MEDIAN(t1.Responsetime)

WHEN t1.Responsetime > 36000 THEN MEDIAN(t1.Responsetime)

ELSE t1.Responsetime

END) FORMAT=COMMA6. AS ResponseTime,

/* Onscenetime */

(CASE

WHEN t2.H_AVG_of_Onscenetime NOT BETWEEN 0 AND 36000 THEN MEDIAN(t1.Onscenetime)

ELSE t2.H_AVG_of_Onscenetime

END) FORMAT=COMMA6. AS Onscenetime,

/* Transportationtime */

(CASE

WHEN t1.Transportationtime NOT BETWEEN 0 AND 36000 THEN MEDIAN(t1.Transportationtime)

ELSE t1.Transportationtime

END) FORMAT=COMMA8. AS Transportationtime,

/* Call_handelingtime */

(CASE

WHEN t1.Call_handelingtime NOT BETWEEN 0 AND 36000 THEN MEDIAN(t1.Call_handelingtime)

ELSE t1.Call_handelingtime

END) FORMAT=COMMA8. AS Call_handelingtime,

/* Drivetime */

(CASE

WHEN t1.Drivetime NOT BETWEEN 0 AND 36000 THEN MEDIAN(t1.Drivetime)

ELSE t1.Drivetime

END) FORMAT=COMMA8. AS Drivetime,

/* Deliverytime */

(CASE

WHEN t1.Deliverytime NOT BETWEEN 0 AND 36000 THEN MEDIAN(t1.Deliverytime)

ELSE t1.Deliverytime

END) FORMAT=NUMX8. AS Deliverytime

FROM WORK.QUERY_FOR_DYNEMSALL_TASKLASTVALU t1

INNER JOIN WORK.QUERY_FOR_DYNEMSALL_TASKLAS_0000 t2 ON (t1.Date = t2.Date) AND (t1.Hour = t2.Hour)

LEFT JOIN WORK.MINMAXTEMP t3 ON (t2.Date = t3.'Representativt dygn'n)

LEFT JOIN WORK.SUNPERHOUR t5 ON (t2.Date = t5.Datum) AND (t2.Hour = t5.'Tid (UTC)'n)

LEFT JOIN WORK.'NEDERBÖRD'n t4 ON (t2.Date = t4.'Representativt dygn'n)

LEFT JOIN WORK.TEMPERATURE t6 ON (t2.Date = t6.'Representativt dygn'n)

LEFT JOIN WORK.'TYPE OF WEATHER'n t7 ON (t2.Date = t7.'Representativt dygn'n)

WHERE t1.incidentid_lopnr NOT IS MISSING AND t1.taskid_lopnr NOT IS MISSING AND t1.'Priority set by dispatch'n IN

(

'1',

'2',

'3'

) AND t1.Responsetime NOT IS MISSING AND t1.Year IN

(

2017,

2018,

2019,

2020,

2021,

2022

)

ORDER BY t1.'Receved emergencycall UTC'n,

t1.'Priority set by dispatch'n,

t1.RakelIndex1,

t1.PRIORITETUT,

t1.Responsetime;

QUIT;

%LET _CLIENTTASKLABEL=;

%LET _CLIENTPROCESSFLOWNAME=;

%LET _CLIENTPROJECTPATH=;

%LET _CLIENTPROJECTPATHHOST=;

%LET _CLIENTPROJECTNAME=;

/* START PÅ NOD: Standardize Data */

%LET _CLIENTTASKLABEL='Standardize Data';

%LET _CLIENTPROCESSFLOWNAME='Processflöde';

%LET _CLIENTPROJECTPATH='C:\Users\4khw\OneDrive - Karolinska Institutet\General\Studie I\Revision II\Analyses\Study I Revision 2.2.egp';

%LET _CLIENTPROJECTPATHHOST='RSTPC00020406';

%LET _CLIENTPROJECTNAME='Study I Revision 2.2.egp';

%macro web_drop_table / parmbuff;%mend;

%macro web_open_table / parmbuff;%mend;

ODS GRAPHICS ON;

TITLE;FOOTNOTE;

FOOTNOTE1 "Genererat av SAS (&_SASSERVERNAME, &SYSSCPL) %TRIM(%QSYSFUNC(DATE(), NLDATE20.)) %TRIM(%QSYSFUNC(TIME(), NLTIME.))";

ods noproctitle;

proc stdize data=J.DEMSI method=std nomiss out=WORK.STDIZE oprefix

sprefix=Standardized_;

var 'TravelDistanceToPatient_ km'n MissionCancelled Airtemperature_min

Airtemperature_max AmmountPrecipitation Airtemperature H_SUM_of_Resources

H_AVG_of_Onscenetime H_AVG_of_Transportationtime H_AVG_of_Deliverytime

H_AVG_of_Responsetime H_AVG_of_Call_handelingtime H_AVG_of_Drivetime

H_AVG_of_DistArrvalkm H_SUM_of_Missions H_SUM_of_MissionCanc ResponseTime;

run;

TITLE;FOOTNOTE;

%LET _CLIENTTASKLABEL=;

%LET _CLIENTPROCESSFLOWNAME=;

%LET _CLIENTPROJECTPATH=;

%LET _CLIENTPROJECTPATHHOST=;

%LET _CLIENTPROJECTNAME=;

/* START PÅ NOD: Describe Missing Data */

%LET _CLIENTTASKLABEL='Describe Missing Data';

%LET _CLIENTPROCESSFLOWNAME='Processflöde';

%LET _CLIENTPROJECTPATH='C:\Users\4khw\OneDrive - Karolinska Institutet\General\Studie I\Revision II\Analyses\Study I Revision 2.2.egp';

%LET _CLIENTPROJECTPATHHOST='RSTPC00020406';

%LET _CLIENTPROJECTNAME='Study I Revision 2.2.egp';

%macro web_drop_table / parmbuff;%mend;

%macro web_open_table / parmbuff;%mend;

ODS GRAPHICS ON;

TITLE;FOOTNOTE;

FOOTNOTE1 "Genererat av SAS (&_SASSERVERNAME, &SYSSCPL) %TRIM(%QSYSFUNC(DATE(), NLDATE20.)) %TRIM(%QSYSFUNC(TIME(), NLTIME.))";

ods noproctitle;

proc format;

value _nmissprint low-high="Non-missing";

value $_cmissprint " "=" " other="Non-missing";

run;

proc freq data=J.DEMSI;

title3 "Missing Data Frequencies";

title4 h=2 "Legend: ., A, B, etc = Missing";

format incidentid_lopnr taskid_lopnr rakelid_lopnr

'Receved emergencycall UTC'n Year Month Weekday Hour

'TravelDistanceToPatient_ km'n Call_handelingtime Drivetime Date

MissionCancelled Onscenetime Transportationtime Deliverytime

Airtemperature_min Airtemperature_max AmmountPrecipitation Airtemperature

H_SUM_of_Resources H_AVG_of_Onscenetime H_AVG_of_Transportationtime

H_AVG_of_Deliverytime H_AVG_of_Responsetime H_AVG_of_Call_handelingtime

H_AVG_of_Drivetime H_AVG_of_DistArrvalkm H_SUM_of_Missions

H_SUM_of_MissionCanc ResponseTime _nmissprint.;

format 'Priority set by EMCC'n Station KOMMUN PrecipitationType $_cmissprint.;

tables incidentid_lopnr taskid_lopnr rakelid_lopnr 'Priority set by EMCC'n

Station KOMMUN 'Receved emergencycall UTC'n Year Month Weekday Hour

'TravelDistanceToPatient_ km'n Call_handelingtime Drivetime Date

MissionCancelled Onscenetime Transportationtime Deliverytime

Airtemperature_min Airtemperature_max AmmountPrecipitation Airtemperature

PrecipitationType H_SUM_of_Resources H_AVG_of_Onscenetime

H_AVG_of_Transportationtime H_AVG_of_Deliverytime H_AVG_of_Responsetime

H_AVG_of_Call_handelingtime H_AVG_of_Drivetime H_AVG_of_DistArrvalkm

H_SUM_of_Missions H_SUM_of_MissionCanc ResponseTime / missing nocum;

run;

proc freq data=J.DEMSI noprint;

table incidentid_lopnr * taskid_lopnr * rakelid_lopnr *

'Priority set by EMCC'n * Station * KOMMUN * 'Receved emergencycall UTC'n *

Year * Month * Weekday * Hour * 'TravelDistanceToPatient_ km'n *

Call_handelingtime * Drivetime * Date * MissionCancelled * Onscenetime *

Transportationtime * Deliverytime * Airtemperature_min * Airtemperature_max *

AmmountPrecipitation * Airtemperature * PrecipitationType *

H_SUM_of_Resources * H_AVG_of_Onscenetime * H_AVG_of_Transportationtime *

H_AVG_of_Deliverytime * H_AVG_of_Responsetime * H_AVG_of_Call_handelingtime *

H_AVG_of_Drivetime * H_AVG_of_DistArrvalkm * H_SUM_of_Missions *

H_SUM_of_MissionCanc * ResponseTime / missing out=Work._MissingData_;

format incidentid_lopnr taskid_lopnr rakelid_lopnr

'Receved emergencycall UTC'n Year Month Weekday Hour

'TravelDistanceToPatient_ km'n Call_handelingtime Drivetime Date

MissionCancelled Onscenetime Transportationtime Deliverytime

Airtemperature_min Airtemperature_max AmmountPrecipitation Airtemperature

H_SUM_of_Resources H_AVG_of_Onscenetime H_AVG_of_Transportationtime

H_AVG_of_Deliverytime H_AVG_of_Responsetime H_AVG_of_Call_handelingtime

H_AVG_of_Drivetime H_AVG_of_DistArrvalkm H_SUM_of_Missions

H_SUM_of_MissionCanc ResponseTime _nmissprint.;

format 'Priority set by EMCC'n Station KOMMUN PrecipitationType $_cmissprint.;

run;

proc print data=Work._MissingData_ noobs label;

title3 "Missing Data Patterns across Variables";

title4 h=2 "Legend: ., A, B, etc = Missing";

format incidentid_lopnr taskid_lopnr rakelid_lopnr

'Receved emergencycall UTC'n Year Month Weekday Hour

'TravelDistanceToPatient_ km'n Call_handelingtime Drivetime Date

MissionCancelled Onscenetime Transportationtime Deliverytime

Airtemperature_min Airtemperature_max AmmountPrecipitation Airtemperature

H_SUM_of_Resources H_AVG_of_Onscenetime H_AVG_of_Transportationtime

H_AVG_of_Deliverytime H_AVG_of_Responsetime H_AVG_of_Call_handelingtime

H_AVG_of_Drivetime H_AVG_of_DistArrvalkm H_SUM_of_Missions

H_SUM_of_MissionCanc ResponseTime _nmissprint.;

format 'Priority set by EMCC'n Station KOMMUN PrecipitationType $_cmissprint.;

label count="Frequency" percent="Percent";

run;

title3;

/* Clean up */

proc delete data=Work._MissingData_;

run;

TITLE;FOOTNOTE;

%LET _CLIENTTASKLABEL=;

%LET _CLIENTPROCESSFLOWNAME=;

%LET _CLIENTPROJECTPATH=;

%LET _CLIENTPROJECTPATHHOST=;

%LET _CLIENTPROJECTNAME=;

;*';*";*/;quit;run;

ODS _ALL_ CLOSE;

### SAS code from analyses in SAS Viya Visual Analytics 8.5.2

#### Gradient Boosting overall

/*---------------------------------------------------------

The options statement below should be placed

before the data step when submitting this code.

---------------------------------------------------------*/

options VALIDMEMNAME=EXTEND VALIDVARNAME=ANY;

/*---------------------------------------------------------

Before this code can run you need to fill in all the

macro variables below.

---------------------------------------------------------*/

/*---------------------------------------------------------

Start Macro Variables

---------------------------------------------------------*/

%let SOURCE_HOST=<Hostname>; /* The host name of the CAS server */

%let SOURCE_PORT=<Port>; /* The port of the CAS server */

%let SOURCE_LIB=<Library>; /* The CAS library where the source data resides */

%let SOURCE_DATA=<Tablename>; /* The CAS table name of the source data */

%let DEST_LIB=<Library>; /* The CAS library where the destination data should go */

%let DEST_DATA=<Tablename>; /* The CAS table name where the destination data should go */

/* Open a CAS session and make the CAS libraries available */

options cashost="&SOURCE_HOST" casport=&SOURCE_PORT;

cas mysess;

caslib _all_ assign;

/* Load ASTOREs into CAS memory */

proc casutil;

Load casdata="Gradient_boosting___Response_Time_(minutes)_1.sashdat" incaslib="Models" casout="Gradient_boosting___Response_Time_(minutes)_1" outcaslib="casuser" replace;

Quit;

/* Apply the model */

proc cas;

fcmpact.runProgram /

inputData={caslib="&SOURCE_LIB" name="&SOURCE_DATA"}

outputData={caslib="&DEST_LIB" name="&DEST_DATA" replace=1}

routineCode = "

/*------------------------------------------

Generated SAS Scoring Code

Date : 17Dec2024:15:25:16

Locale : en_US

Model Type : Gradient Boosting

Interval variable: _va_d_Response_Time_(minutes)(Response Time (minutes))

Interval variable: Airtemperature

Interval variable: Airtemperature_max

Interval variable: Airtemperature_min

Interval variable: AmmountPrecipitation

Interval variable: H_AVG_of_DistArrvalkm

Interval variable: H_SUM_of_MissionCanc

Interval variable: H_SUM_of_Missions

Interval variable: H_SUM_of_Resources

Interval variable: Hour

Interval variable: Month

Interval variable: H_AVG_of_Call_handelingtime

Interval variable: H_AVG_of_Deliverytime

Interval variable: H_AVG_of_Drivetime

Interval variable: H_AVG_of_Onscenetime

Interval variable: H_AVG_of_Responsetime

Interval variable: H_AVG_of_Transportationtime

Interval variable: TravelDistanceToPatient_ km

Class variable : PrecipitationType

Class variable : Priority set by EMCC

Class variable : Station(EMS Station)

Class variable : KOMMUN(Locality)

Class variable : _va_d_Weekday_ONES(Weekday)

Class variable : ResonOfCallToEMCC(ReasonOfCallToEMCC)

Response variable: _va_d_Response_Time_(minutes)(Response Time (minutes))

------------------------------------------*/

/* Temporary Computed Columns */

'_va_d_Response_Time_(minutes)'n=('ResponseTime'n / 60);;

'_va_d_Weekday_ONES'n=round('Weekday'n,1);

/*------------------------------------------*/

declare object Gradient_boosting___Response_Time_(minutes)_1(astore);

call Gradient_boosting___Response_Time_(minutes)_1.score('CASUSER','Gradient_boosting___Response_Time_(minutes)_1');

/*------------------------------------------*/

/*_VA_DROP*/ drop '_va_d_Response_Time_(minutes)'n '_va_d_Weekday_ONES'n 'P__va_d_Response_Time__minutes_'n;

'P__va_d_Response_Time__minu_57'n='P__va_d_Response_Time__minutes_'n;

/*------------------------------------------*/

";

run;

Quit;

/* Persist the output table */

proc casutil;

Save casdata="&DEST_DATA" incaslib="&DEST_LIB" casout="&DEST_DATA%str(.)sashdat" outcaslib="&DEST_LIB" replace;

Quit;

#### Gradient Boosting priority level 1

/*---------------------------------------------------------

The options statement below should be placed

before the data step when submitting this code.

---------------------------------------------------------*/

options VALIDMEMNAME=EXTEND VALIDVARNAME=ANY;

/*---------------------------------------------------------

Before this code can run you need to fill in all the

macro variables below.

---------------------------------------------------------*/

/*---------------------------------------------------------

Start Macro Variables

---------------------------------------------------------*/

%let SOURCE_HOST=<Hostname>; /* The host name of the CAS server */

%let SOURCE_PORT=<Port>; /* The port of the CAS server */

%let SOURCE_LIB=<Library>; /* The CAS library where the source data resides */

%let SOURCE_DATA=<Tablename>; /* The CAS table name of the source data */

%let DEST_LIB=<Library>; /* The CAS library where the destination data should go */

%let DEST_DATA=<Tablename>; /* The CAS table name where the destination data should go */

/* Open a CAS session and make the CAS libraries available */

options cashost="&SOURCE_HOST" casport=&SOURCE_PORT;

cas mysess;

caslib _all_ assign;

/* Load ASTOREs into CAS memory */

proc casutil;

Load casdata="Gradient_boosting___Response_Time_(minutes)_2.sashdat" incaslib="Models" casout="Gradient_boosting___Response_Time_(minutes)_2" outcaslib="casuser" replace;

Quit;

/* Apply the model */

proc cas;

fcmpact.runProgram /

inputData={caslib="&SOURCE_LIB" name="&SOURCE_DATA"}

outputData={caslib="&DEST_LIB" name="&DEST_DATA" replace=1}

routineCode = "

/*------------------------------------------

Generated SAS Scoring Code

Date : 17Dec2024:15:16:53

Locale : en_US

Model Type : Gradient Boosting

Interval variable: _va_d_Response_Time_(minutes)(Response Time (minutes))

Interval variable: Airtemperature

Interval variable: Airtemperature_max

Interval variable: Airtemperature_min

Interval variable: AmmountPrecipitation

Interval variable: H_AVG_of_DistArrvalkm

Interval variable: H_SUM_of_MissionCanc

Interval variable: H_SUM_of_Missions

Interval variable: H_SUM_of_Resources

Interval variable: Hour

Interval variable: Month

Interval variable: H_AVG_of_Call_handelingtime

Interval variable: H_AVG_of_Deliverytime

Interval variable: H_AVG_of_Drivetime

Interval variable: H_AVG_of_Onscenetime

Interval variable: H_AVG_of_Responsetime

Interval variable: H_AVG_of_Transportationtime

Interval variable: TravelDistanceToPatient_ km

Class variable : PrecipitationType

Class variable : Priority set by EMCC

Class variable : Station(EMS Station)

Class variable : KOMMUN(Locality)

Class variable : _va_d_Weekday_ONES(Weekday)

Class variable : ResonOfCallToEMCC(ReasonOfCallToEMCC)

Response variable: _va_d_Response_Time_(minutes)(Response Time (minutes))

------------------------------------------*/

/* Temporary Computed Columns */

'_va_d_Response_Time_(minutes)'n=('ResponseTime'n / 60);;

'_va_d_Weekday_ONES'n=round('Weekday'n,1);

/*------------------------------------------*/

declare object Gradient_boosting___Response_Time_(minutes)_2(astore);

call Gradient_boosting___Response_Time_(minutes)_2.score('CASUSER','Gradient_boosting___Response_Time_(minutes)_2');

/*------------------------------------------*/

/*_VA_DROP*/ drop '_va_d_Response_Time_(minutes)'n '_va_d_Weekday_ONES'n 'P__va_d_Response_Time__minutes_'n;

'P__va_d_Response_Time__min_981'n='P__va_d_Response_Time__minutes_'n;

/*------------------------------------------*/

";

run;

Quit;

/* Persist the output table */

proc casutil;

Save casdata="&DEST_DATA" incaslib="&DEST_LIB" casout="&DEST_DATA%str(.)sashdat" outcaslib="&DEST_LIB" replace;

Quit;

#### Gradient Boosting priority level 2

/*---------------------------------------------------------

The options statement below should be placed

before the data step when submitting this code.

---------------------------------------------------------*/

options VALIDMEMNAME=EXTEND VALIDVARNAME=ANY;

/*---------------------------------------------------------

Before this code can run you need to fill in all the

macro variables below.

---------------------------------------------------------*/

/*---------------------------------------------------------

Start Macro Variables

---------------------------------------------------------*/

%let SOURCE_HOST=<Hostname>; /* The host name of the CAS server */

%let SOURCE_PORT=<Port>; /* The port of the CAS server */

%let SOURCE_LIB=<Library>; /* The CAS library where the source data resides */

%let SOURCE_DATA=<Tablename>; /* The CAS table name of the source data */

%let DEST_LIB=<Library>; /* The CAS library where the destination data should go */

%let DEST_DATA=<Tablename>; /* The CAS table name where the destination data should go */

/* Open a CAS session and make the CAS libraries available */

options cashost="&SOURCE_HOST" casport=&SOURCE_PORT;

cas mysess;

caslib _all_ assign;

/* Load ASTOREs into CAS memory */

proc casutil;

Load casdata="Gradient_boosting___Response_Time_(minutes)_3.sashdat" incaslib="Models" casout="Gradient_boosting___Response_Time_(minutes)_3" outcaslib="casuser" replace;

Quit;

/* Apply the model */

proc cas;

fcmpact.runProgram /

inputData={caslib="&SOURCE_LIB" name="&SOURCE_DATA"}

outputData={caslib="&DEST_LIB" name="&DEST_DATA" replace=1}

routineCode = "

/*------------------------------------------

Generated SAS Scoring Code

Date : 17Dec2024:15:22:42

Locale : en_US

Model Type : Gradient Boosting

Interval variable: _va_d_Response_Time_(minutes)(Response Time (minutes))

Interval variable: Airtemperature

Interval variable: Airtemperature_max

Interval variable: Airtemperature_min

Interval variable: AmmountPrecipitation

Interval variable: H_AVG_of_DistArrvalkm

Interval variable: H_SUM_of_MissionCanc

Interval variable: H_SUM_of_Missions

Interval variable: H_SUM_of_Resources

Interval variable: Hour

Interval variable: Month

Interval variable: H_AVG_of_Call_handelingtime

Interval variable: H_AVG_of_Deliverytime

Interval variable: H_AVG_of_Drivetime

Interval variable: H_AVG_of_Onscenetime

Interval variable: H_AVG_of_Responsetime

Interval variable: H_AVG_of_Transportationtime

Interval variable: TravelDistanceToPatient_ km

Class variable : PrecipitationType

Class variable : Priority set by EMCC

Class variable : Station(EMS Station)

Class variable : KOMMUN(Locality)

Class variable : _va_d_Weekday_ONES(Weekday)

Class variable : ResonOfCallToEMCC(ReasonOfCallToEMCC)

Response variable: _va_d_Response_Time_(minutes)(Response Time (minutes))

------------------------------------------*/

/* Temporary Computed Columns */

'_va_d_Response_Time_(minutes)'n=('ResponseTime'n / 60);;

'_va_d_Weekday_ONES'n=round('Weekday'n,1);

/*------------------------------------------*/

declare object Gradient_boosting___Response_Time_(minutes)_3(astore);

call Gradient_boosting___Response_Time_(minutes)_3.score('CASUSER','Gradient_boosting___Response_Time_(minutes)_3');

/*------------------------------------------*/

/*_VA_DROP*/ drop '_va_d_Response_Time_(minutes)'n '_va_d_Weekday_ONES'n 'P__va_d_Response_Time__minutes_'n;

'P__va_d_Response_Time__mi_1502'n='P__va_d_Response_Time__minutes_'n;

/*------------------------------------------*/

";

run;

Quit;

/* Persist the output table */

proc casutil;

Save casdata="&DEST_DATA" incaslib="&DEST_LIB" casout="&DEST_DATA%str(.)sashdat" outcaslib="&DEST_LIB" replace;

Quit;

#### Gradient Boosting priority level 3

/*---------------------------------------------------------

The options statement below should be placed

before the data step when submitting this code.

---------------------------------------------------------*/

options VALIDMEMNAME=EXTEND VALIDVARNAME=ANY;

/*---------------------------------------------------------

Before this code can run you need to fill in all the

macro variables below.

---------------------------------------------------------*/

/*---------------------------------------------------------

Start Macro Variables

---------------------------------------------------------*/

%let SOURCE_HOST=<Hostname>; /* The host name of the CAS server */

%let SOURCE_PORT=<Port>; /* The port of the CAS server */

%let SOURCE_LIB=<Library>; /* The CAS library where the source data resides */

%let SOURCE_DATA=<Tablename>; /* The CAS table name of the source data */

%let DEST_LIB=<Library>; /* The CAS library where the destination data should go */

%let DEST_DATA=<Tablename>; /* The CAS table name where the destination data should go */

/* Open a CAS session and make the CAS libraries available */

options cashost="&SOURCE_HOST" casport=&SOURCE_PORT;

cas mysess;

caslib _all_ assign;

/* Load ASTOREs into CAS memory */

proc casutil;

Load casdata="Gradient_boosting___Response_Time_(minutes)_4.sashdat" incaslib="Models" casout="Gradient_boosting___Response_Time_(minutes)_4" outcaslib="casuser" replace;

Quit;

/* Apply the model */

proc cas;

fcmpact.runProgram /

inputData={caslib="&SOURCE_LIB" name="&SOURCE_DATA"}

outputData={caslib="&DEST_LIB" name="&DEST_DATA" replace=1}

routineCode = "

/*------------------------------------------

Generated SAS Scoring Code

Date : 17Dec2024:15:20:38

Locale : en_US

Model Type : Gradient Boosting

Interval variable: _va_d_Response_Time_(minutes)(Response Time (minutes))

Interval variable: Airtemperature

Interval variable: Airtemperature_max

Interval variable: Airtemperature_min

Interval variable: AmmountPrecipitation

Interval variable: H_AVG_of_DistArrvalkm

Interval variable: H_SUM_of_MissionCanc

Interval variable: H_SUM_of_Missions

Interval variable: H_SUM_of_Resources

Interval variable: Hour

Interval variable: Month

Interval variable: H_AVG_of_Call_handelingtime

Interval variable: H_AVG_of_Deliverytime

Interval variable: H_AVG_of_Drivetime

Interval variable: H_AVG_of_Onscenetime

Interval variable: H_AVG_of_Responsetime

Interval variable: H_AVG_of_Transportationtime

Interval variable: TravelDistanceToPatient_ km

Class variable : PrecipitationType

Class variable : Priority set by EMCC

Class variable : Station(EMS Station)

Class variable : KOMMUN(Locality)

Class variable : _va_d_Weekday_ONES(Weekday)

Class variable : ResonOfCallToEMCC(ReasonOfCallToEMCC)

Response variable: _va_d_Response_Time_(minutes)(Response Time (minutes))

------------------------------------------*/

/* Temporary Computed Columns */

'_va_d_Response_Time_(minutes)'n=('ResponseTime'n / 60);;

'_va_d_Weekday_ONES'n=round('Weekday'n,1);

/*------------------------------------------*/

declare object Gradient_boosting___Response_Time_(minutes)_4(astore);

call Gradient_boosting___Response_Time_(minutes)_4.score('CASUSER','Gradient_boosting___Response_Time_(minutes)_4');

/*------------------------------------------*/

/*_VA_DROP*/ drop '_va_d_Response_Time_(minutes)'n '_va_d_Weekday_ONES'n 'P__va_d_Response_Time__minutes_'n;

'P__va_d_Response_Time__mi_1987'n='P__va_d_Response_Time__minutes_'n;

/*------------------------------------------*/

";

run;

Quit;

/* Persist the output table */

proc casutil;

Save casdata="&DEST_DATA" incaslib="&DEST_LIB" casout="&DEST_DATA%str(.)sashdat" outcaslib="&DEST_LIB" replace;

Quit;
